# Advances in Magnetic Nanoparticles-Supported Palladium Complexes for Coupling Reactions

**DOI:** 10.3390/molecules23102532

**Published:** 2018-10-04

**Authors:** Mahmoud Nasrollahzadeh

**Affiliations:** Department of Chemistry, Faculty of Science, University of Qom, Qom 37185-359, Iran; mahmoudnasr81@gmail.com; Tel.: +98-25-32850953; Fax: +98-25-32103595

**Keywords:** magnetic nanoparticles, palladium complexes, coupling reactions

## Abstract

Carbon‒carbon (C‒C) and carbon‒heteroatom (C‒X) bonds that form via transition-metal-catalyzed processes have been extensively used in the organic synthesis and preparation of natural products and important compounds such as heterocycles, biologically active molecules, and dendrimers. Among the most significant catalysts, magnetic nanoparticles-supported palladium complexes are very effective, versatile, and heterogeneous catalysts for a wide range of C‒C and C‒X coupling reactions due to their reusability, thermal stability, and excellent catalytic performance. In this review, recent advances to develop magnetic nanoparticles supported palladium complexes, including their preparation, characterization, catalytic application, and reusability in the formation of both C‒C and C‒X bonds, by authors such as Sonogashira, Heck, Suzuki‒Miyaura, and Stille, and a few examples concerning *N*-arylation, *S*-arylation, and C_sp2_-P coupling reactions are discussed.

## 1. Introduction

Over the past 15 years, there has been growing interest in coupling reactions, one of the most powerful tools for C‒C and C‒X bond formation. C‒C and C‒X coupling reactions are widely utilized to assemble a vast range of important molecules in pharmaceutical, agricultural, natural products, non-linear optical materials, polymeric materials, molecular electronics, polyalkynylated molecules, and macrocycles with acetylene links [1,2,3,4,5,6,7,8,9,10,11,12]. There are a variety of studies on C‒C and C‒X coupling reactions with transition-metal-based catalysts. 

During the past decade, there has been significant interest in improving experimental procedures for coupling reactions using various homogeneous metallic complexes. However, most of these catalysts are homogeneous and cannot be recovered from the reaction medium. Generally, heterogeneous catalysis is favored over homogeneous catalysis due to their ease of handling, reusability, and regenerability.

Among the catalysts or heterogeneous supports, metal or metal oxide nanoparticles (NPs) often showed high catalytic activity different from the corresponding bulk materials because of their different shapes and sizes and high surface-to-volume ratio, which gives rise to distinctive quantum properties. However, to avoid the agglomeration of the NPs or reusability of metallic complexes, the discovery of a safer, nontoxic, ecofriendly, and recyclable support is still in demand for coupling reactions in heterogeneous conditions.

**Scheme 1:**
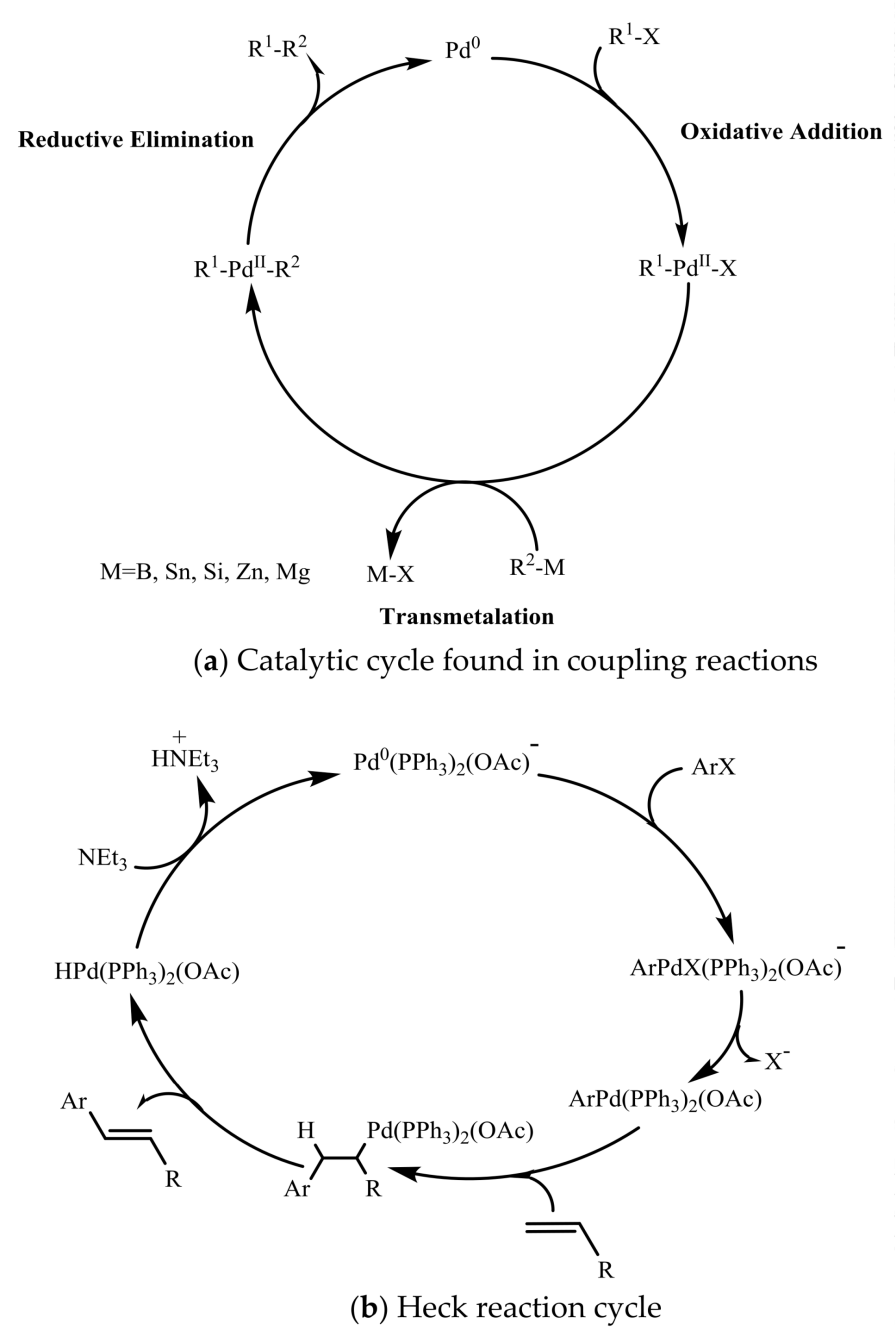
Major steps of Pd-catalyzed coupling reactions [1].

In this regard, various solid supports have been introduced to prevent the agglomeration of NPs or reusability of metallic complexes and to facilitate catalyst separation and recycling. It is also well known that metallic nanocomposite-based magnetic supports are popular with scientists due to their chemical stability and high specific surface area [13,14,15,16,17,18,19,20]. Also, they do not swell in organic solvents and can be recovered from the reaction mixture with an external magnetic field and reused several times with considerable efficiency. Recently, magnetic materials have attracted increasing attention in the immobilization of palladium catalysts for coupling reactions. In general, carbon‒carbon coupling reactions catalyzed by solid-supported Pd follow the usual reaction mechanism, as shown in Scheme 1 [1].

## 2. Novel Types of Magnetic Nanoparticles-Supported Palladium Complexes for C‒C and C‒X Coupling Reactions

There are many reports about the synthesis of magnetic nanoparticles-supported palladium complexes that used as effective catalysts for the formation C‒C and C‒X bonds via coupling reactions.

### 2.1. Direct Immobilization on Magnetic Nanoparticles

**Scheme 2:**
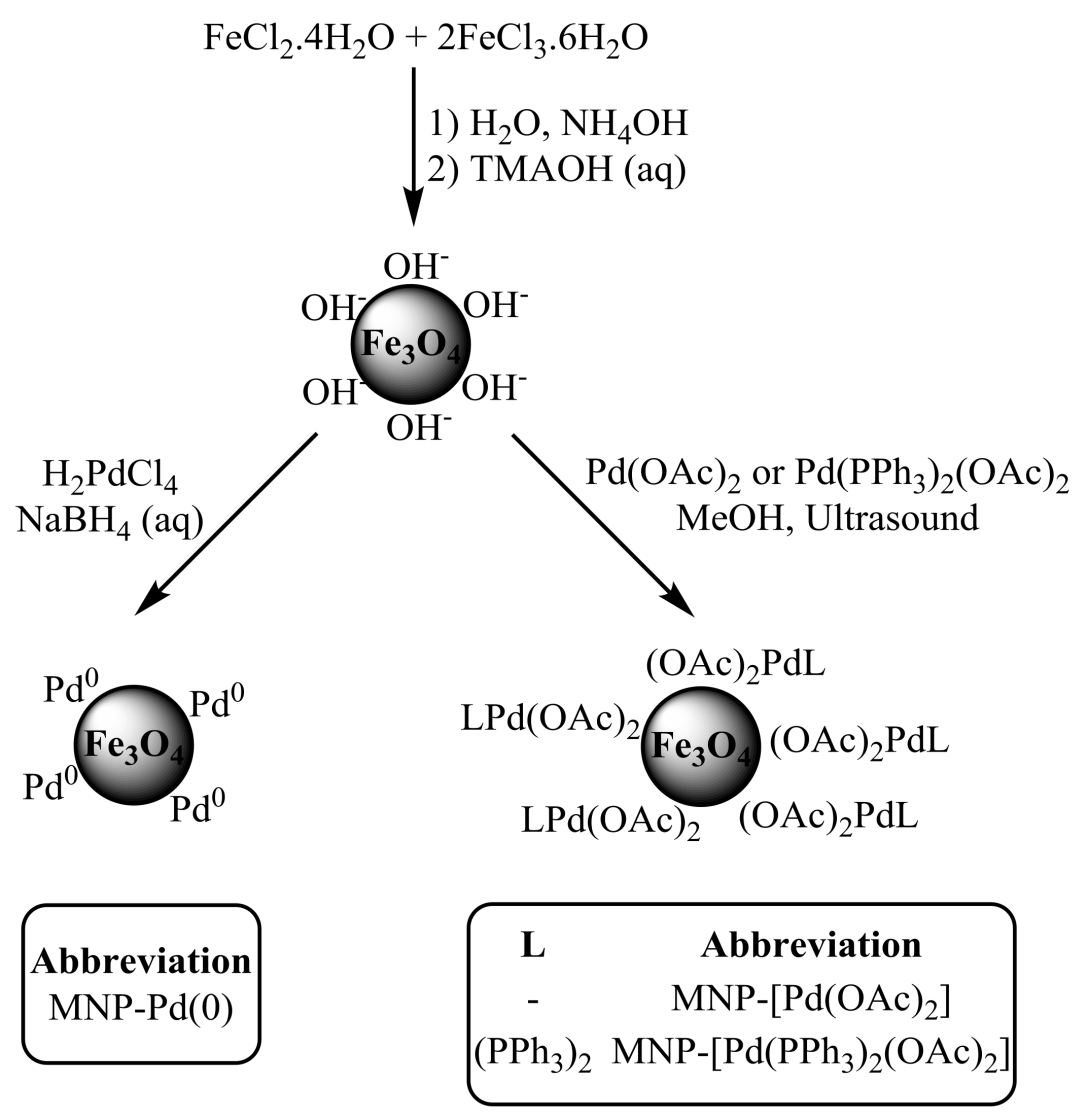
The preparation of MNP-supported palladium catalysts. Reproduced with permission from [21].

One of the simplest methods for the preparation of magnetically separable palladium catalysts is the direct immobilization of Pd NPs on the surfaces of magnetic materials; in this regard, Pd NPs are stabilized on un-functionalized Fe_3_O_4_ NPs. Indeed, this method has focused on combining high activity and selectivity of homogeneous species with ease of separation and recycling of heterogeneous catalysts. An example of this type of magnetically separable Pd-catalysts in C‒C coupling reactions is a study by Plucinski and co-workers in 2009; they prepared a series of palladium-based catalysts supported on magnetic NPs and evaluated the catalytic activities of the resulting catalysts in C‒C coupling, hydrogenation, and amination reactions [21]. The Fe_3_O_4_ NPs was synthesized through co-precipitation of ferric and ferrous ions, adding tetramethylammonium hydroxide (TMAOH) to make a negative charge on the surface; following the addition of Pd salts, catalysts were prepared. The Fe_3_O_4_-Pd^0^ catalyst was synthesized by reduction of H_2_PdCl_4_ using NaBH_4_ and Fe_3_O_4_-Pd(OAc)_2_ and Fe_3_O_4_-Pd(PPh_3_)_2_(OAc)_2_ catalysts were prepared from Pd(OAc)_2_ and Pd(PPh_3_)_2_(OAc)_2_ salts, respectively (Scheme 2).

**Scheme 3:**
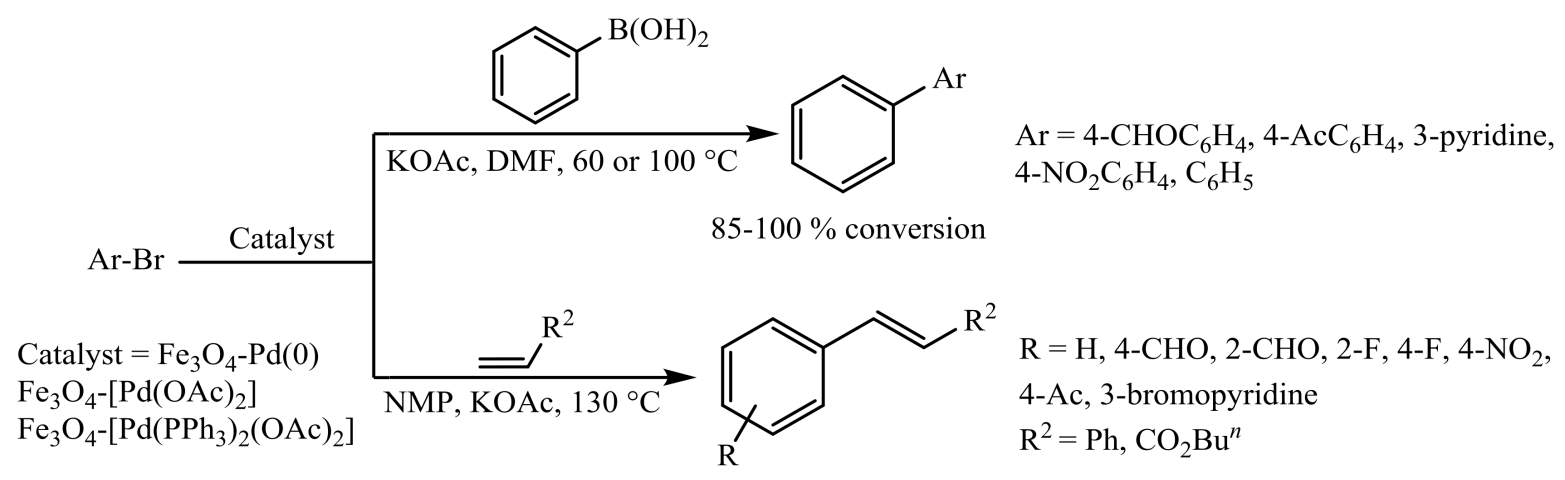
Heck and Suzuki‒Miyaura coupling reactions catalyzed by Fe_3_O_4_-Pd(OAc)_2_, Fe_3_O_4_-Pd^0^, and Fe_3_O_4_ Pd(PPh_3_)_2_(OAc)_2_ [21].

The Suzuki‒Miyaura coupling of phenylboronic acid with bromobenzene in DMF at 60 °C in the presence of Fe_3_O_4_-Pd(OAc)_2_, Fe_3_O_4_-Pd^0^ and Fe_3_O_4_-Pd(PPh_3_)_2_(OAc)_2_ as the catalyst showed 85%, 54%, and 45% yield, respectively. Also, Fe_3_O_4_-Pd(OAc)_2_ was used in the Suzuki‒Miyaura coupling reaction of various (hetero)aryl bromides with phenylboronic acid in DMF at 100 °C. In addition, the application of all catalysts in a Heck coupling reaction in the presence of potassium acetate as a base in NMP at 130 °C was evaluated (Scheme 3). A recycling experiment for Fe_3_O_4_-Pd^0^ and Fe_3_O_4_-[Pd(OAc)_2_] showed high activity; however, in the case of the Fe_3_O_4_-[Pd(PPh_3_)_2_(OAc)_2_] system, the conversion markedly decreases after the second run.

### 2.2. Core-Shell Magnetic Nps with an Inorganic Shell

**Figure 1:**
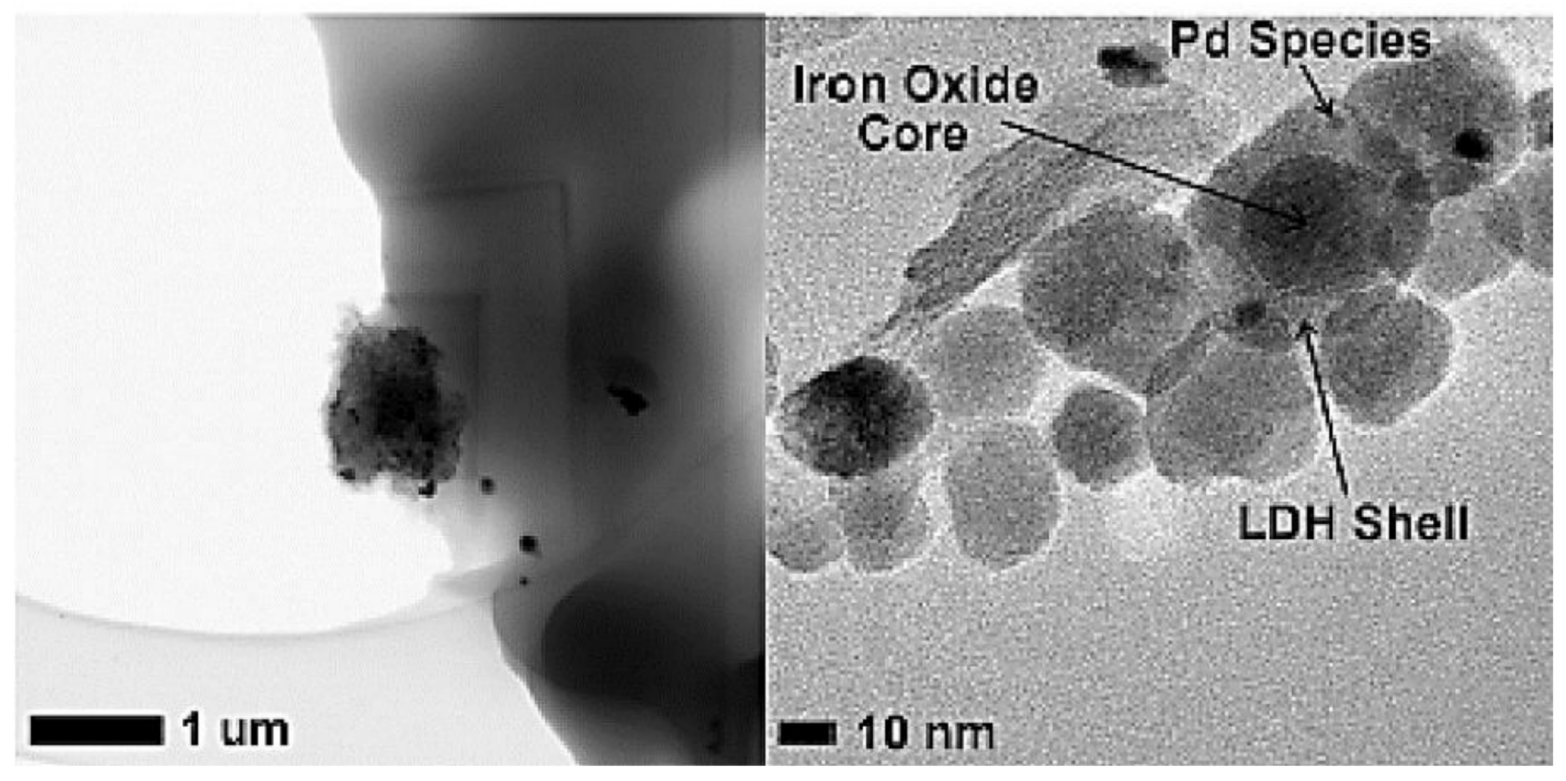
TEM images of the Pd-LDH@M catalyst. Reproduced with permission from [22].

**Scheme 4:**
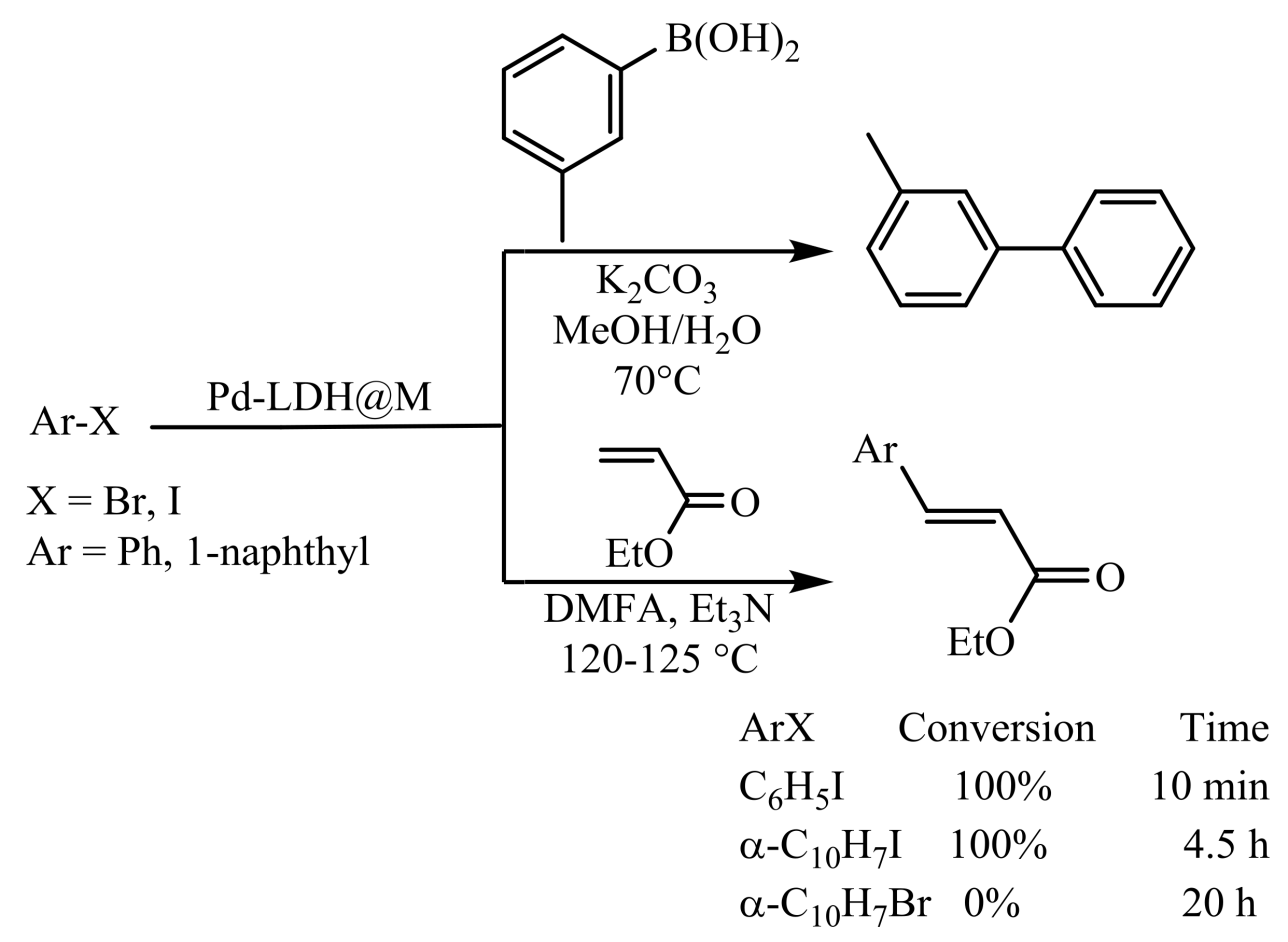
Suzuki‒Miyaura and Heck reaction of aryl halides catalyzed by Pd-LDH@M [22].

One of the methods used to develop magnetically separable catalysts is encapsulation of magnetic NPs by inorganic materials such as metal oxides. Along this line, Sokolov and Karan have prepared a catalyst with a magnetite core and Mg‒Al-layered double hydroxide shell functionalized with a palladium(0) complex (Pd_2_(dba)_3_ in which dba = dibenzylideneacetone) [22]. Spherical particles with diameters about 10–40 nm were revealed in TEM images (Figure 1). Suzuki‒Miyaura reaction of aryl halides with arylboronic acid in H_2_O/MeOH at 70 °C was performed in the presence of Pd-LDH@M catalyst and gave product in good yield. Also, the catalyst showed high activity in the Heck coupling of aryl iodides (Scheme 4). The catalyst could be separated due to its ferromagnetic behavior from the reaction mixture by means of magnetic field. The activity of the reused catalyst in the next run decreased markedly. The authors believe that the interaction of Pd with hydroxide shell is not strong enough and leaching of Pd^0^ from this support is not unexpected, thus the catalyst has limited recyclability.

### 2.3. Magnetic Polymer Nanocomposites

**Scheme 5:**
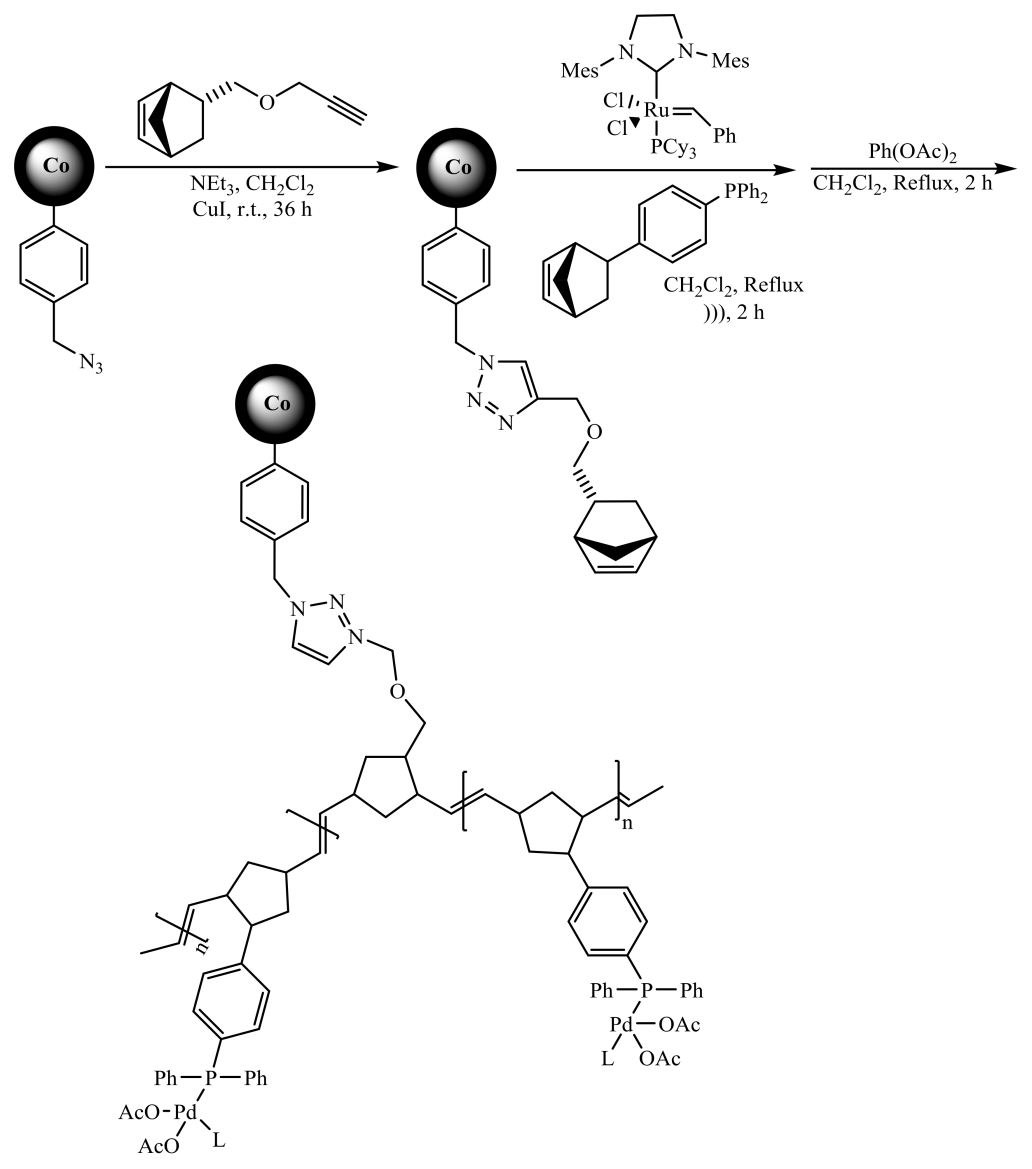
Formation of a heterogeneous palladium complex on PPh_3_-functionalized Co/C-ROMPgel. Reproduced with permission from [23].

Coating the surfaces of magnetic NPs with polymers provides another way to stabilize metal NPs on the surfaces of magnetic materials. In addition, since a decrease in activity and selectivity often appeared due to the low solubility of the supported catalysts in the reaction media, by using various hydrophobic and hydrophilic polymeric coatings, the solubility of the catalyst in both organic and aqueous solvents is markedly enhanced. In this regard, Co-based magnetic NPs functionalized with a polymeric phosphine ligand were synthesized by Reiser and Hanson [23]. The grafting of this polymeric ligand onto carbon-coated cobalt magnetic NPs was based on a “click” reaction, followed by ring-opening metathesis polymerization (ROMP). Treating PPh_3_-functionalized Co/C magnetic NPs with Pd(OAc)_2_ results in the generation of a recyclable Pd catalyst (Scheme 5).

**Scheme 6:**
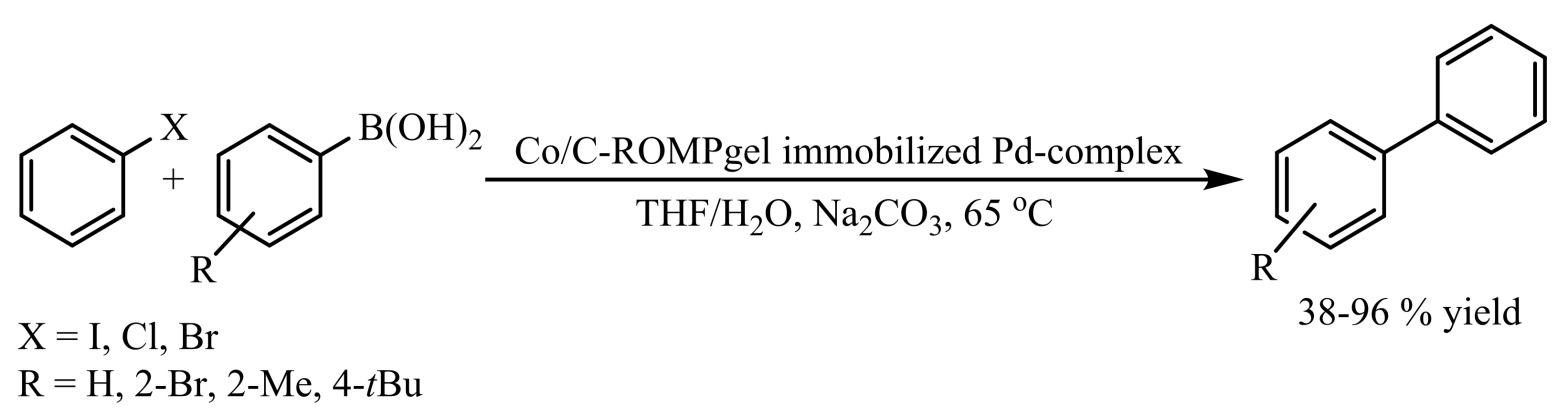
Suzuki‒Miyaura cross-coupling reactions catalyzed by recyclable Co/C-ROMPgel immobilized Pd complex [23].

The efficiency of the hybrid material was estimated from the Suzuki‒Miyaura cross-coupling reactions between iodobenzene, bromobenzene, and chlorobenzene with phenylboronic acid. Coupling reactions were conducted in THF/H_2_O at 65 °C by using Na_2_CO_3_ as the base (Scheme 6). Due to the ferromagnetic behavior of metal core NPs, an external magnetic field can be utilized to recycle the catalyst after each run, and the effectiveness of the catalyst is retained even after seven runs, which is due to high loading in ROMP technology.

**Scheme 7:**
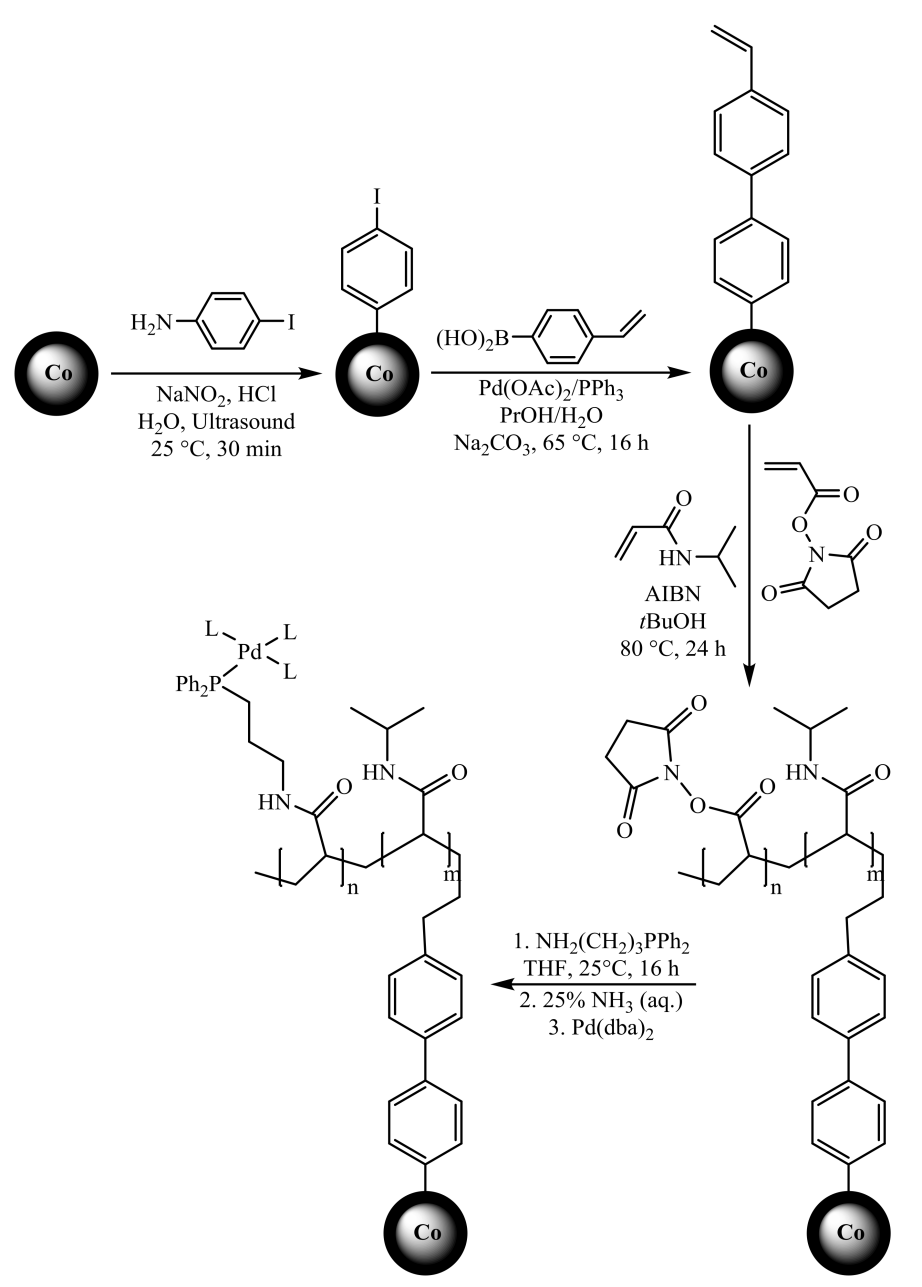
Preparation of recyclable C/Co@PNIPAM immobilized Pd complex. Reproduced with permission from [24].

In 2011, Stark and co-workers reported a self-separating phase switching catalyst system with grafting of palladium(II) onto amphiphilic *N*-isopropylacrylamide (PNIPAM) polymer-functionalized graphene-coated cobalt NPs (Scheme 7) [24].

**Scheme 8:**
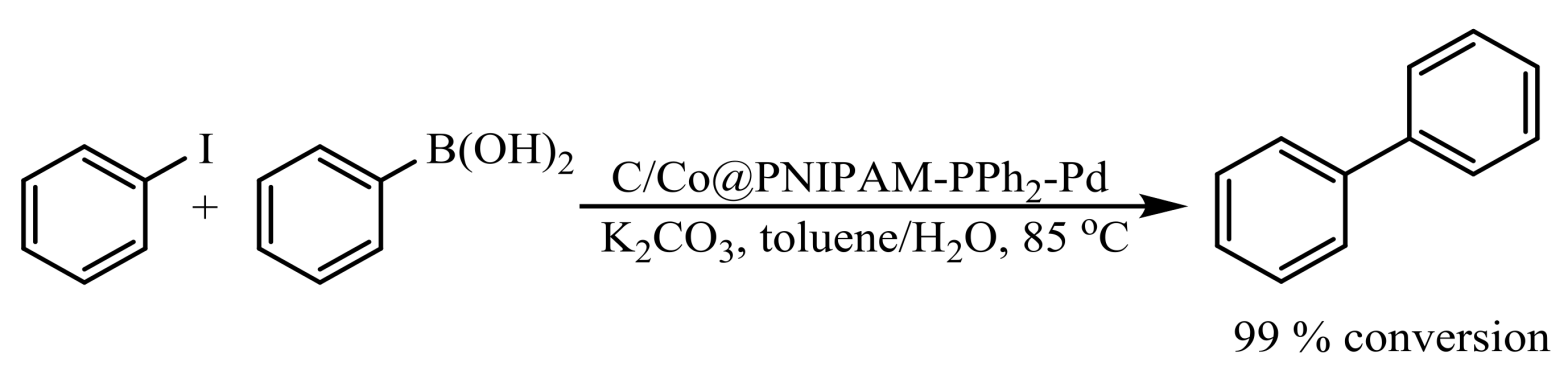
Suzuki‒Miyaura cross-coupling reactions catalyzed by recyclable C/Co@PNIPAM immobilized Pd complex. Reproduced with permission from [24].

The obtained catalyst was used successfully in Suzuki‒Miyaura cross-coupling reactions in the presence of K_2_CO_3_ as a base (Scheme 8). In this catalyst system, collapse of the polymer chains makes the material more hydrophobic; on the other hand, the cobalt core makes the catalyst magnetically separable. Due to the temperature-responsive nature of PNIPAM, adding toluene to a dispersed catalyst in water and increasing the temperature to 85 °C moved the particles into the organic phase and caused coupling of the aryl halide and boronic acid in a biphasic water/toluene mixture. Finally, after completion of the reaction and cooling the reaction mixture, defolding the polymeric chain results in transfer of the particles into the aqueous phase. The organic layer, including the product, is separated and the particles are removed from the aqueous phase by magnetic separation and can be reused 10 times or more.

**Scheme 9:**
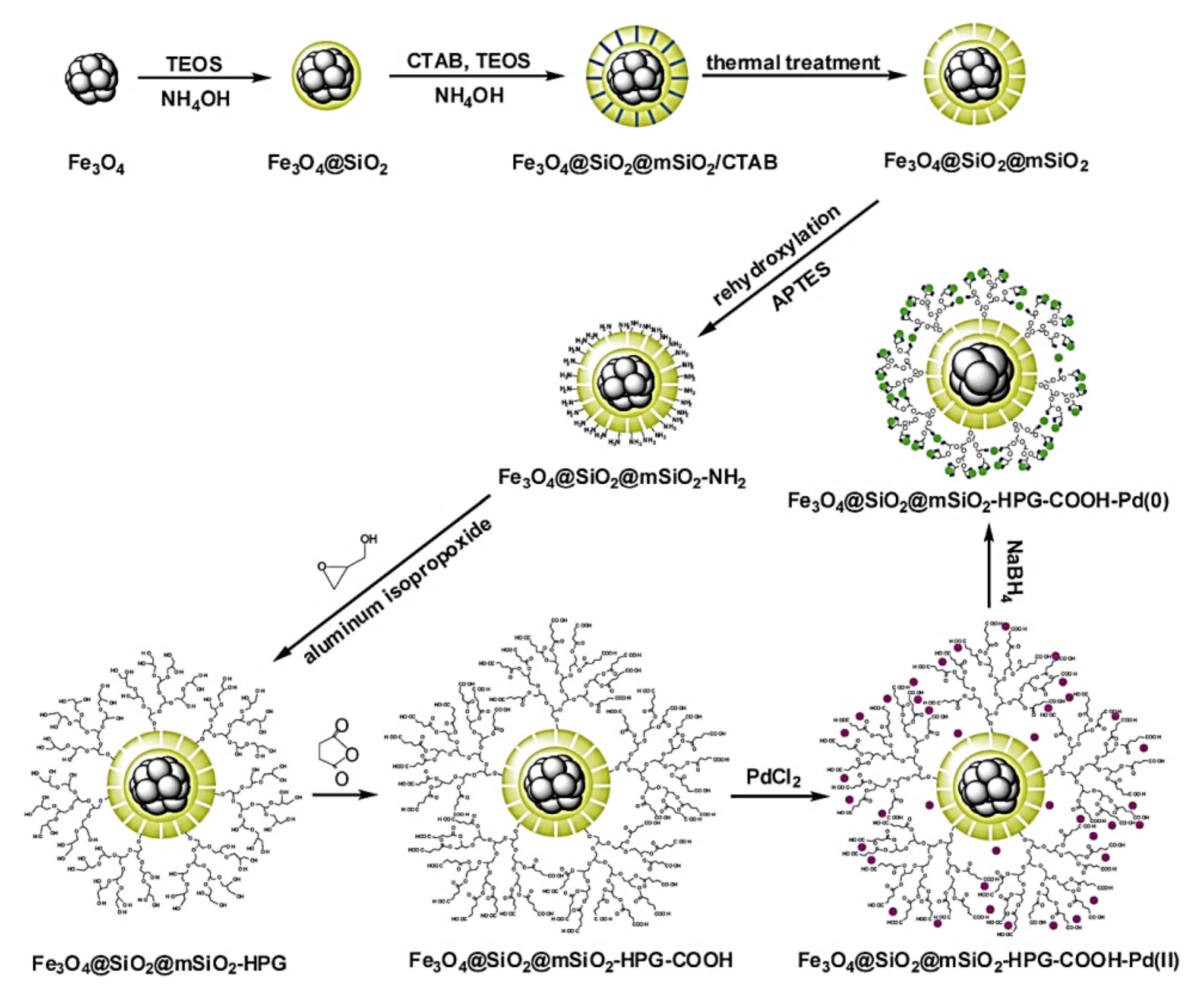
Preparation process of Fe_3_O_4_@SiO_2_@mSiO_2_-HPG-COOH-Pd(0). Reproduced with permission from [25].

A catalyst with palladium NPs supported on Fe_3_O_4_@SiO_2_@mSiO_2_-HPG-COOH microspheres was synthesized and evaluated in a Suzuki‒Miyaura cross-coupling reaction by Li and co-workers [25]. Grafting hyperbranch polyglycerol (HPG) onto the surface of Fe_3_O_4_@SiO_2_@mSiO_2_ microspheres, followed by a reaction of the mesopore wall of this material with isopropanol aluminum and epoxide opening with amino groups, gave Fe_3_O_4_@SiO_2_@mSiO_2_-HPG. Subsequently, treating this material with succinic anhydride resulted in the transformation of terminal hydroxyl groups of HPG into carboxyl groups. Finally, a Fe_3_O_4_@SiO_2_@mSiO_2_-HPG-COOH-Pd(0)-supported catalyst was obtained through complexation of Pd^2+^ ions and carboxyl groups (Scheme 9). 

**Scheme 10:**
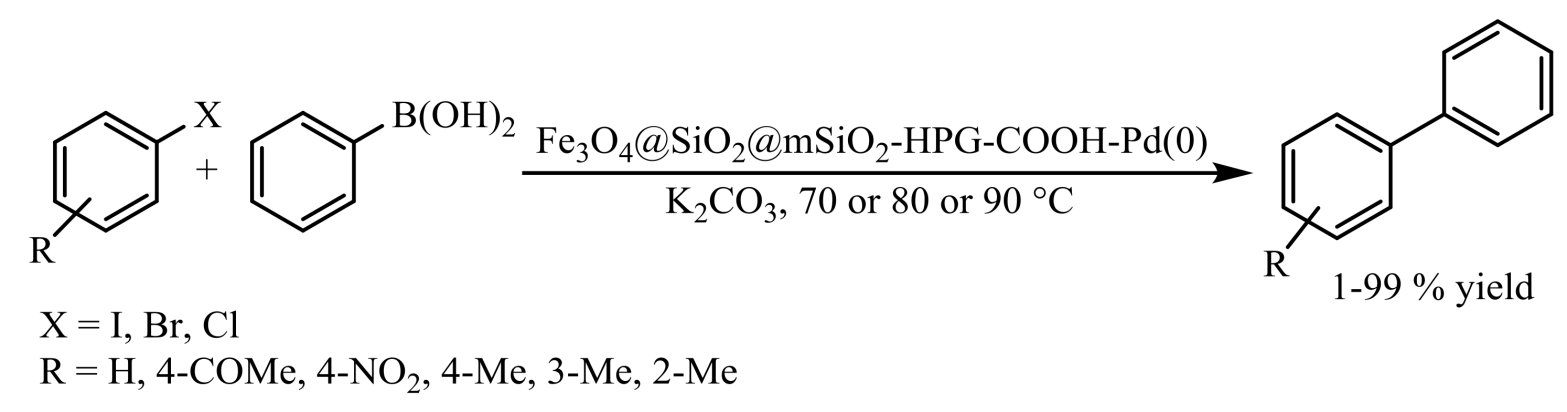
Suzuki‒Miyaura coupling reactions catalyzed by Fe_3_O_4_@SiO_2_@mSiO_2_-HPG-COOH-Pd(0) under different conditions [25].

The heterogenized catalyst showed remarkable catalytic activity in the Suzuki‒Miyaura cross-coupling reaction of aryl halides and phenylboronic acid (Scheme 10). The high catalytic performance and stability of this magnetic catalyst are due to numerous terminal carboxyl groups on the surface of the magnetic Fe_3_O_4_@SiO_2_@mSiO_2_ microspheres that can provide plenty of binding sites for Pd NPs. The supported catalyst can be reused at least eight times without evident loss of activity [25].

**Scheme 11:**
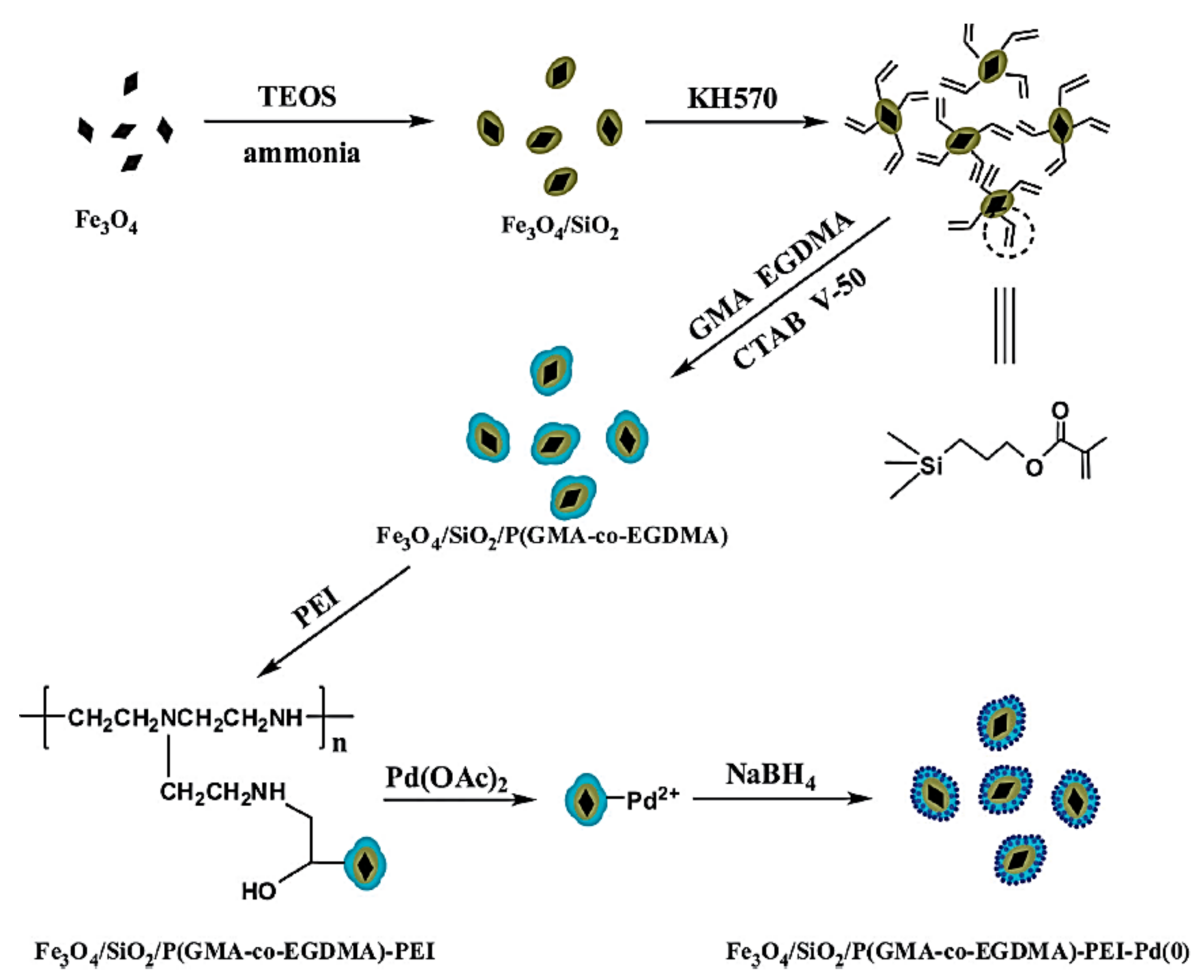
Preparation process of the Fe_3_O_4_/SiO_2_/P(GMA-co-EGDMA)-PEI-Pd(0) catalyst. Reproduced with permission from [26]. GMA: glycidyl methacrylate, EGDMA: ethyleneglycol dimethacrylate, CTAB: hexadecyltrimethylammonium bromide, V-50: 2,20-azobis-(2-methylpropionamide)dihydrochloride, PEI: polyethyleneimine.

Zhang et al. have prepared Pd NPs supported on branched/linear polyethylenimine-grafted magnetic Fe_3_O_4_/SiO_2_/P(GMA-co-EGDMA) composite [26]. The procedure and utilized steps for the preparation of catalyst are shown in Scheme 11. Hydrolyzing tetraethyl orthosilicate (TEOS) in the presence of Fe_3_O_4_ NPs, followed by surface-grafted copolymerization, gave a magnetic Fe_3_O_4_/SiO_2_/P(GMA-co-EGDMA) nanocomposite. Complexation between Pd^2+^ ions and imidogen groups or tertiary amine groups after grafting of the branched/linear polyethylenimine by a chemical method gave Fe_3_O_4_/SiO_2_/P(GMA-co-EGDMA)-PEI-Pd(0) catalyst.

**Scheme 12:**
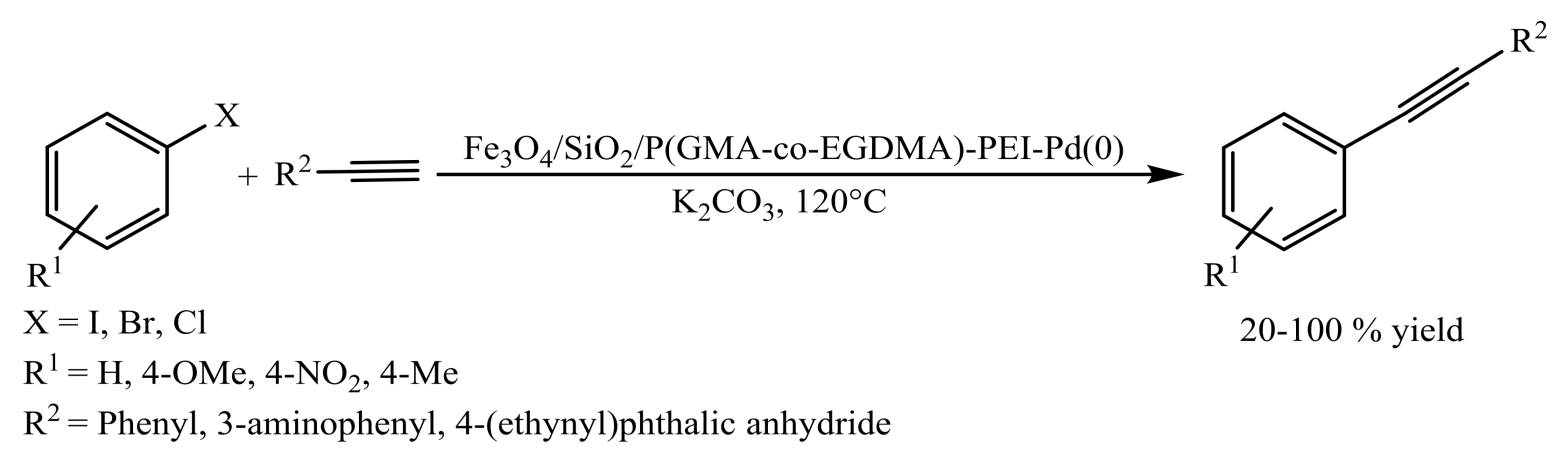
Sonogashira coupling reactions catalyzed by Fe_3_O_4_/SiO_2_/P(GMA-co-EGDMA)-PEI-Pd(0) [26].

The obtained material act as effective catalyst for coupling reactions of aryl halides and arylacetylenes (Sonogashira cross-coupling reaction) (Scheme 12). Gratifyingly, this catalyst can be reused at least eight times without significant loss of catalytic activity, which is conducive to its application. The yield of iodobenzene with phenylacetylene, 3-aminophenylacetylene, and 4-(ethynyl)phthalic anhydride is approximately 85%, 84%, and 100%, respectively. Recycling studies of the nanocatalyst were investigated for the Sonogashira cross-coupling reaction between iodobenzene and phenylacetylene, 3-aminophenylacetylene and 4-(ethynyl)phthalic anhydride, which indicated around 79%, 78%, and 95%, respectively, after eight times of reusing.

**Scheme 13:**
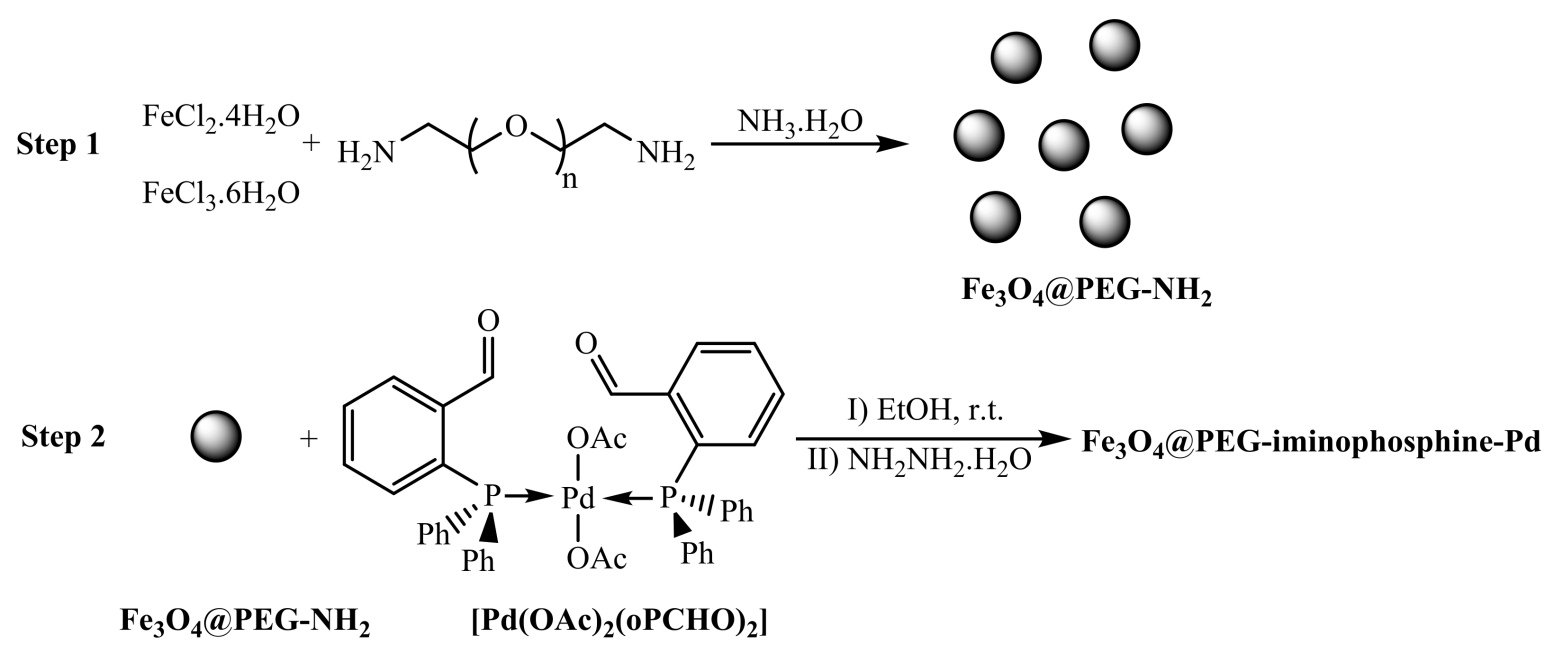
Preparation of Fe_3_O_4_@PEG-iminophosphine-supported nanopalladium catalyst [27].

**Figure 2:**
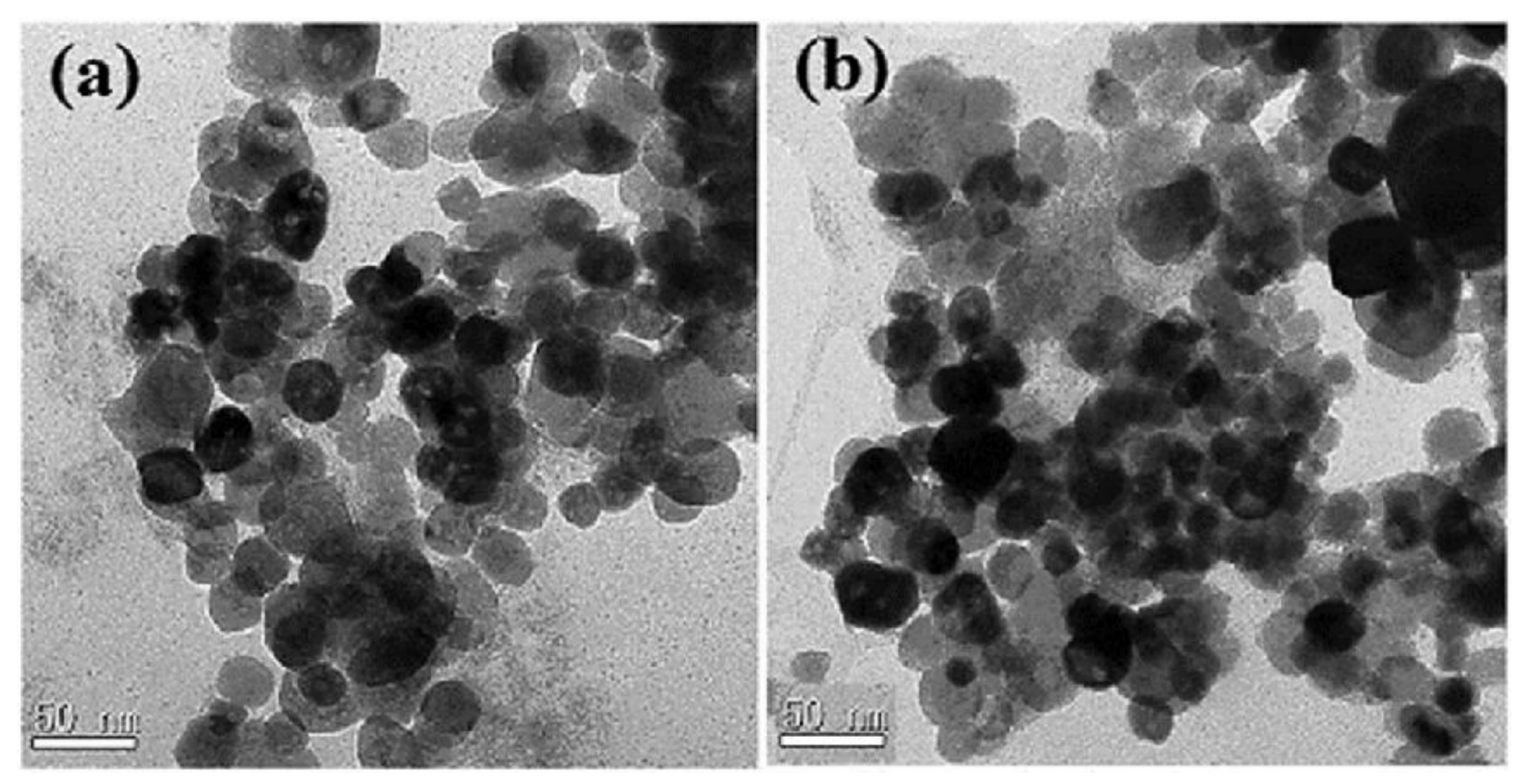
TEM images: (**a**) fresh Fe_3_O_4_@PEG-iminophosphine-supported palladium complex; (**b**) catalyst after being used five times. Reproduced with permission from [27].

Zhao and Lu designed a highly water-dispersible and magnetically separable palladium catalyst based on functionalized PEG-supported iminophosphine [27]. The magnetic NPs were prepared by reacting a palladium complex containing (diphenylphosphino) benzaldehyde as a ligand with amino-functionalized PEG-coated iron oxide NPs (Scheme 13). TEM images of the obtained catalyst showed mostly spherical particles with an average diameter of about 15–20 nm (Figure 2a).

**Scheme 14:**
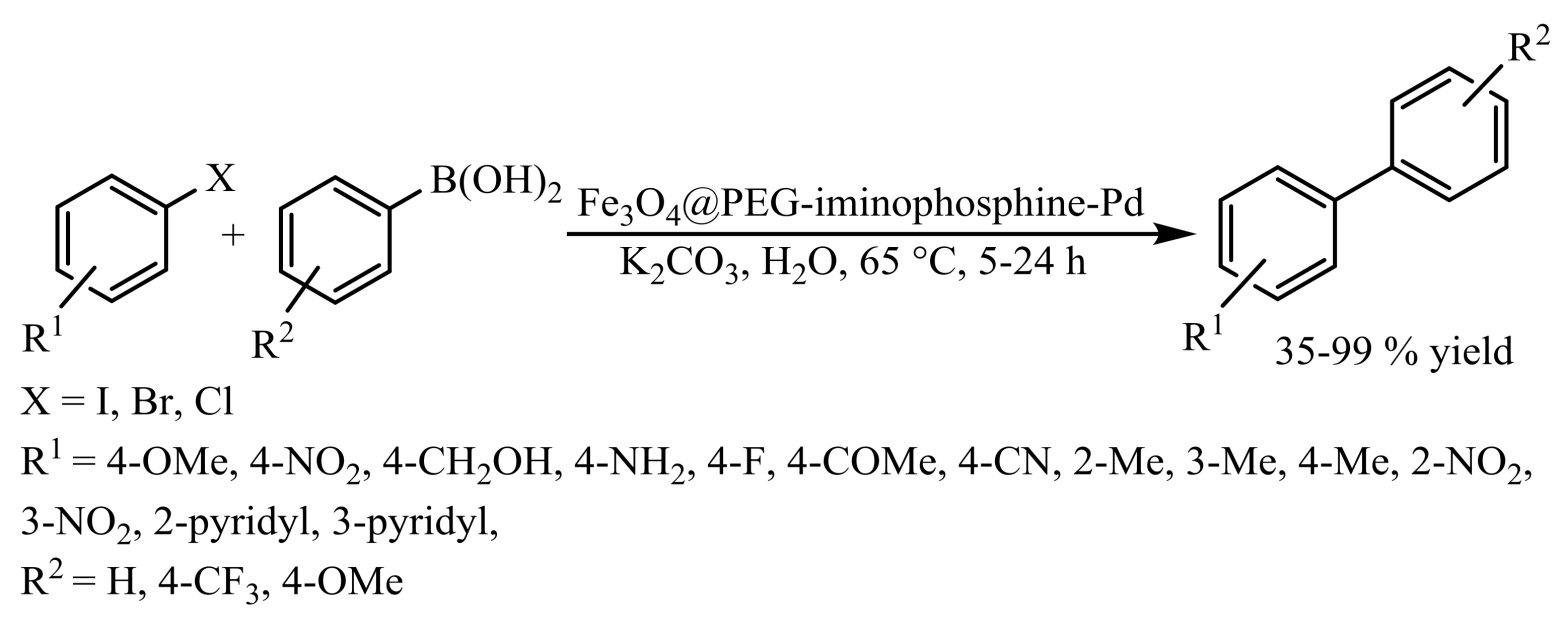
Suzuki‒Miyaura reaction of aryl halides with arylboronic acids catalyzed by Fe_3_O_4_@PEG-iminophosphine‒Pd complex [27].

Suzuki‒Miyaura reaction of aryl halides with arylboronic acids based on functionalized PEG-supported iminophosphine in neat water were investigated. The heterogeneous palladium catalyst presented good activity in the transportation and all of the coupling products were obtained in good to excellent yields (Scheme 14). Furthermore, a magnetically separable catalyst can be recovered from the reaction mixture using an external magnet and reused for five consecutive runs without a significant decrease in its activity. The TEM image of the recovered catalyst after the fifth reaction run also indicated that the Fe_3_O_4_@PEG-iminophosphine-supported palladium catalyst maintained its core-shell structure after five consecutive Suzuki‒Miyaura reactions (Figure 2b) [27].

**Scheme 15:**
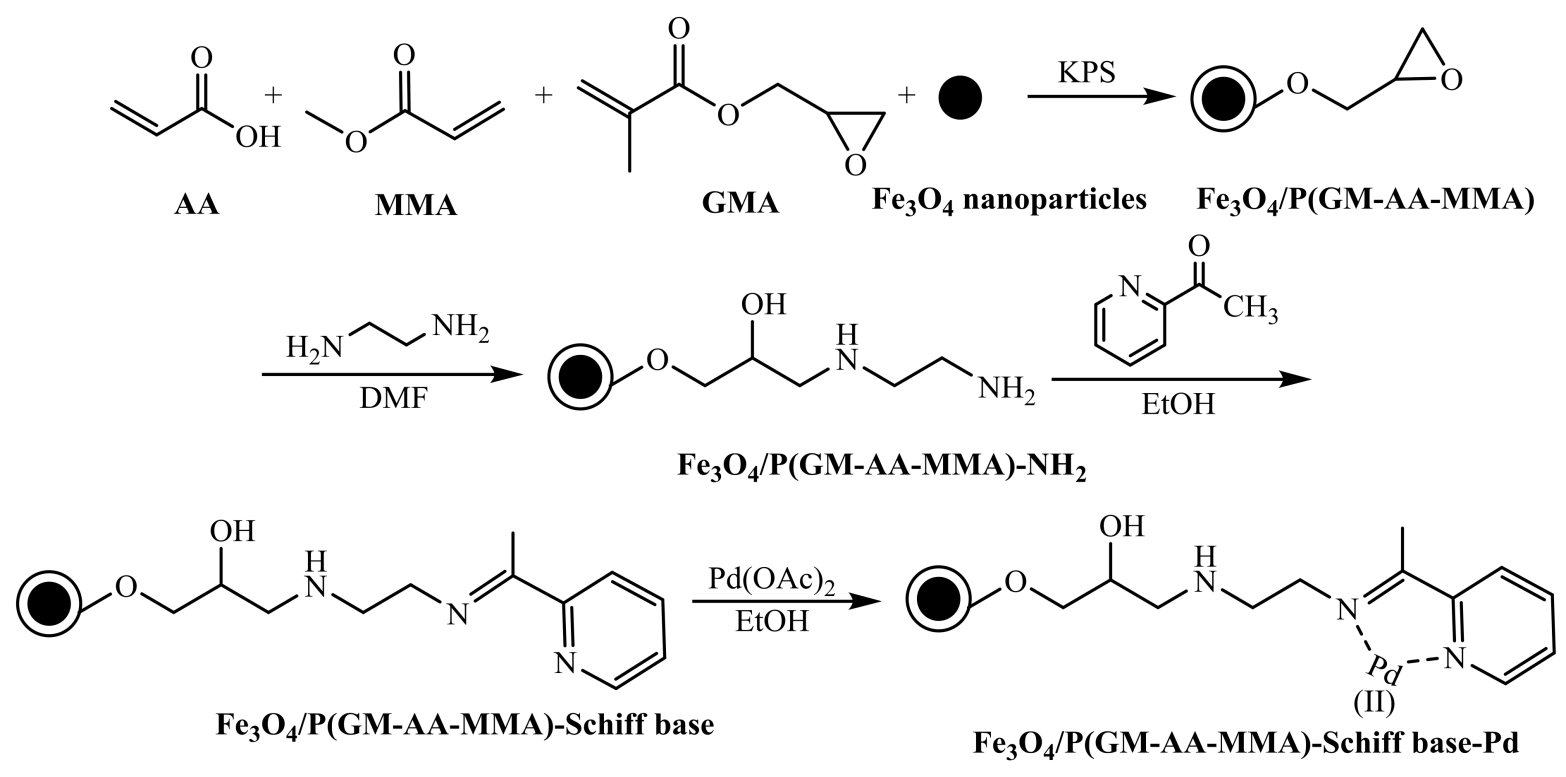
Synthesis of Fe_3_O_4_/P(GMA-AA-MMA)-Schiff base Pd [28].

**Figure 3:**
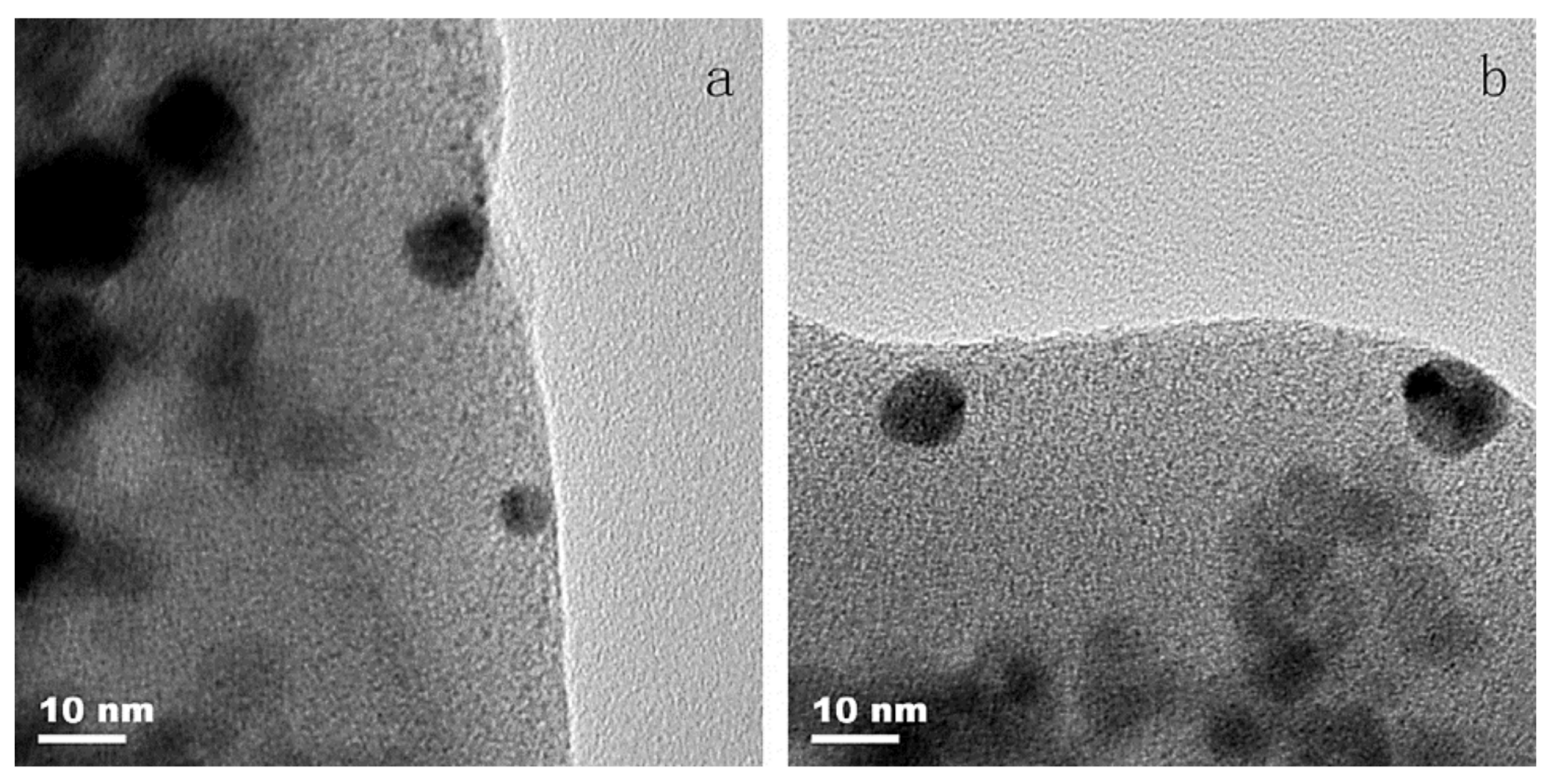
HRTEM images of Fe_3_O_4_/P(GMA-AA-MMA)-Schiff base-Pd before (**a**) and after (**b**) Suzuki‒Miyaura reaction. Reproduced with permission from [28].

Yuan and co-workers reported a soap-free emulsion polymerization technique for the formation of superparamagnetic polymer composite microspheres as the support for immobilization of Schiff base palladium catalyst for Suzuki‒Miyaura coupling reactions (Scheme 15) [28]. HRTEM TEM of Fe_3_O_4_/P(GMA-AA-MMA)-Schiff base Pd showed Pd NPs with an average size of about 5–10 nm on the surface of the support materials (Figure 3).

**Scheme 16:**
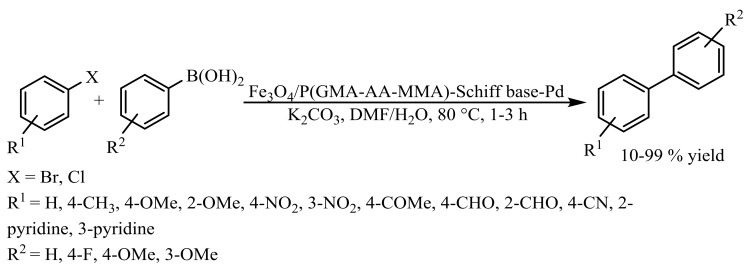
Suzuki‒Miyaura reactions between aryl halides and aryl boronic acids catalyzed by Fe_3_O_4_/P(GMA-AA-MMA)-Schiff base Pd [28].

This magnetic catalyst demonstrated excellent reactivity in the Suzuki‒Miyaura coupling reactions of aryl halides bearing both electron-withdrawing and electron-donating groups with phenylboronic acids (Scheme 16) and could be reused at least seven times without any loss of catalytic activity or Pd leaching. As shown in Figure 3, the HRTEM image of the catalyst after seven times of its frequent using revealed no change in Pd size and also ICP-AES results showed insignificant Pd leaching of the supported magnetic catalyst.

**Scheme 17:**
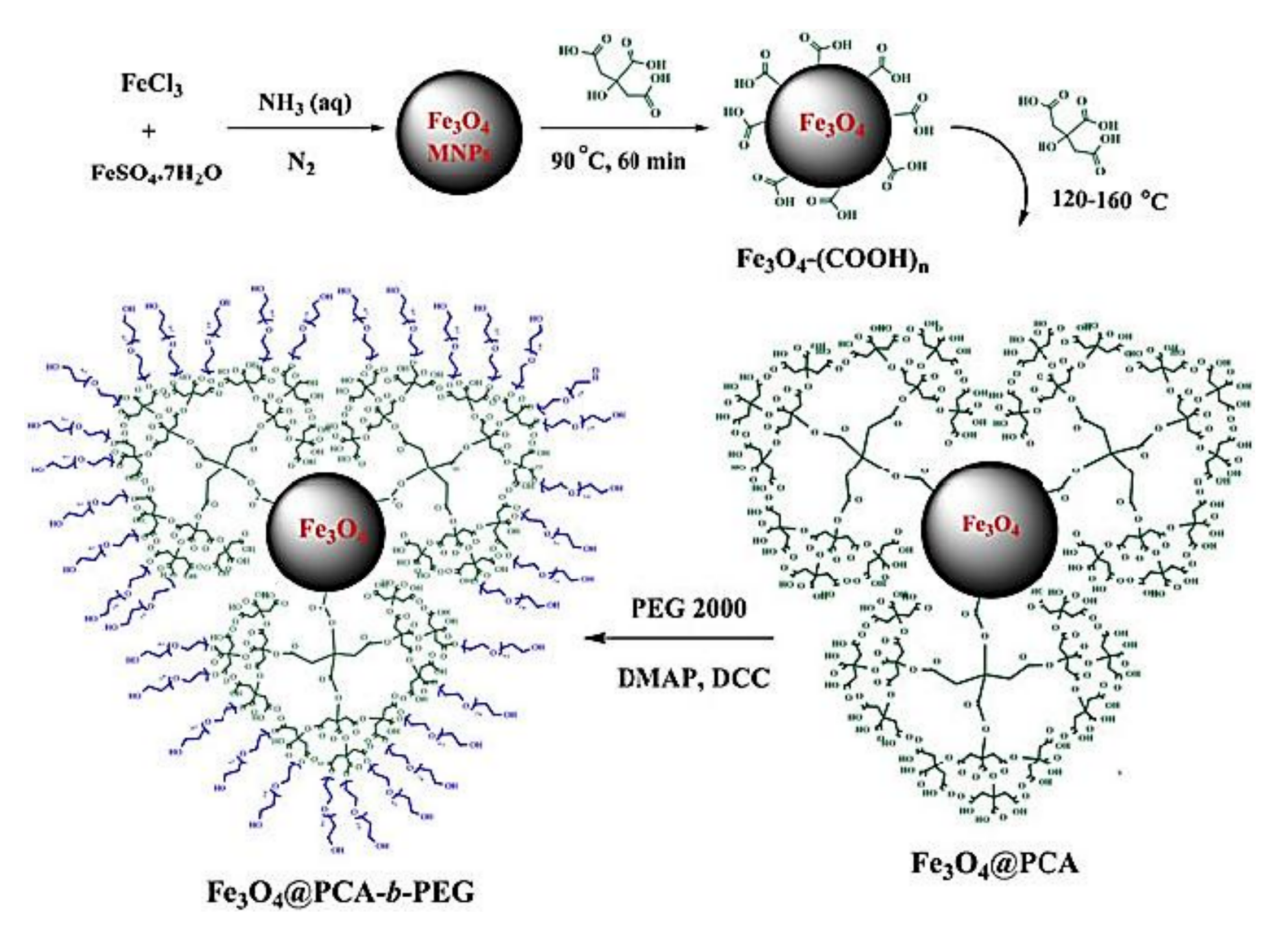
Synthetic route employed for preparation of Fe_3_O_4_@PCA-*b*-PEG magnetic NPs. Reproduced with permission from [29].

**Figure 4:**
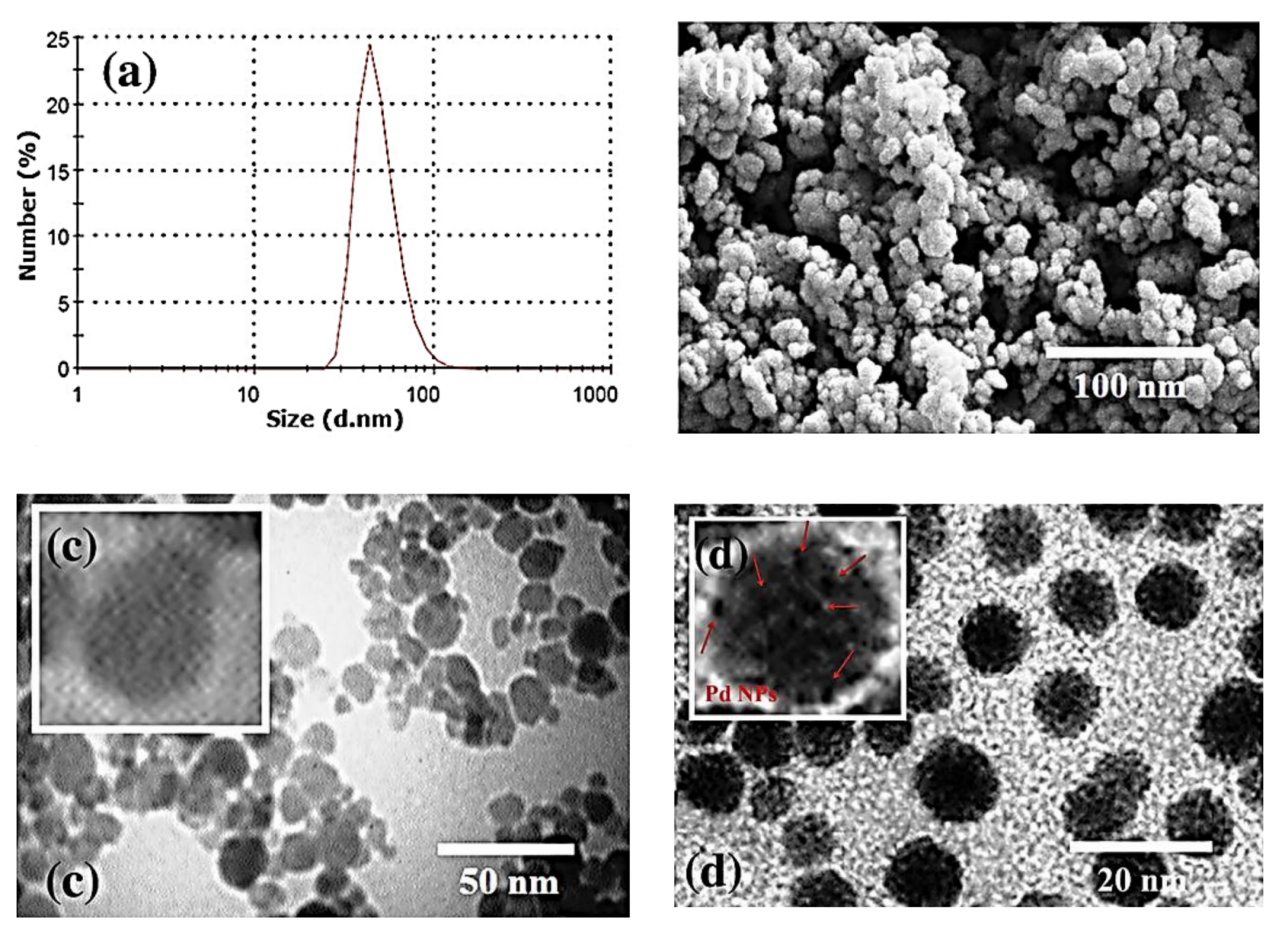
(**a**) DLS plot of Fe_3_O_4_@PCA/Pd(0)-*b*-PEG in water. (**b**) SEM image of Fe_3_O_4_@PCA-*b*-PEG. TEM images of (**c**) Fe_3_O_4_@PCA-*b*-PEG and (**d**) Fe_3_O_4_@PCA/Pd(0)-*b*-PEG at different magnifications. Reproduced with permission from [29].

Tabatabaei Rezaei and co-workers reported a novel magnetically retrievable palladium catalyst for the C‒C cross-coupling of aryl halides with olefin compounds. The catalyst was prepared by immobilizing palladium NPs on amphiphilic and hyperbranched polymer-functionalized magnetic NPs. In this catalyst, hyperbranched poly (ethylene glycol)-*block* poly (citric acid)-functionalized Fe_3_O_4_ magnetic NPs (Fe_3_O_4_@PCA-*b*-PEG) with the presence of a large number of carboxyl groups as anchoring sites are capable to effectively immobilize and stabilize Pd NPs (Scheme 17) [29]. The TEM image indicates the dispersion of Pd NPs into the hyperbranched polymers on the surface of Fe_3_O_4_ and also size distribution diagram indicates a uniform distribution of the particles with an average diameter of about 49.5 nm (Figure 4).

**Scheme 18:**
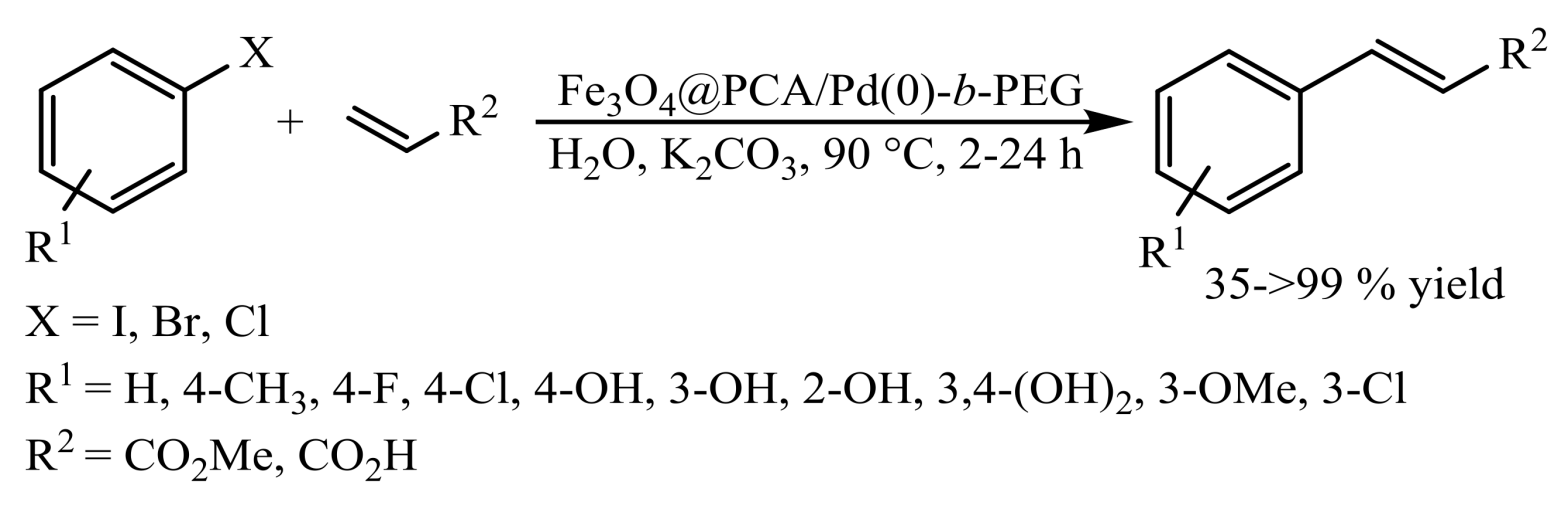
Heck reaction of acrylic acid and methyl acrylate with various aryl halides in the presence of Fe_3_O_4_@PCA-*b*-PEG magnetic NPs [29].

The heterogenized catalyst was used in the palladium-catalyzed Heck coupling reaction of aryl halides with olefin compounds in the presence of water as a green solvent and K_2_CO_3_ as base (Scheme 18). In addition, the catalyst could be simply separated from the reaction mixture by applying an external magnetic field and reused for more than 10 consecutive cycles without much loss in its activity.

### 2.4. Ligand-Functionalized Magnetic Nanoparticles

**Scheme 19:**
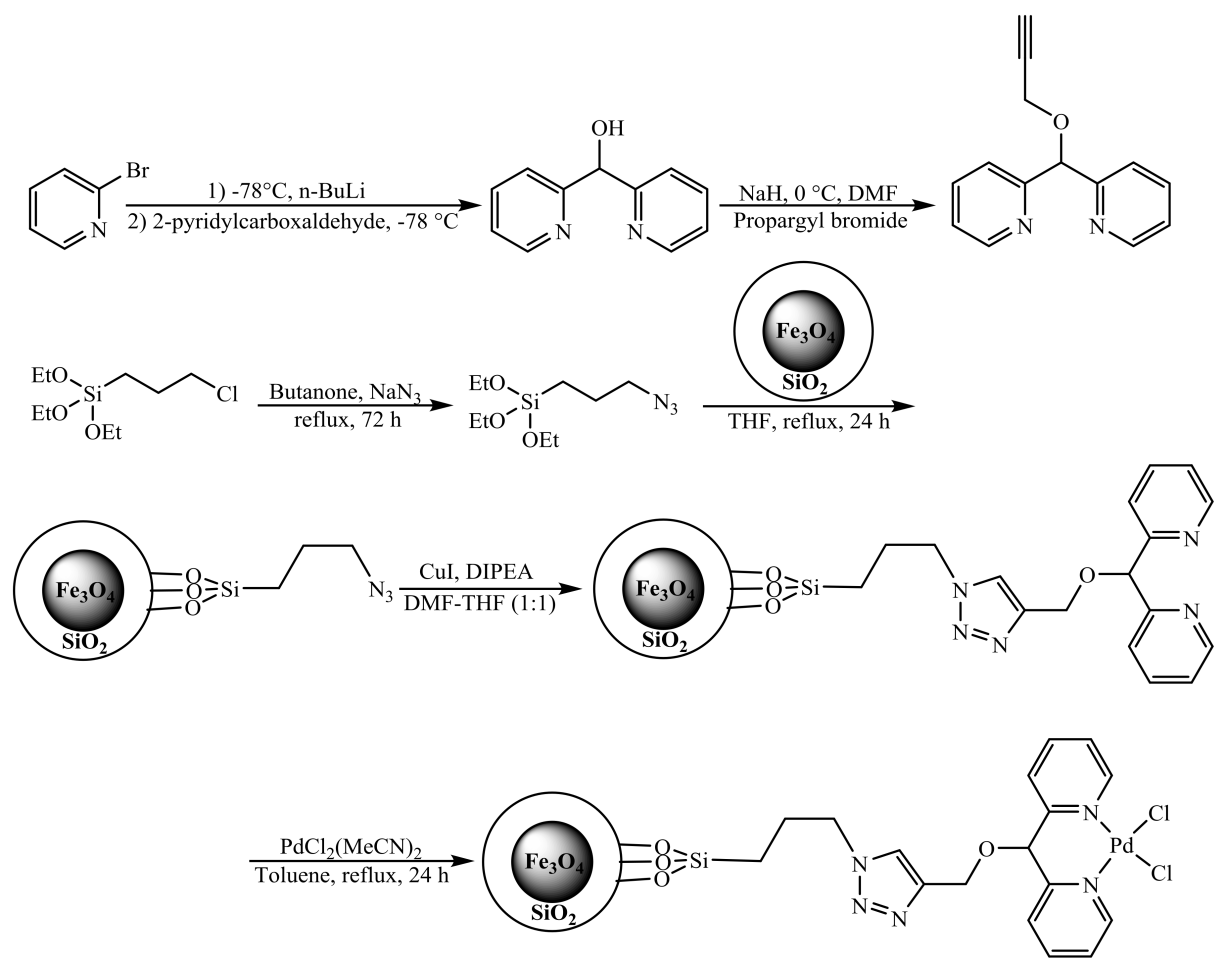
Preparation of MNP-supported di(2-pyridyl)methanol‒Pd complex. Reproduced with permission from [30].

Generally, a popular approach for the effective and stable immobilization of a metal catalyst on the surfaces of magnetic NPs is using a suitable organic ligand as a linker between an inorganic support and a metal catalyst core. Therefore, nature of magnetic NPs combines with a highly stable metal complex to arrive at recyclable catalysts. In this regard, Gao and co-workers, by click chemistry, immobilized dipyridyl palladium complex onto magnetite core/shell particles and prepared a catalyst for the Suzuki‒Miyaura cross-coupling reaction [30]. For preparation of catalyst, silica-capped magnetite nanoparticles functionalized by 3-azido-1-triethoxysilylpropane followed by copper-catalyzed triazole formation using an alkyne-functionalized dipyridyl precursor obtained covalently fixed ligand for coordination with palladium(II) (Scheme 19).

**Scheme 20:**
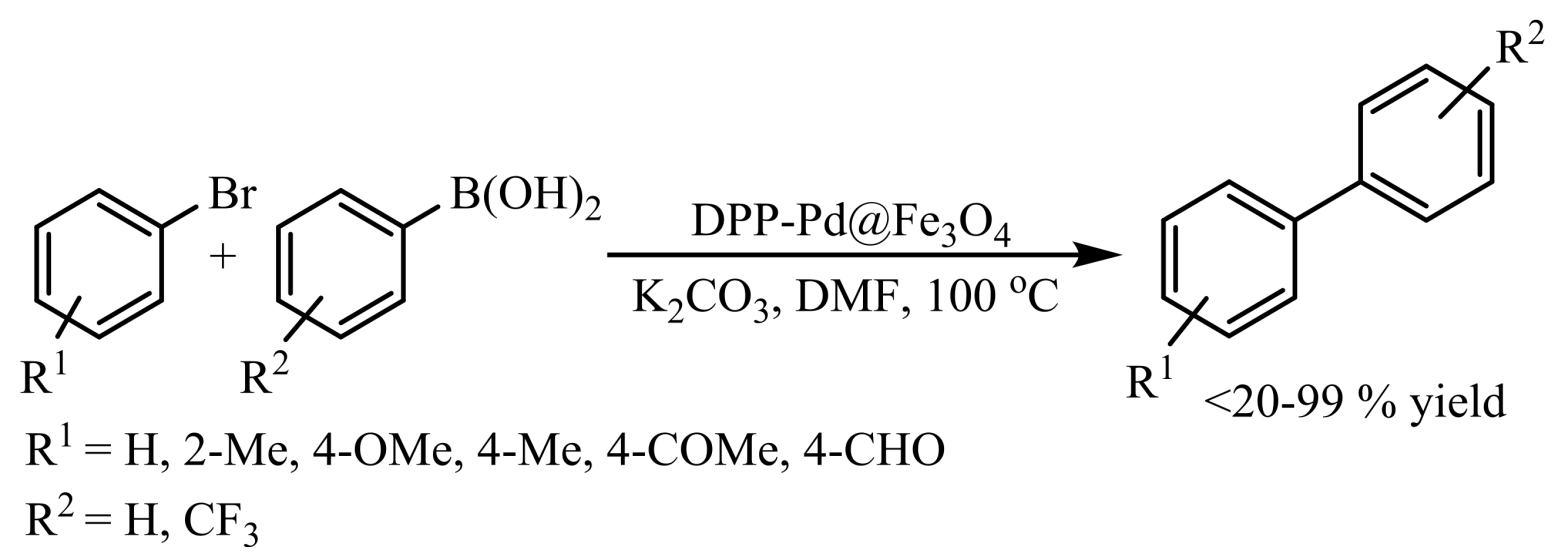
DPP-Pd@Fe_3_O_4_ catalyst in Suzuki‒Miyaura coupling of aryl halides and aryl boronic acids [30].

The activity of resulting DPP-Pd@Fe_3_O_4_ catalyst was evaluated in the Suzuki‒Miyaura cross-coupling of different aryl halides with arylboronic acids by using K_2_CO_3_ as the base in DMF, which showed very good to excellent yields (Scheme 20). Moreover, the recyclable catalyst could be reused for five consecutive runs without a significant loss in activity. 

**Scheme 21:**
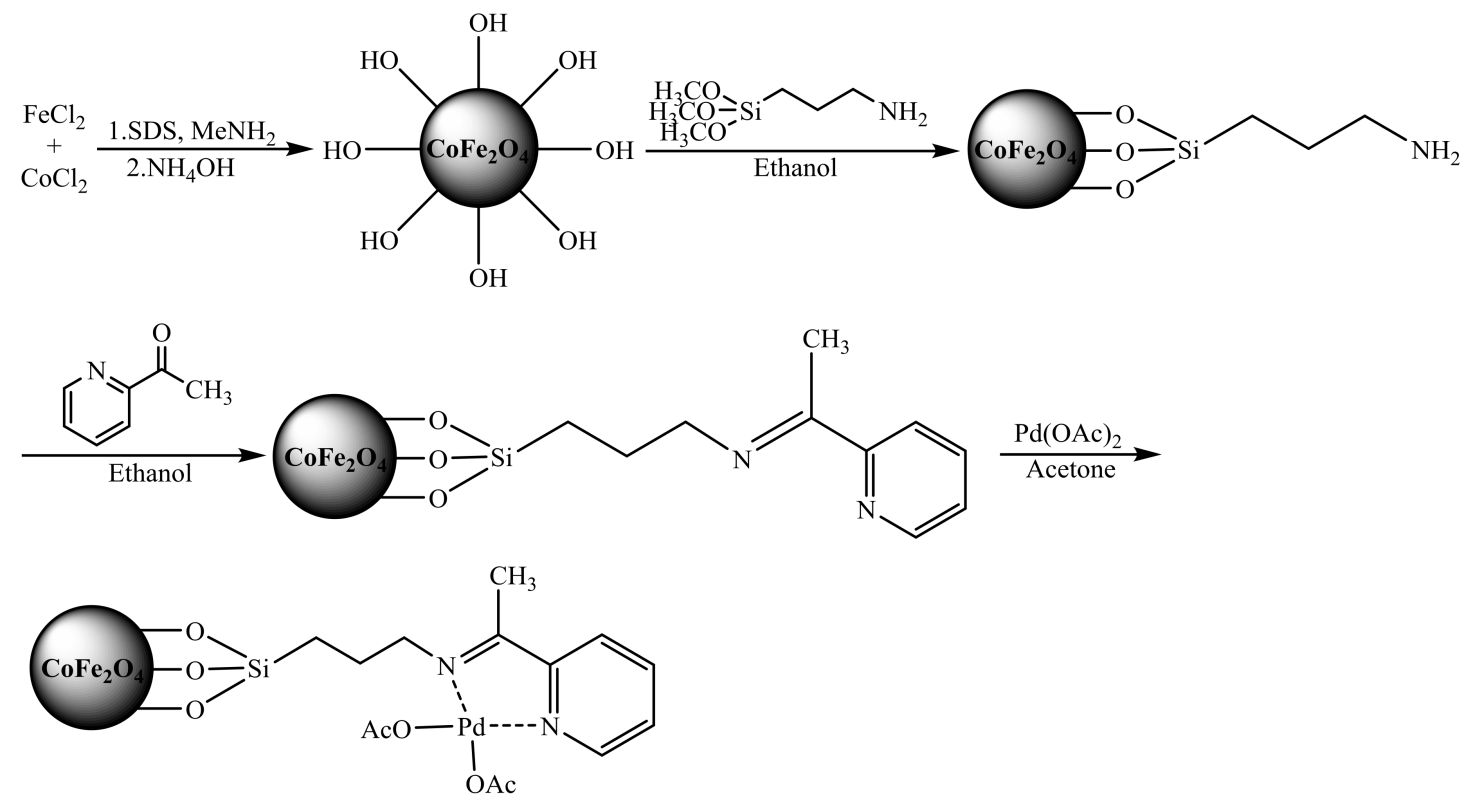
Synthesis of the palladium catalyst immobilized on superparamagnetic NPs [31].

Phan and co-workers reported palladium complex immobilized on pyridinylimine-functionalized cobalt ferrite NPs and investigated its catalytic activity in Sonogashira reaction of aryl halides and phenylacetylene [31]. Superparamagnetic NPs were obtained through microemulsion method; functionalization of this material with Schiff base groups gave immobilized bidentate ligands. Adding palladium acetate result in complexation of palladium with immobilized ligand and affording the superparamagnetic NPs-supported phosphine-free palladium catalyst (Scheme 21).

**Scheme 22:**
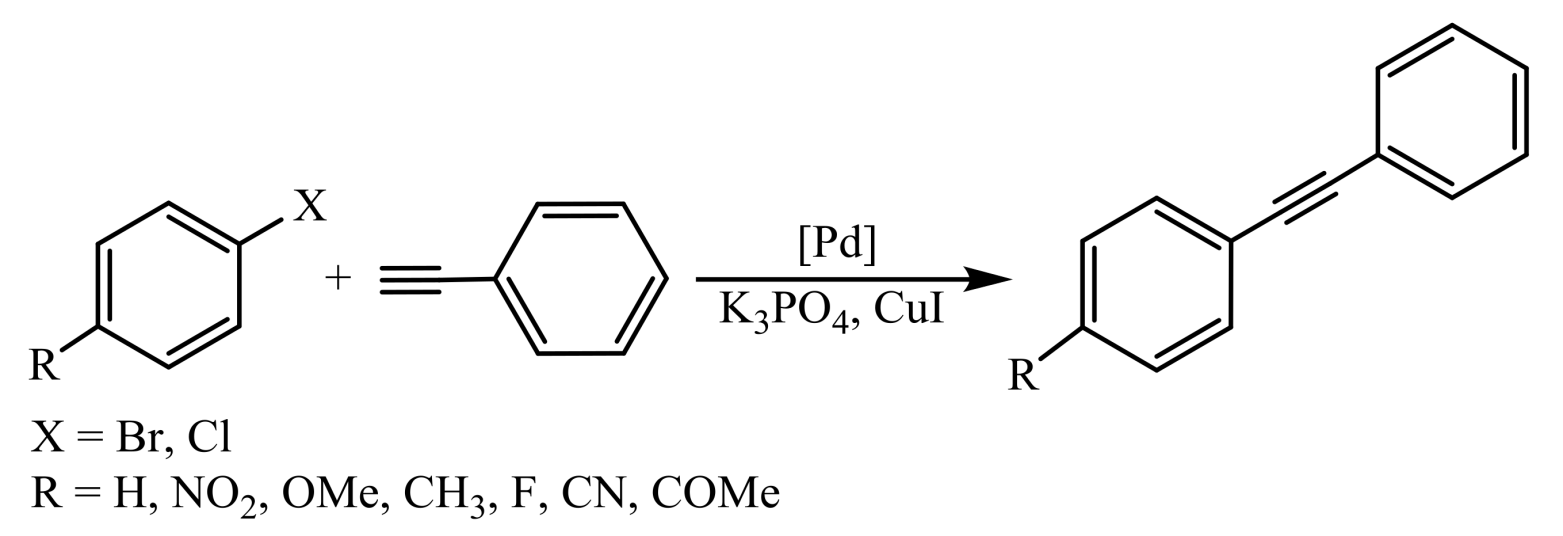
The Sonogashira reaction of aryl halides and phenylacetylene using the palladium complex catalyst immobilized on the superparamagnetic NPs [31].

The Sonogashira reaction takes place in the presence of K_3_PO_4_ as a base without adding phosphine ligands and a corresponding diphenylacetylene product; in the case of electron-withdrawing groups on the benzene ring, it was obtained in favored yields, whereas electron-donating groups slowed down the cross-coupling process (Scheme 22). Superparamagnetic NPs could be recovered and reused more than 10 times with no significant decrease in their catalytic activity.

**Scheme 23:**
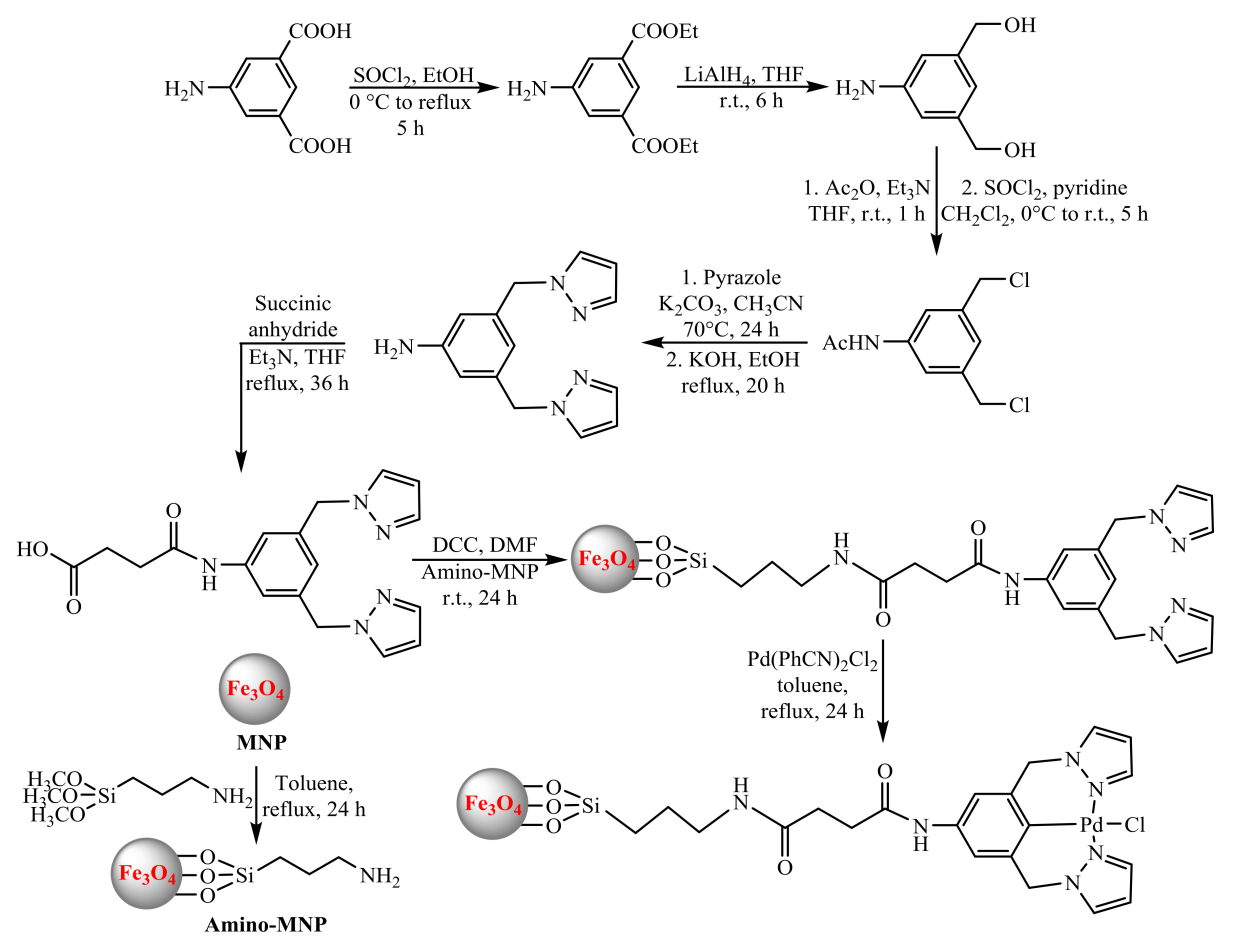
Synthesis of magnetic nanoparticle (MNP)-grafted NCN pincer Pd catalyst [32].

**Figure 5:**
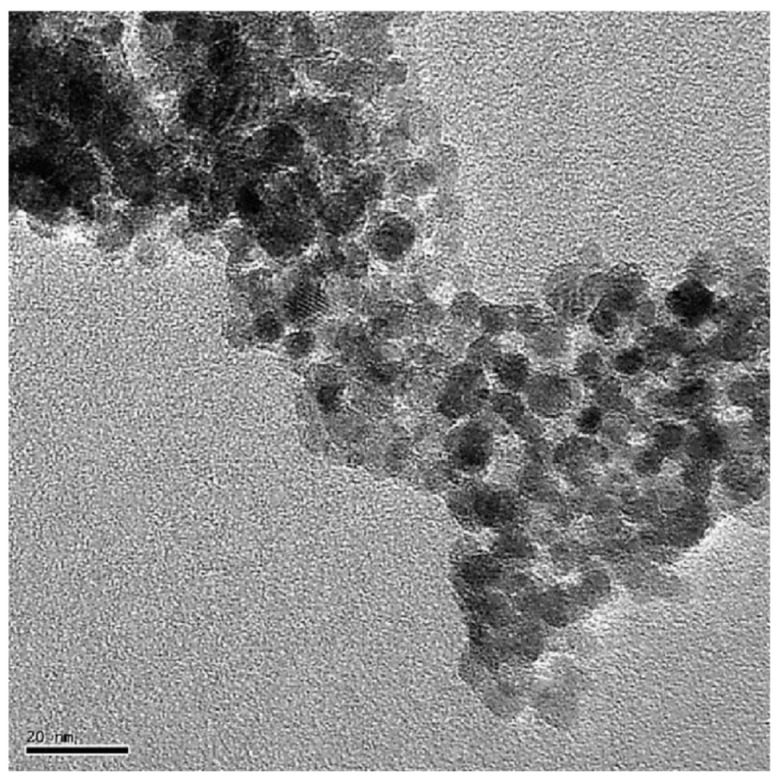
Transmission electron microscopy (TEM) image of magnetic nanoparticle (MNP)-grafted NCN pincer Pd catalyst. Reproduced with permission from [32].

Zheng and co-workers reported the preparation of a palladium pincer complex immobilized on magnetic NPs and investigated its catalytic activity in the reductive homocoupling of aryl halides [32]. The Fe_3_O_4_ NPs were synthesized through thermal decomposition of Fe(Acac)_3_ in triethylene glycol, modification of these materials with 3-aminopropyltrimethoxysilane followed by grafting 4-{3,5-bis[(1*H*-pyrazol-1-yl)methyl]phenylamino}-4-oxobutanoic acid onto the surface of amine-functionalized Fe_3_O_4_ magnetic NPs resulted NCN pincer ligand for the formation of complex with palladium (Scheme 23). The formed MNPs were composed of relatively uniform particles with an average size of about 6 ± 1 nm (Figure 5). 

**Scheme 24:**
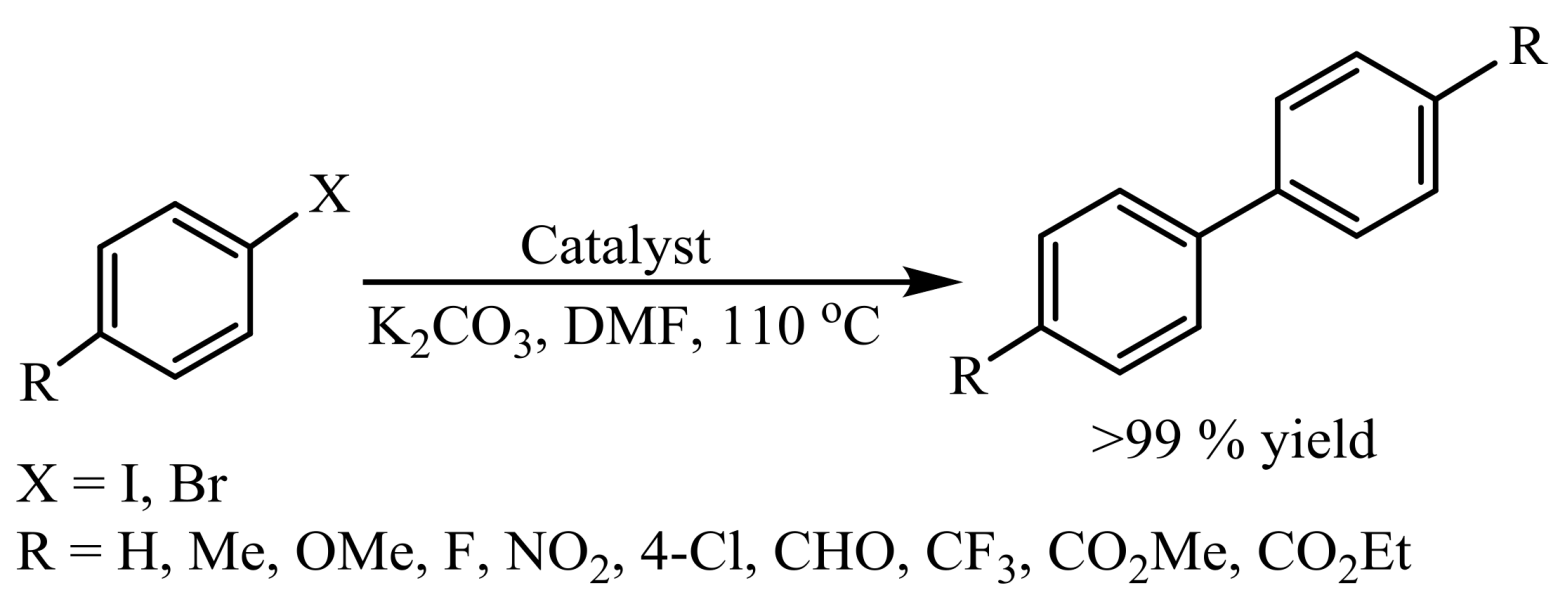
Homocoupling reaction of aryl halides in the present of magnetic nanoparticle (MNP)-grafted NCN pincer Pd catalyst [32].

This catalyst with magnetic nanoparticle support acts as the reducing agent and catalyzes the reductive homocoupling of various aryl halides in the absence of additional reducing agents. A homocoupling reaction in DMF at 110 °C by using K_2_CO_3_ as the base demonstrated symmetrical biaryls in quantitative yields (Scheme 24). Furthermore, the catalyst can be recycled using a magnetic bar and reused in five consecutive runs. With the application of the catalyst for more than five runes, homocoupling yield was decreased, indicating the diminutive reducing capability of the MNPs. To confirm the possibility of the Fe_3_O_4_ magnetic core acting as a reducing agent, the XRD pattern of the recovered catalyst was analyzed. It indicated oxidation of Fe_3_O_4_ to Fe_2_O_3_ after five cycles and proved the synergetic role of Fe_3_O_4_ as a reducing agent in this process.

**Scheme 25:**
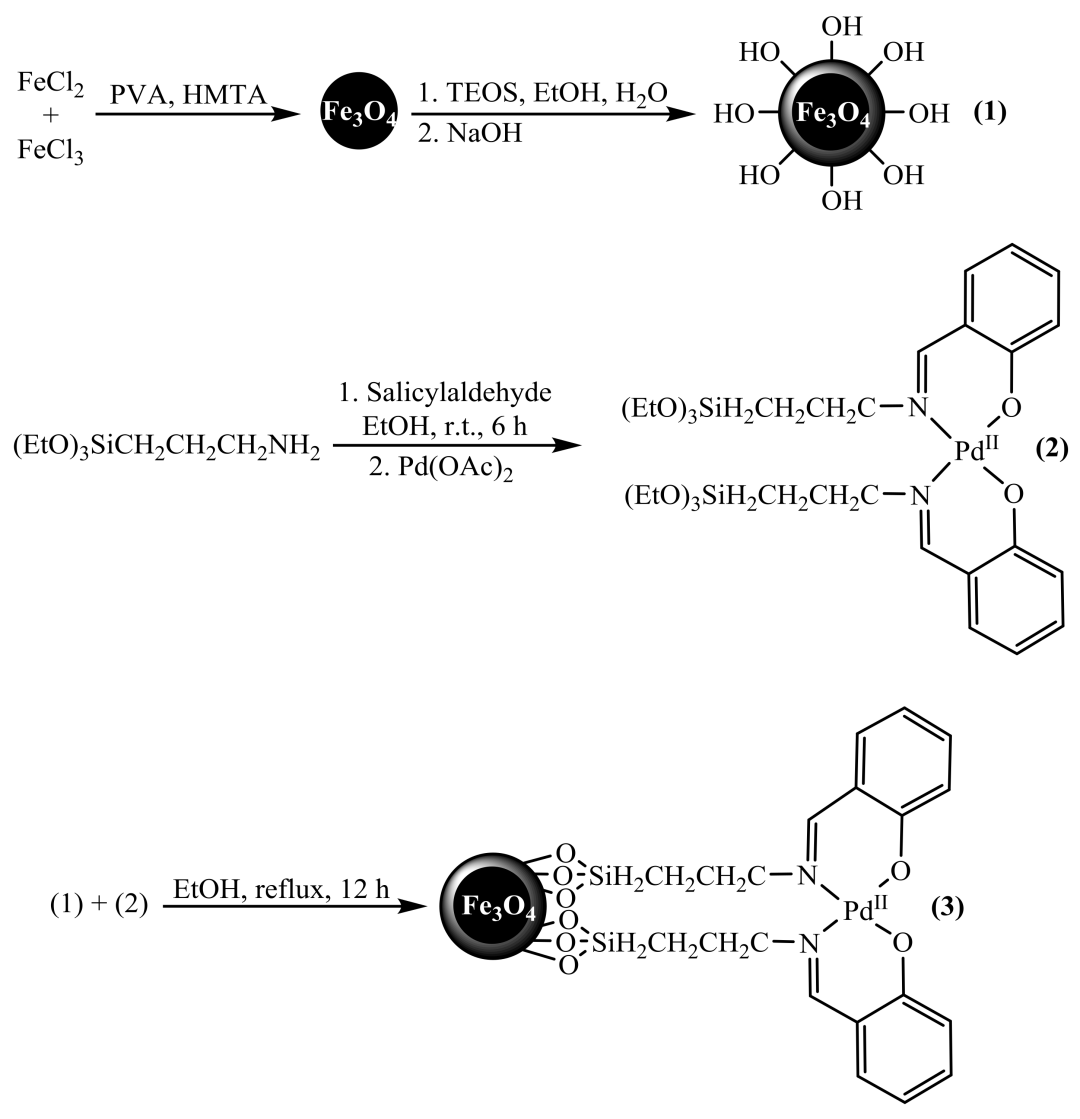
Preparation process of Schiff base complex of Pd(II) functionalized Fe_3_O_4_@SiO_2_ NPs. Reproduced with permission from [33].

Esmaeilpour and co-workers prepared a Schiff base complex of Pd(II) immobilized on super-paramagnetic Fe_3_O_4_ NPs. The Schiff base complex was prepared by the condensation of 3-aminopropyl (triethoxy) silane with salicylaldehyde in ethanol followed by treatment with palladium acetates. Refluxing the Schiff base complex and silica-coated Fe_3_O_4_ in ethanol yielded the Schiff base complex of Pd(II) functionalized Fe_3_O_4_@SiO_2_ NPs (Scheme 25) [33].

**Scheme 26:**
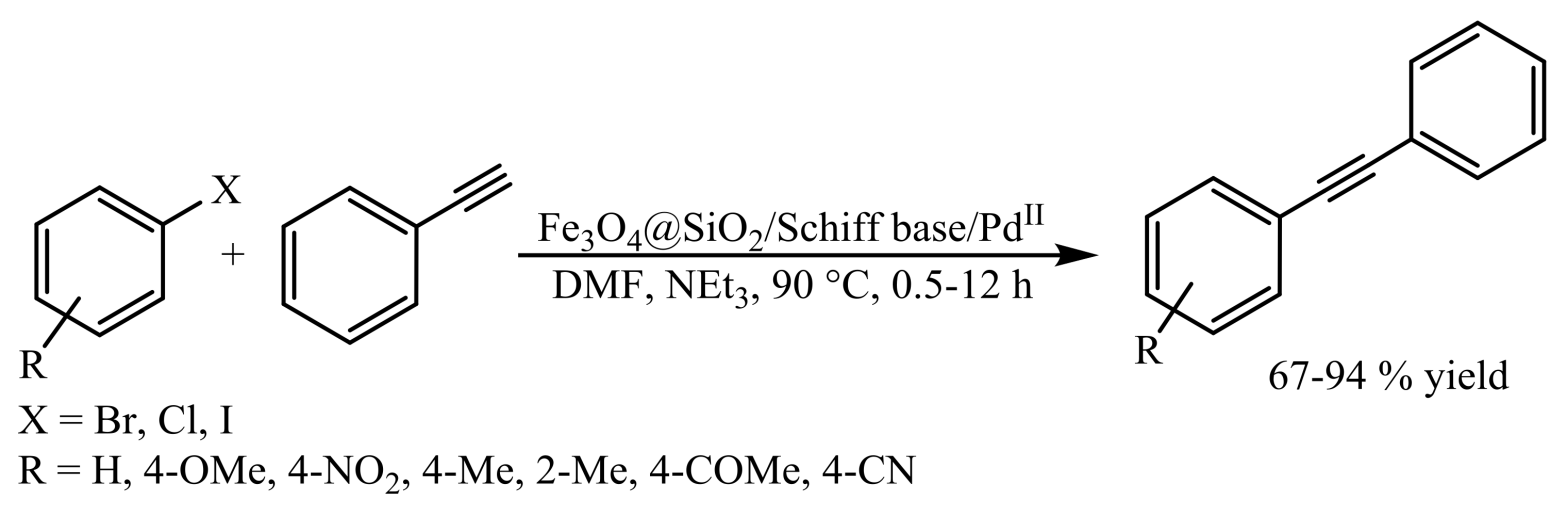
Sonogashira‒Hagihara coupling reaction in the presence of Fe_3_O_4_@SiO_2_/Schiff base/Pd^II^ [33].

The heterogenized catalyst was used for copper- and phosphine-free Sonogashira reaction of various substituted aryl halides and phenylacetylene. High yields of diphenyl acetylene as the principal product were obtained in the presence of NEt_3_ as a base in DMF (Scheme 26). Simplicity of operation, excellent yields, short reaction times, heterogeneous nature, easy magnetic work-up, and recyclability are some notable advantages of this catalyst. Recycling studies showed that the catalyst can be reused at least six times without any noticeable loss of catalytic activity.

**Scheme 27:**
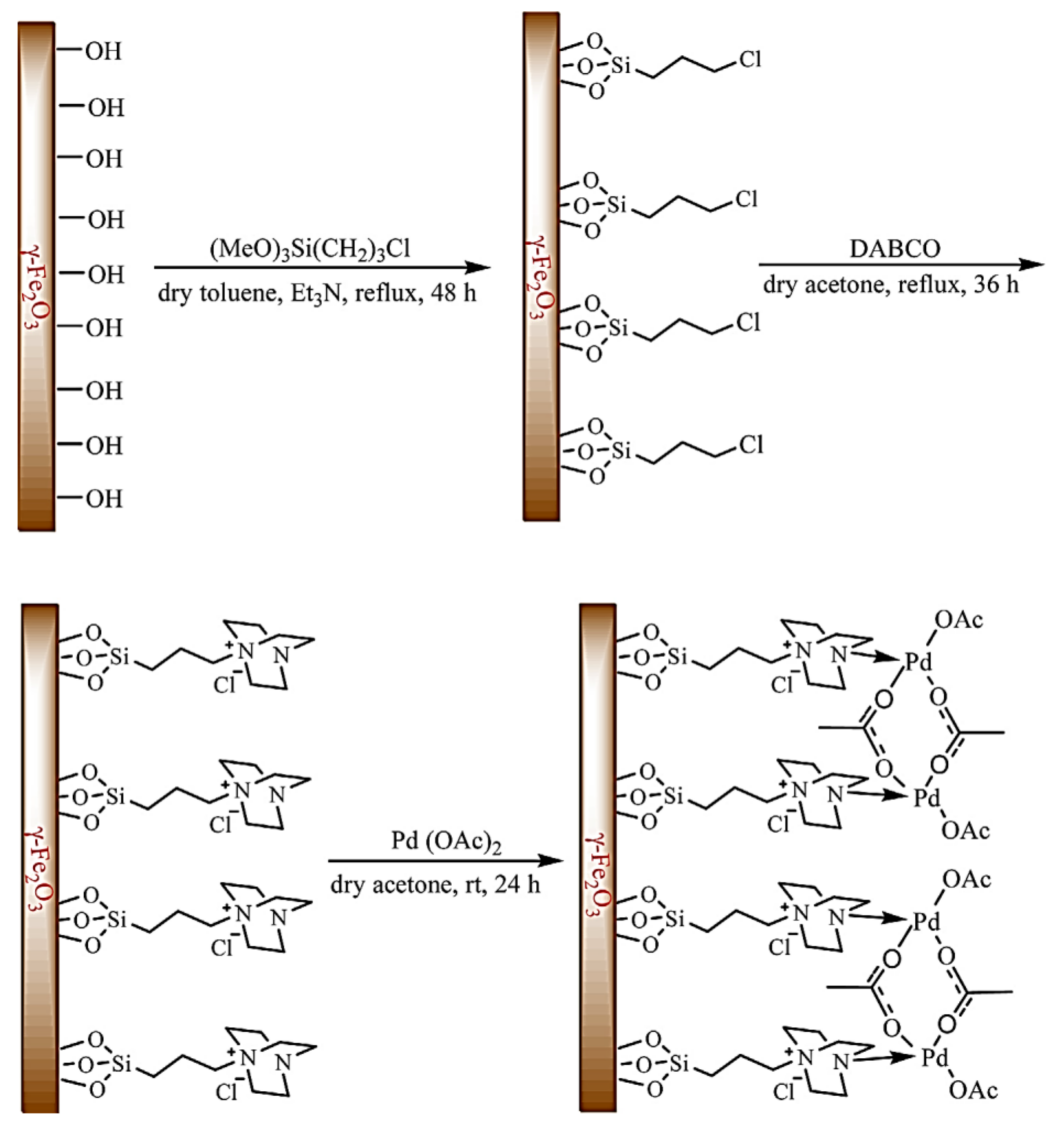
Synthesis of Pd-DABCO-γ-Fe_2_O_3_. Reproduced with permission from [34].

Sobhani and co-workers published a study of a palladium‒DABCO complex supported on γ-Fe_2_O_3_ magnetic NPs [34]. Surface modification of γ-Fe_2_O_3_ magnetite NPs with 3-chloropropyltrimethoxysilane, followed by covalent connection with DABCO, led to a fixed ligand system that was coordinated to palladium(II) (Scheme 27).

**Scheme 28:**
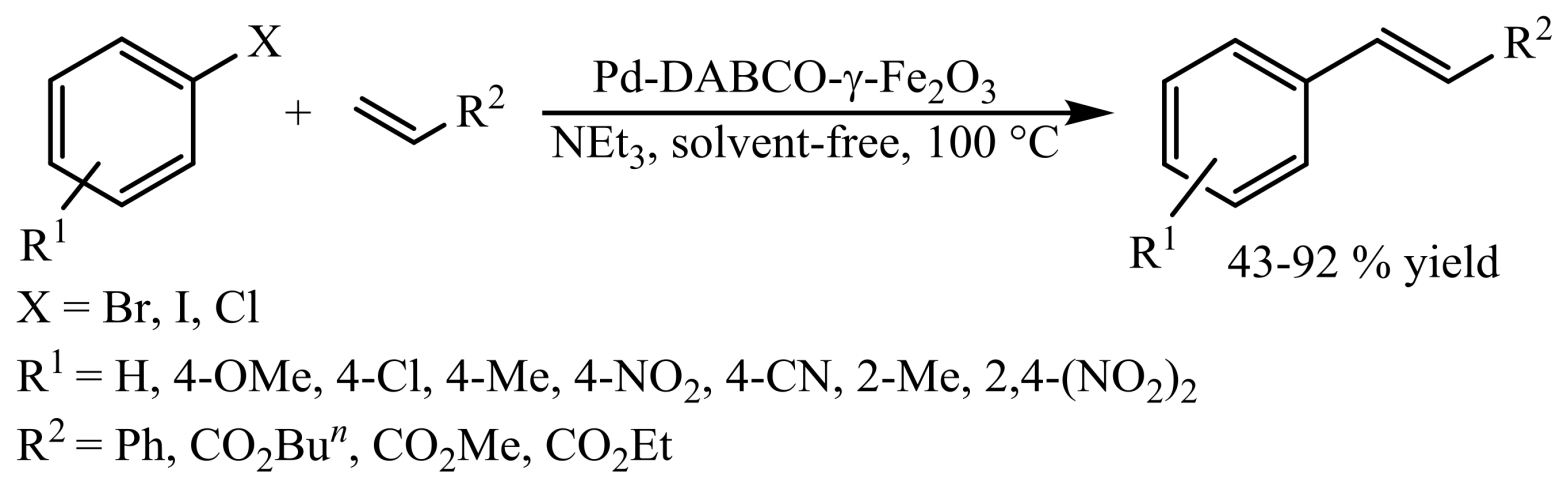
Heck cross-coupling reaction in the presence of Pd-DABCO-γ-Fe_2_O_3_ catalyst [34].

The catalyst Pd-DABCO-γ-Fe_2_O_3_ showed high conversions in Heck cross-coupling reaction of aryl halides with alkyl acrylates and styrene. Under optimized reaction conditions, triethylamine (Et_3_N) was used as the base at 100 °C, and excellent yields were obtained in solvent-free conditions (Scheme 28). This catalyst system could be reused for at least five runs with little loss of activity; the average isolated yield for five consecutive runs was 90%.

**Scheme 29:**
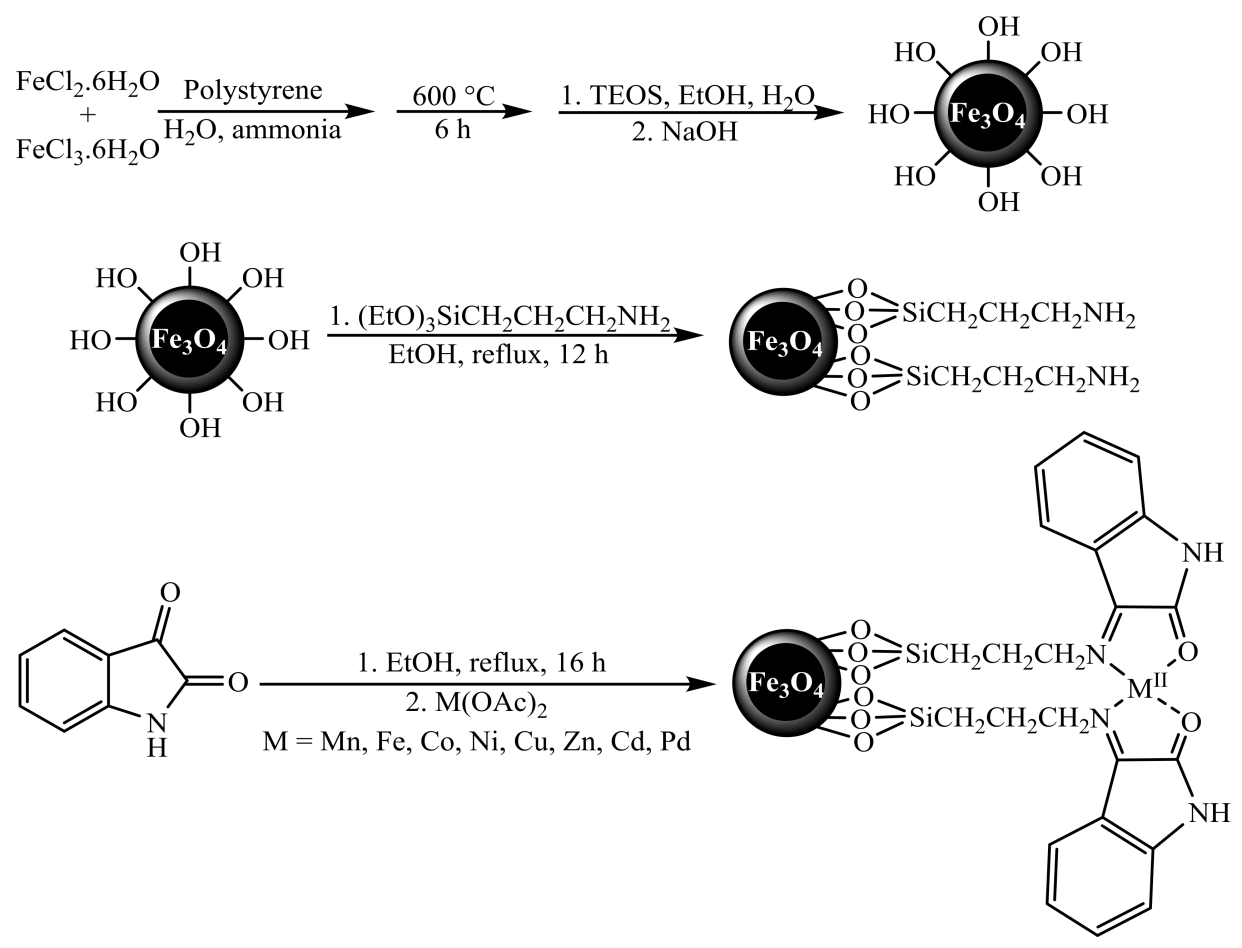
Preparation process of Schiff base complexes functionalized Fe_3_O_4_@SiO_2_ NPs. Reproduced with permission from [35].

Javidi and co-workers synthesized a series of magnetically separable catalysts for Sonogashira‒Hagihara coupling reactions based on M(II) Schiff base complexes (M = Zn, Mn, Cd, Co, Cu, Ni, Fe, and Pd). Fe_3_O_4_ NPs were synthesized by a co-precipitation method using polystyrene as the surfactant and aqueous NH_3_ as the precipitant. Modification of Fe_3_O_4_ NPs via the Stober method using tetraethylorthosilicate (TEOS) resulted in preparation of Fe_3_O_4_@SiO_2_ NPs. Surface functionalization of obtained NPs with Schiff base complexes of various transition metals gave Fe_3_O_4_@SiO_2_/Schiff base/M(II) microspheres (Scheme 29) [35].

**Scheme 30:**
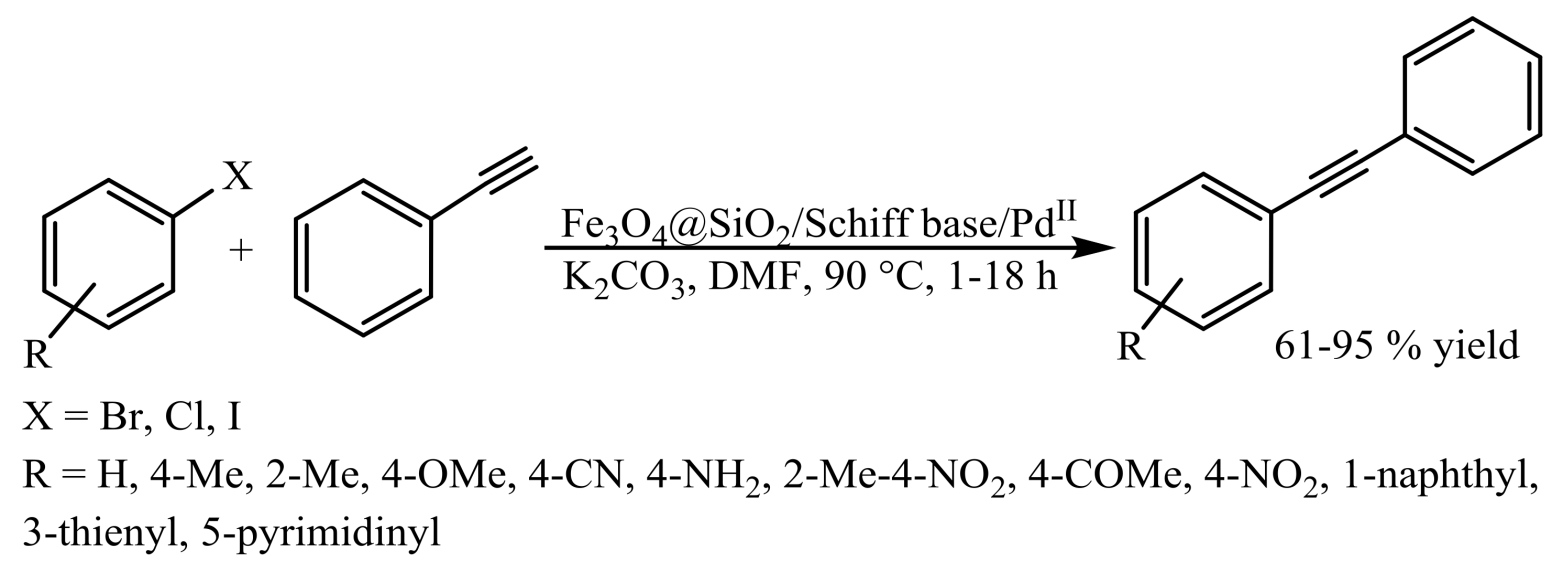
Sonogashira‒Hagihara coupling in the presence of Fe_3_O_4_@SiO_2_/Schiff base/Pd(II) [35].

The obtained catalyst was applied in the copper- and phosphine-free Sonogashira coupling reaction of aryl halides with phenylacetylene in the presence of K_2_CO_3_ in DMF at 90 °C (Scheme 30). Due to the high saturation magnetization of complexes, the catalyst was recovered by a magnetic bar and could be reused six times without significant loss in catalytic activity.

**Scheme 31:**
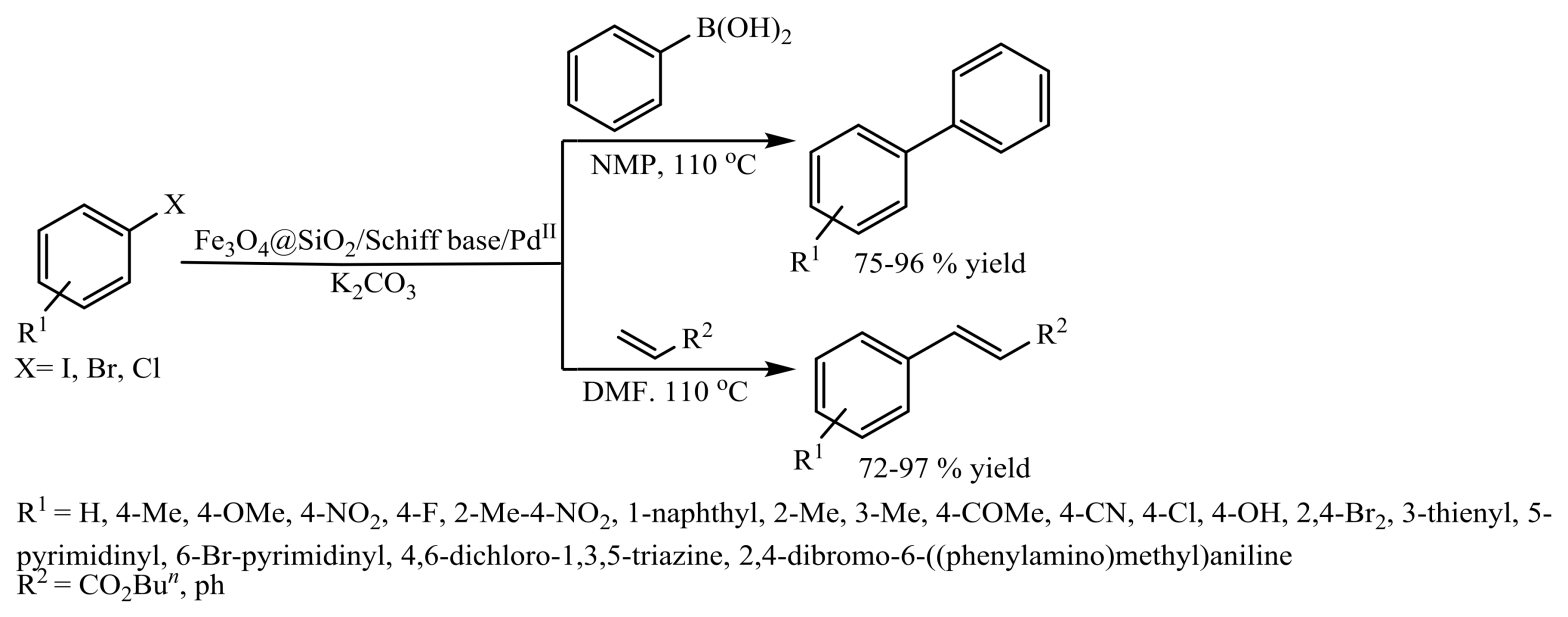
Heck and Suzuki‒Miyaura coupling reactions in the presence of Fe_3_O_4_@SiO_2_/Schiff base/Pd(II) catalyst [36].

In a separate study, the activity of Fe_3_O_4_@SiO_2_/Schiff base/Pd(II) as catalyst for coupling reactions of aryl halides with alkenes (Heck reaction) and phenylboronic acids (Suzuki‒Miyaura reaction) was investigated. The use of this catalyst in the reaction has notable advantages, including heterogeneous nature of the catalyst, excellent yields, short reaction times, easy preparation, simplicity of operation, and cleaner reaction profiles. Also, recycling studies of the catalyst in Heck and Suzuki‒Miyaura reactions revealed that the magnetic catalyst can be reused for eight runs with a slight decline in its catalytic activity (Scheme 31) [36].

**Scheme 32:**
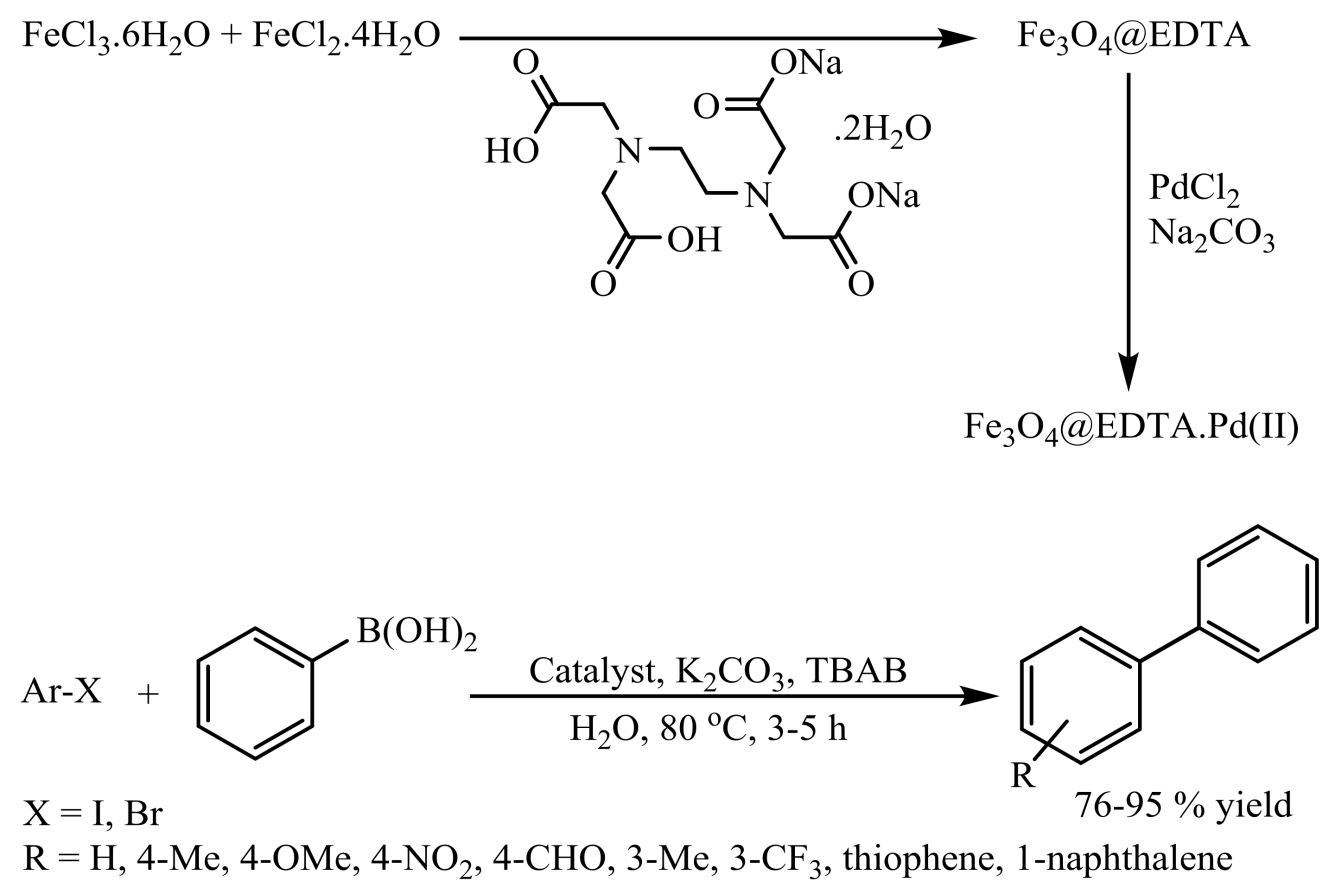
Preparation of Fe_3_O_4_@EDTA-PdCl_2_ and Suzuki‒Miyaura reaction in the presence of the obtained catalyst [37].

**Figure 6:**
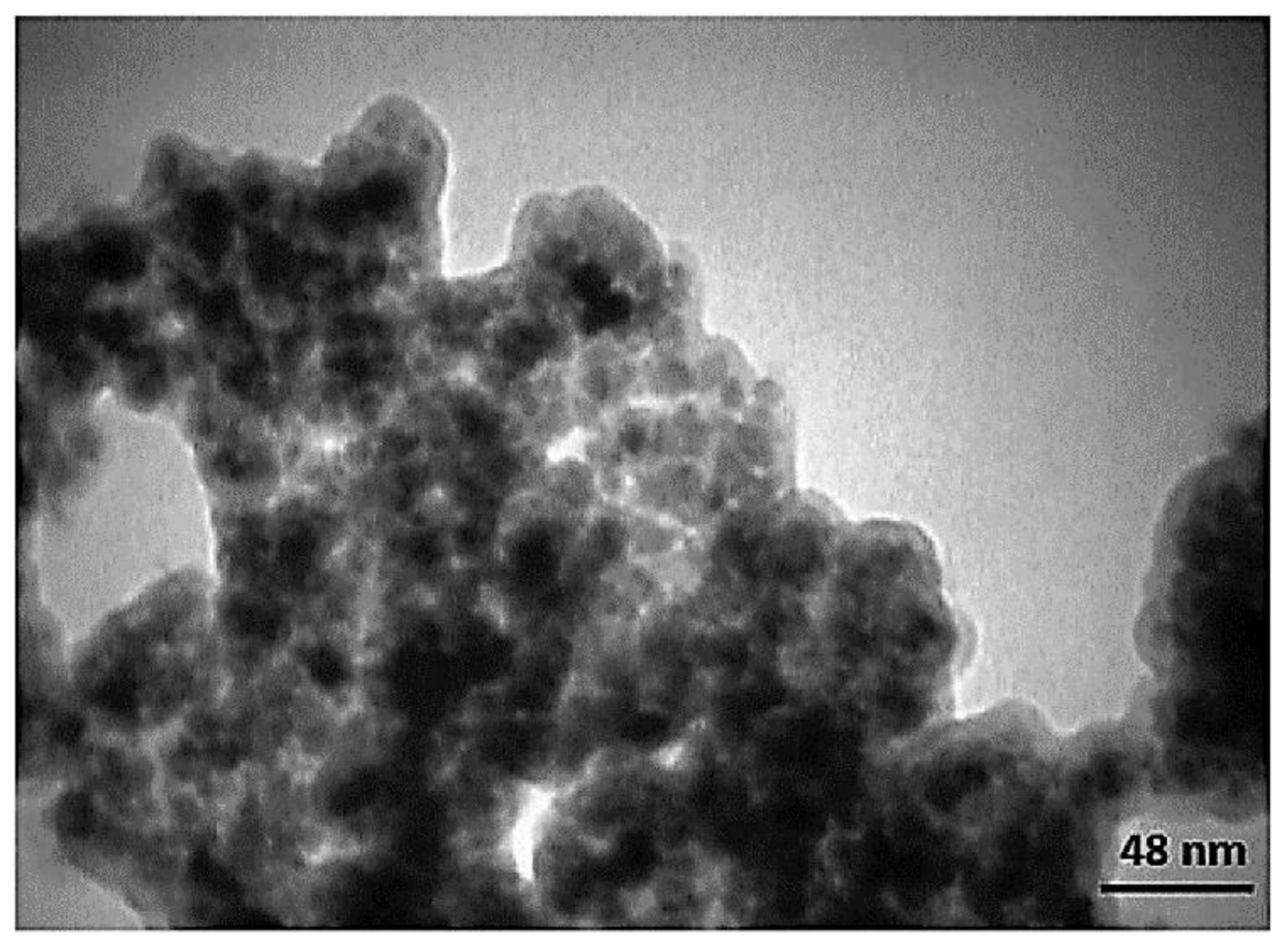
Transmission electron micrograph of Fe_3_O_4_@EDTA-PdCl_2_. Reproduced with permission from [37].

The synthesis of Fe_3_O_4_@EDTA-Pd(II) (EDTA = ethylenediaminetetraacetic acid) by encapsulation of Pd(II) into superparamagnetic NPs grafted with EDTA, and its application in Suzuki‒Miyaura coupling reaction and reduction of nitro compounds in water was investigated (Scheme 32) [37]. In this work, for the first time, the possibility of using magnetic EDTA as a support for immobilizing metal catalyst was investigated. A TEM image of the obtained catalyst revealed NPs with spherical morphology (Figure 6). Furthermore, the catalyst can be recovered by use of external magnet and reused up to five times with only a very slight decrease in its activity [37].

**Scheme 33:**
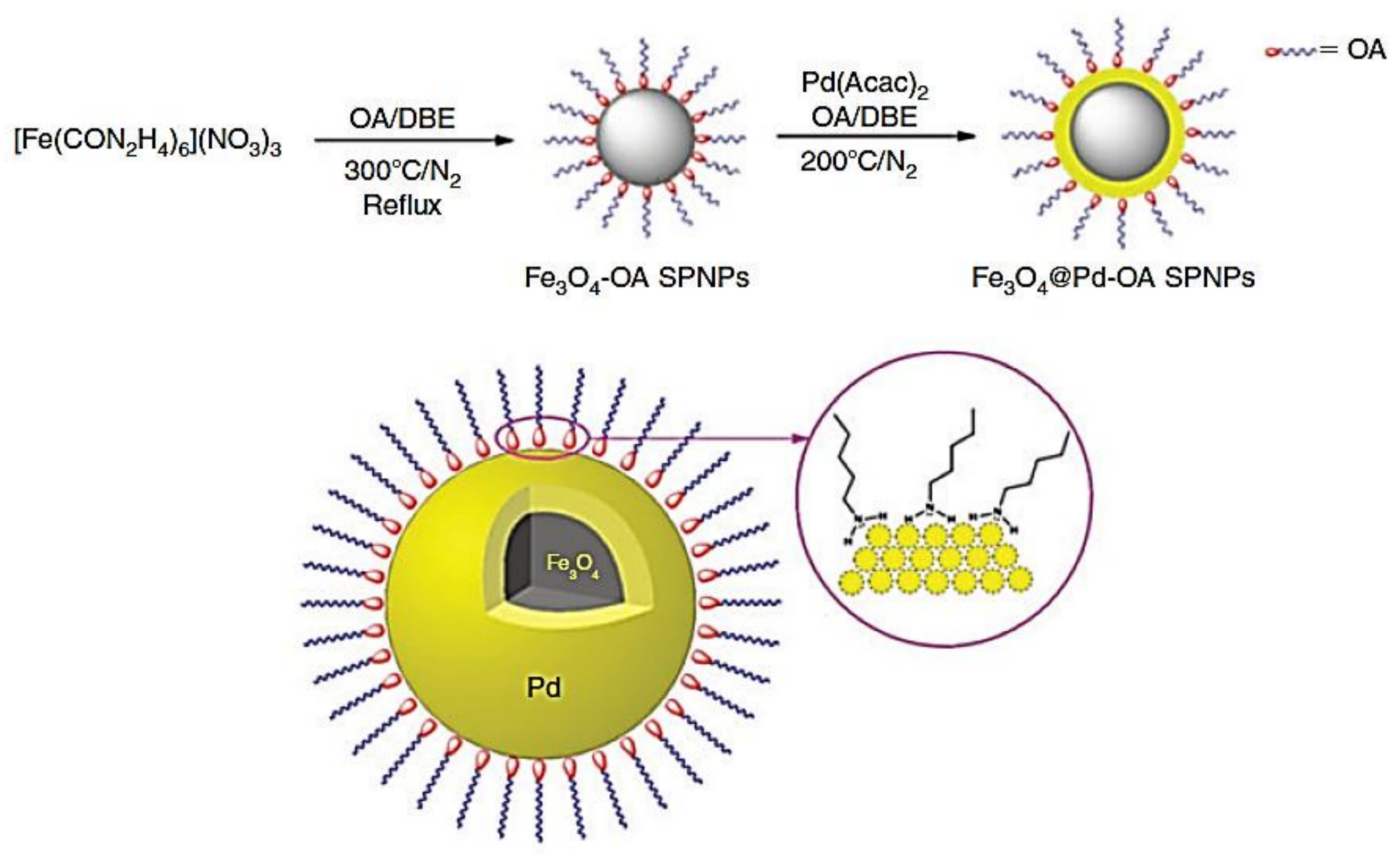
Synthesis of precursors, Fe_3_O_4_-OA and Fe_3_O_4_@Pd-OA superparamagnetic nanoparticles (SPNPs) (OA, oleylamine; DBE, dibenzylether). Reproduced with permission from [38].

Cappelletti et al. reported the synthesis of a Pd catalyst witha superparamagnetic Fe_3_O_4_ core and a very thin Pd shell [38]. Fe_3_O_4_ superparamagnetic NPs (SPNPs) were synthesized through a new strategy whereby very small and narrow size distribution Fe_3_O_4_ NPs were obtained via replacing iron(III) acetylacetonate (Fe(Acac)_3_) as a metal source with an iron‒urea complex (Fe-urea). In this approach, OA was used for stabilization of the Pd(0) shell (Scheme 33).

**Scheme 34:**
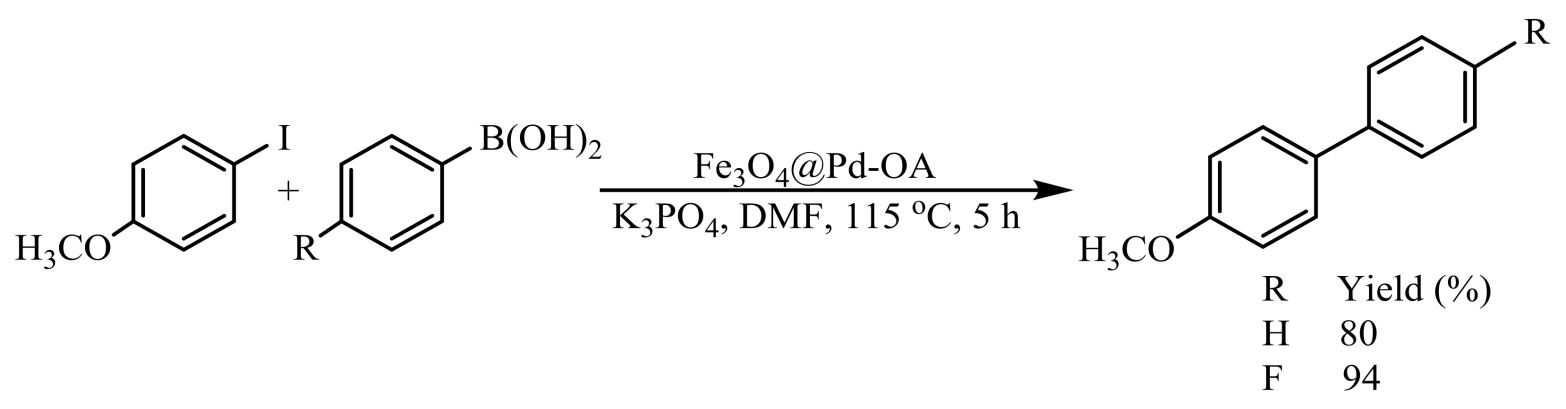
Suzuki‒Miyaura coupling reaction catalyzed by Fe_3_O_4_@Pd-oleylamine (OA) superparamagnetic. Reproduced with permission from [38].

The resulting catalyst exhibits promising results in the Suzuki‒Miyaura coupling reaction of *p*-iodoanisole with boronic acids (Scheme 34). Additionally, the catalyst can be reused for at least four cycles without significant loss in catalytic activity.

**Scheme 35:**
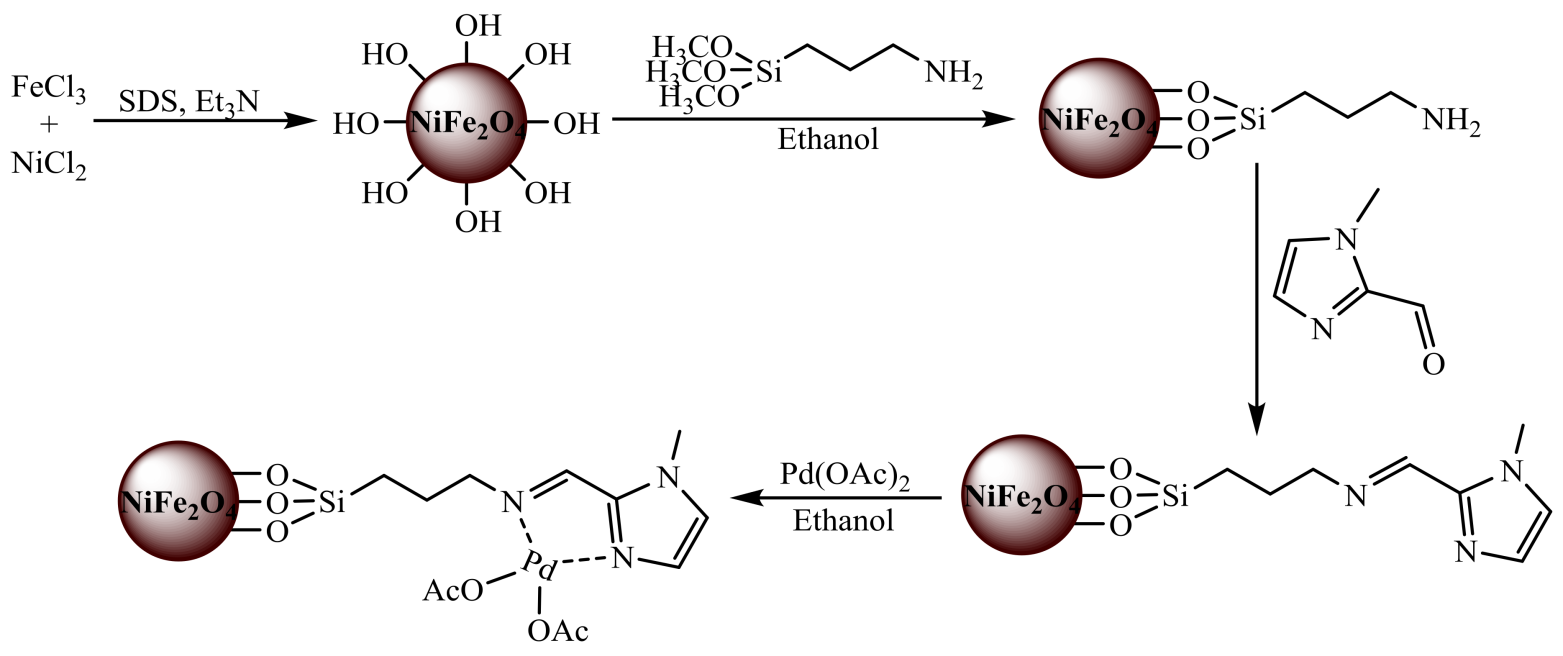
Synthesis of the palladium catalyst anchored on magnetic NPs. Reproduced with permission from [39].

**Figure 7:**
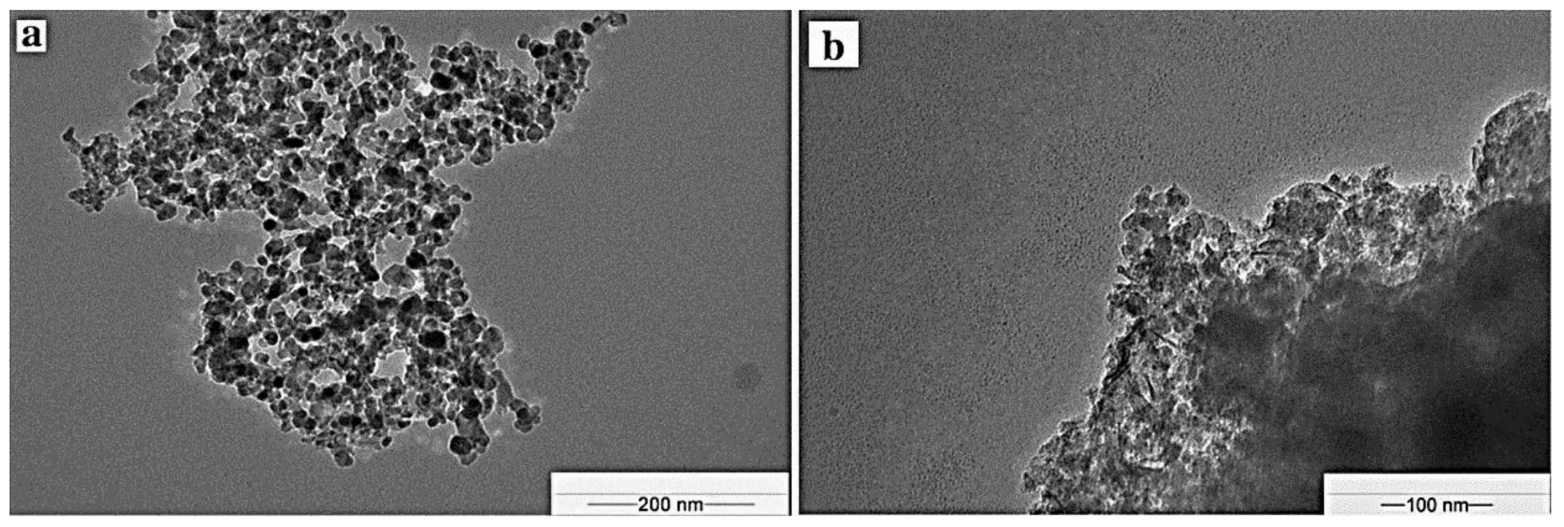
TEM images of (**a**) NiFe_2_O_4_ NPs, (**b**) Pd(II)-NiFe_2_O_4_ catalyst. Reproduced with permission from [39].

Singh and co-workers developed a protocol for the immobilization of Pd NPs on NiFe_2_O_4_ as a heterogeneous catalyst for Suzuki‒Miyaura coupling reaction [39]. NiFe_2_O_4_ NPs were obtained using a microemulsion method. Functionalization of prepared NiFe_2_O_4_ with Schiff base group demonstrated immobilized bidentate ligand for complexation with Pd (Scheme 35). TEM images of NiFe_2_O_4_ and Pd(II)-NiFe_2_O_4_ are given in Figure 7, observed aggregation in Pd(II)-NiFe_2_O_4_ is due to the functionalization of surface with organic materials.

**Scheme 36:**
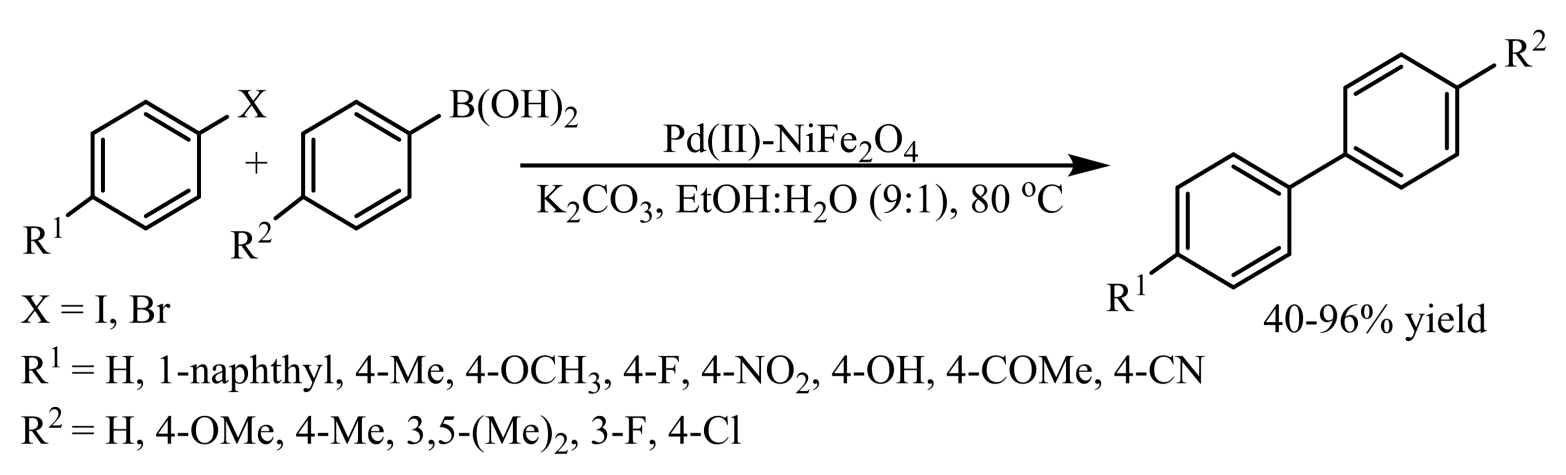
Suzuki‒Miyaura coupling reaction catalyzed by Pd(II)-NiFe_2_O_4_.

The as-prepared catalyst can be applied in coupling reaction of aryl halides with arylboronic acids in the presence of K_2_CO_3_ in EtOH/H_2_O (9:1) at 80 °C (Scheme 36). Furthermore, the reusability of the catalyst has been tested for up to five runs with a slight decrease in catalytic activity (99% to 95%) [39].

**Scheme 37:**
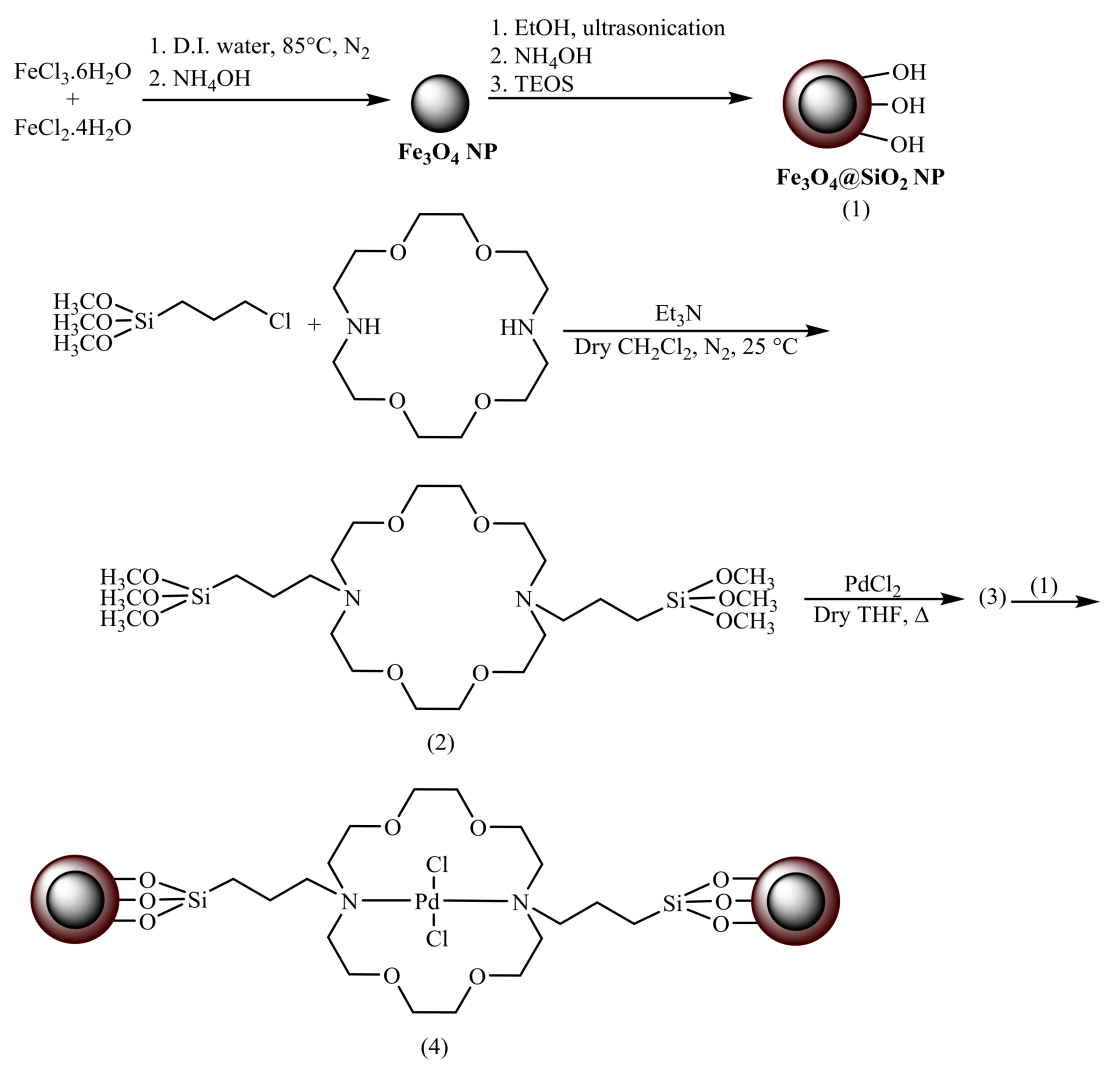
Synthetic schemes of Fe_3_O_4_ NPs and Fe_3_O_4_@SiO_2_ (1), [(MeO)_3_Si-Pr]_2_-C22-Pd(II) complex (3), and Fe_3_O_4_@SiO_2_@C22-Pd(II) catalyst (4) [40].

Movassagh and co-workers, by covalent grafting of a trimethoxysilyl-functionalized C22-Pd(II) complex on silica-coated magnetic nanoparticles, prepared a magnetic nanocatalyst and investigated its catalytic activity in the Suzuki‒Miyaura coupling reaction and the formation of aryl-sulfur bonds (Scheme 37) [40].

**Scheme 38:**
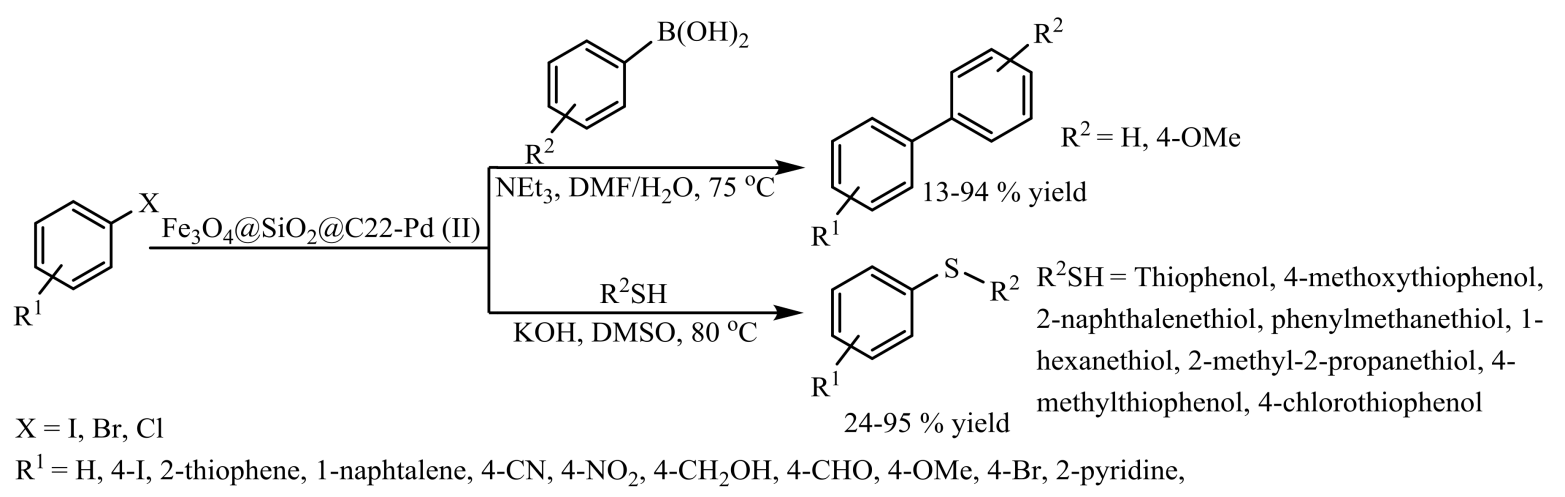
Suzuki‒Miyaura and *S*-arylation coupling reaction catalyzed by Fe_3_O_4_@SiO_2_@C22-Pd(II) [40].

In the presence of 0.5 mol % of this catalyst, the best results for the coupling of aryl halides with arylboronic acids were obtained with NEt_3_ as the base in DMF/H_2_O (1:1) at 75 °C. This magnetic Pd(II)-cryptand 22 complex also showed good activity in the *S*-arylation coupling reaction in DMSO at 80 °C (Scheme 38). The use of this catalyst in the reaction offers several advantages, including excellent yields, short reaction time, simplicity of operation, easy separation and recyclability of the magnetic catalyst, and the ability to tolerate a wide variety of substitutions in the reagents. Moreover, the magnetic nanocatalyst could easily be recovered with no considerable change in the activity for five successive runs by magnetic separation, washing with EtOH, and air-drying.

**Scheme 39:**
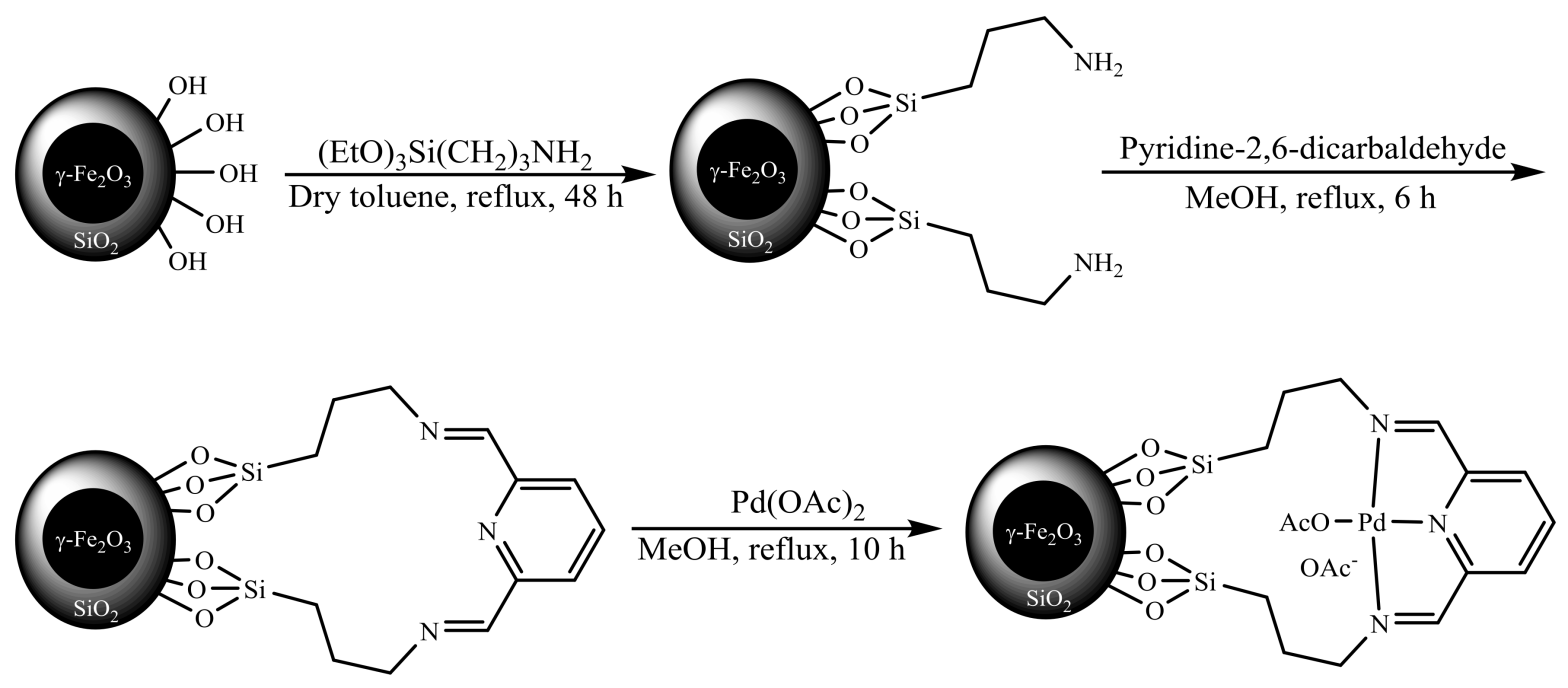
Synthesis of Pd-BIP-γ-Fe_2_O_3_@SiO_2_. Reproduced with permission from [41].

**Figure 8:**
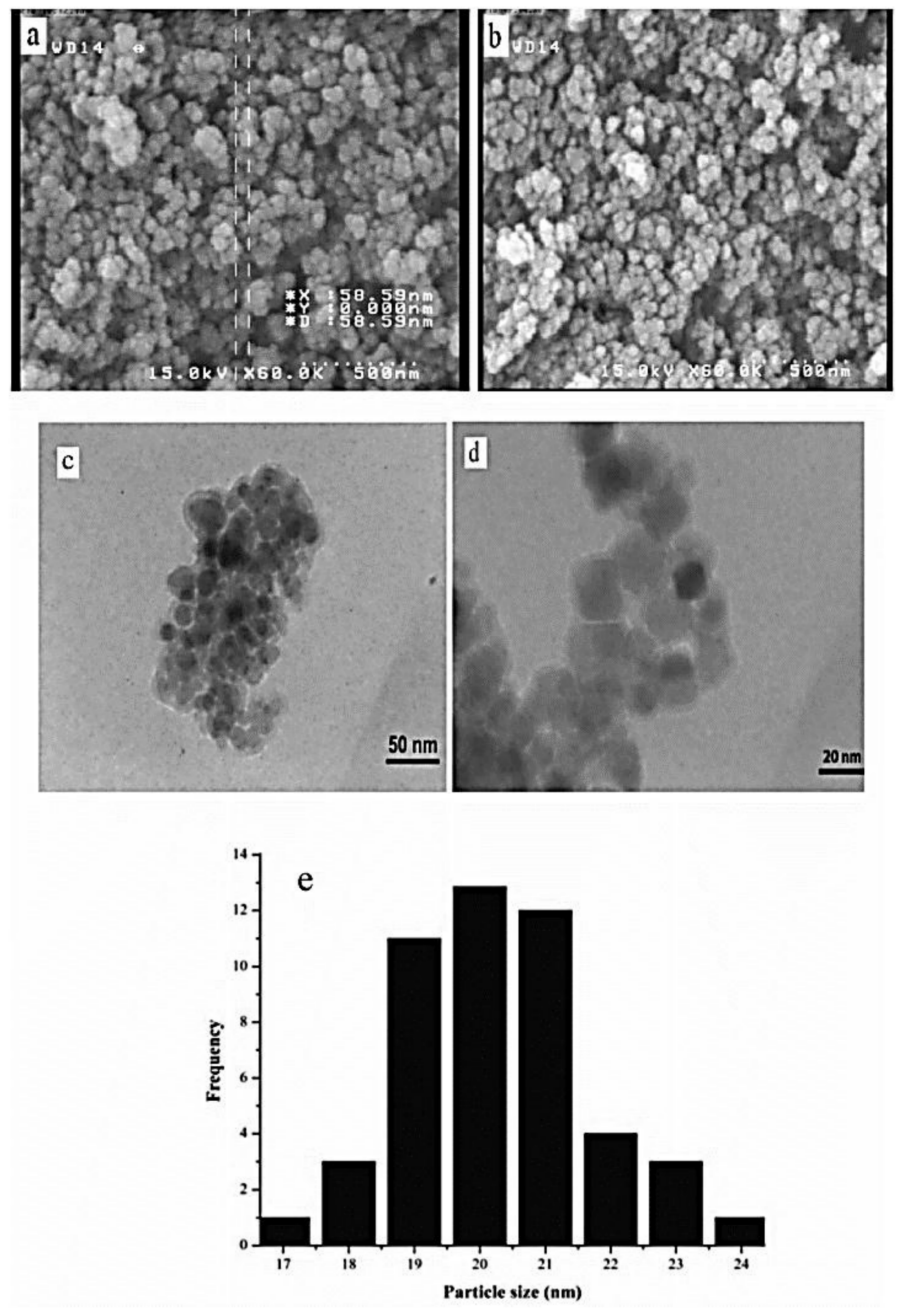
(**a**,**b**) SEM images, (**c**,**d**) TEM images and (**e**) particle size distribution histogram of Pd-BIP-γ-Fe_2_O_3_@SiO_2_. Reproduced with permission from [41].

Sobhani and co-workers demonstrated that pyridine-2,6-dicarbaldehyde can be anchored on the surfaces of amino functionalized γ-Fe_2_O_3_@SiO_2_ magnetic NPs and form a bis(imino)pyridine as an NNN pincer ligand to stabilize Pd NPs; this report presents the preparation of magnetic Pd-catalyst for Heck, Suzuki‒Miyaura, and Sonogashira coupling reactions (Scheme 39) [41]. As shown in Figure 8, the nanocatalyst contained uniform, spherical morphology particles with an average diameter of about 20 nm. TEM images of Pd-BIP-γ-Fe_2_O_3_@SiO_2_ displayed a dark γ-Fe_2_O_3_ core surrounded by a gray silica shell with a thickness of about 4 nm.

**Scheme 40:**
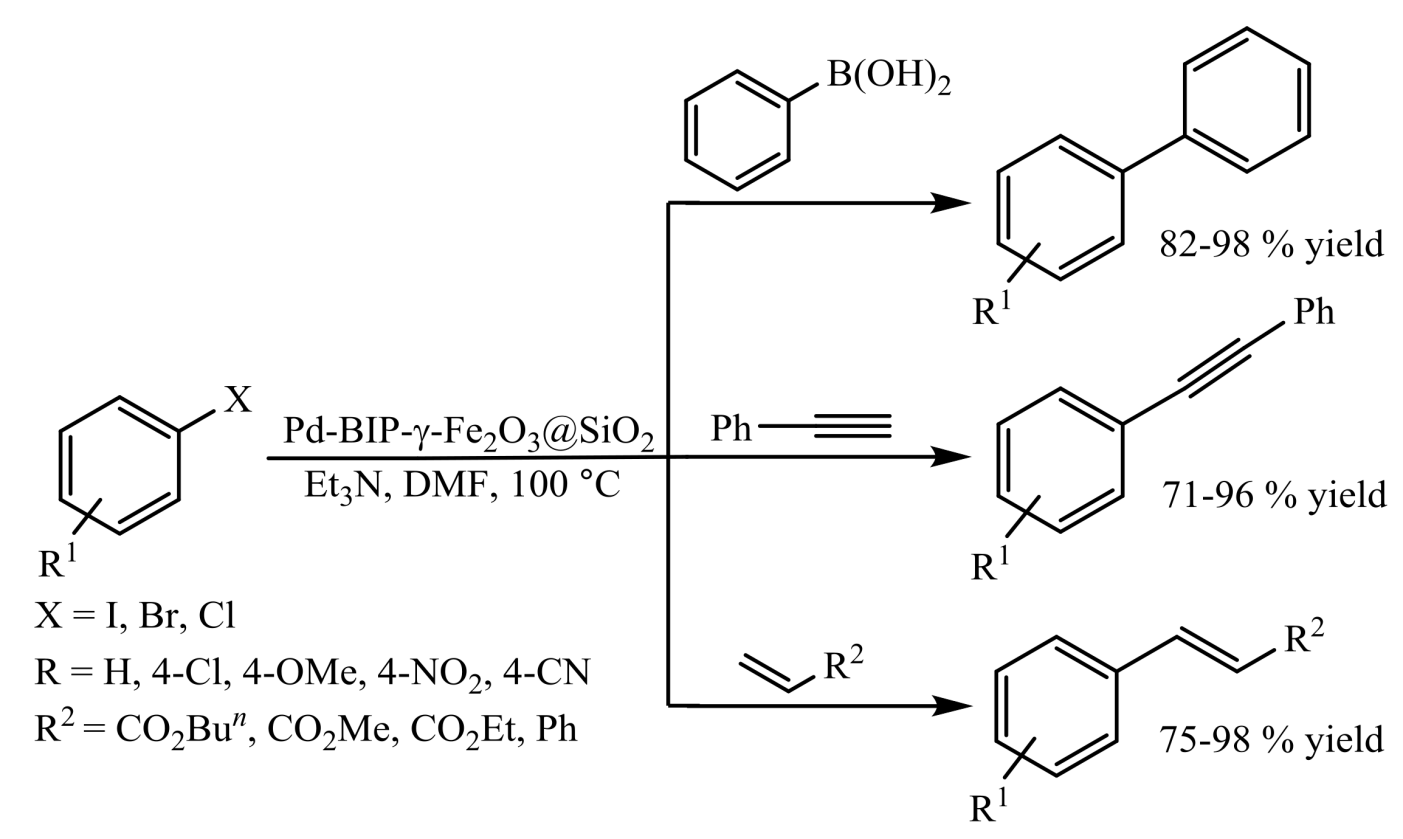
Heck, Suzuki‒Miyaura, and Sonogashira coupling reactions catalyzed by Pd-BIP-γ-Fe_2_O_3_@SiO_2_ [41].

**Figure 9:**
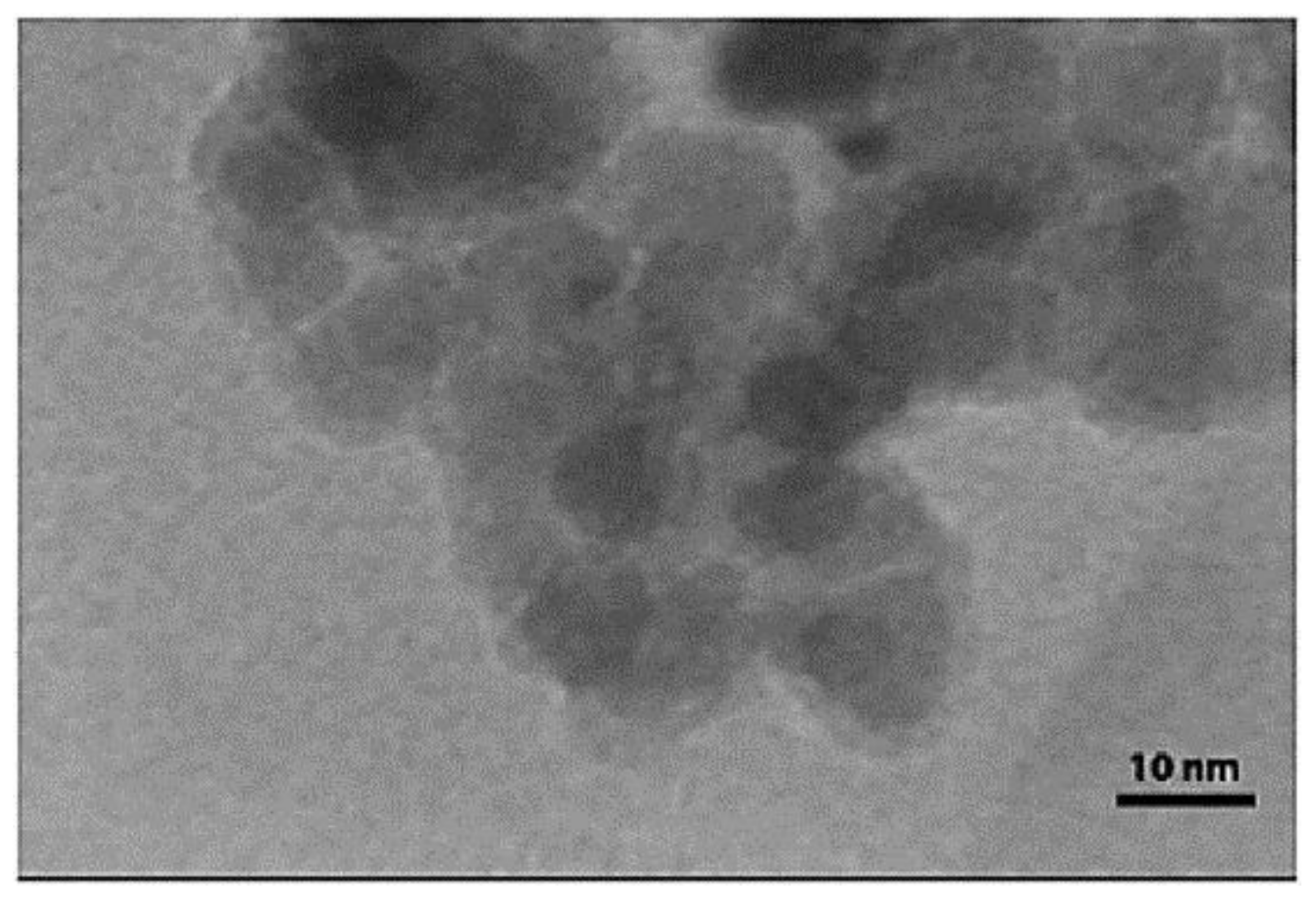
TEM of Pd-BIP-γ-Fe_2_O_3_@SiO_2_ after the tenth reuse. Reproduced with permission from [41].

The air- and moisture-stable Pd catalyst was used in carbon‒carbon coupling reactions of aryl halides with olefins, phenylboronic acid and phenylacetylene via Heck, Suzuki‒Miyaura, and Sonogashira reactions. A wide range of aryl halides (iodides, bromides, and chlorides) were coupled successfully with alkyl acrylates, styrene, phenylboronic acid, and phenylacetylene to generate the corresponding products in good to high yields (Scheme 40). Pd-BIP-γ-Fe_2_O_3_@SiO_2_ could be reused 10 times without any significant loss of its catalytic activity. The TEM image of the catalyst after 10 reuses showed well-dispersed Pd NPs with no evident aggregation (Figure 9) [41].

**Scheme 41:**
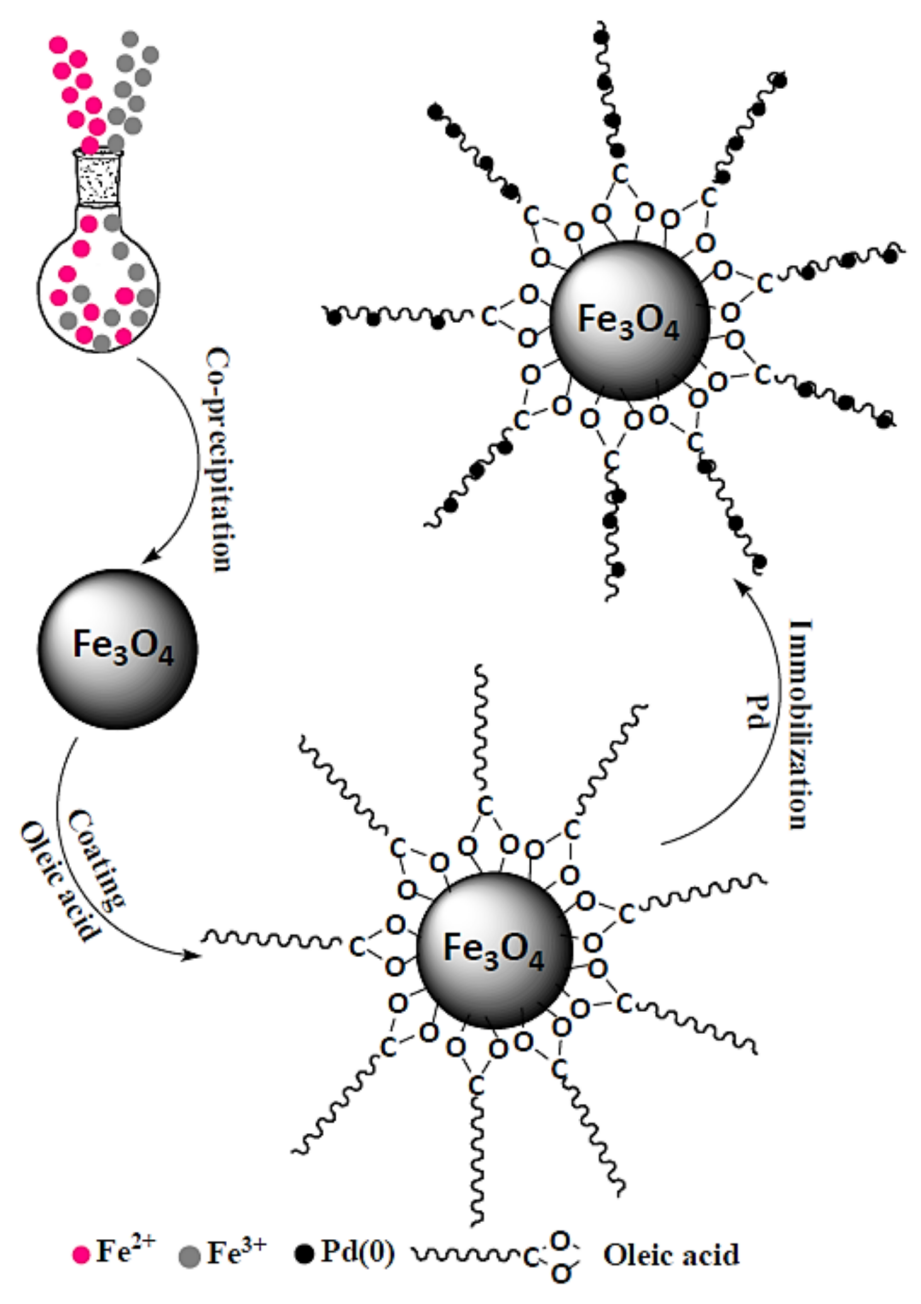
Schematic of the preparation of the Fe_3_O_4_/oleic acid/Pd nanocatalyst. Reproduced with permission from [42].

**Figure 10:**
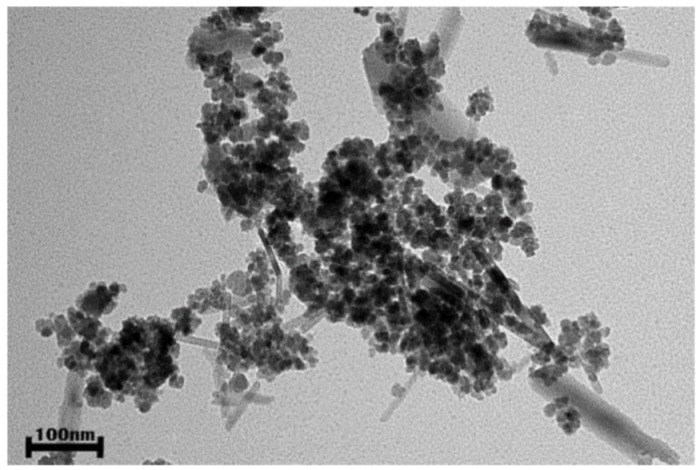
TEM image of Fe_3_O_4_/oleic acid/Pd. Reproduced with permission from [42].

Karami and co-workers describe a recyclable oxime-derived palladacycle immobilized on a highly active Fe_3_O_4_/oleic acid solid support for the copper-free Sonogashira cross-coupling reaction [42]. The procedure for the preparation of catalyst is shown in Scheme 41. First, oleic acid-coated Fe_3_O_4_ NPs were synthesized and then oxime-derived palladacycle was added to a mixture of Fe_3_O_4_/oleic acid and ethanol; finally, hydrazine hydrate was dropped onto the mixture to generate a Fe_3_O_4_/oleic acid/Pd catalyst. A TEM image of catalyst revealed that the palladium NPs were well dispersed on the surface of the Fe_3_O_4_/oleic acid NPs with diameters from 5 to 10 nm and an average size of about 9.97 nm (Figure 10).

**Scheme 42:**
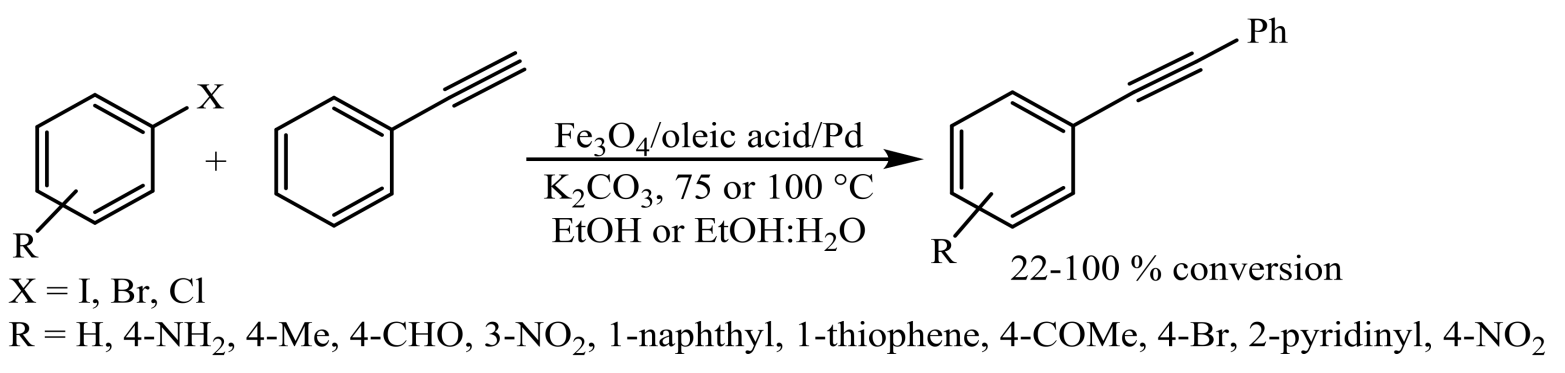
Sonogashira cross-coupling reactions of various aryl halides with phenylacetylene catalyzed by Fe_3_O_4_/oleic acid/Pd [42].

The activity of these supported Pd catalysts has been evaluated in a copper-free Sonogashira cross-coupling reaction between various aryl halides with phenylacetylene, whose products were obtained in high yields (Scheme 42). The Fe_3_O_4_/oleic acid/Pd can be reused more than six times without loss of its activity.

**Scheme 43:**
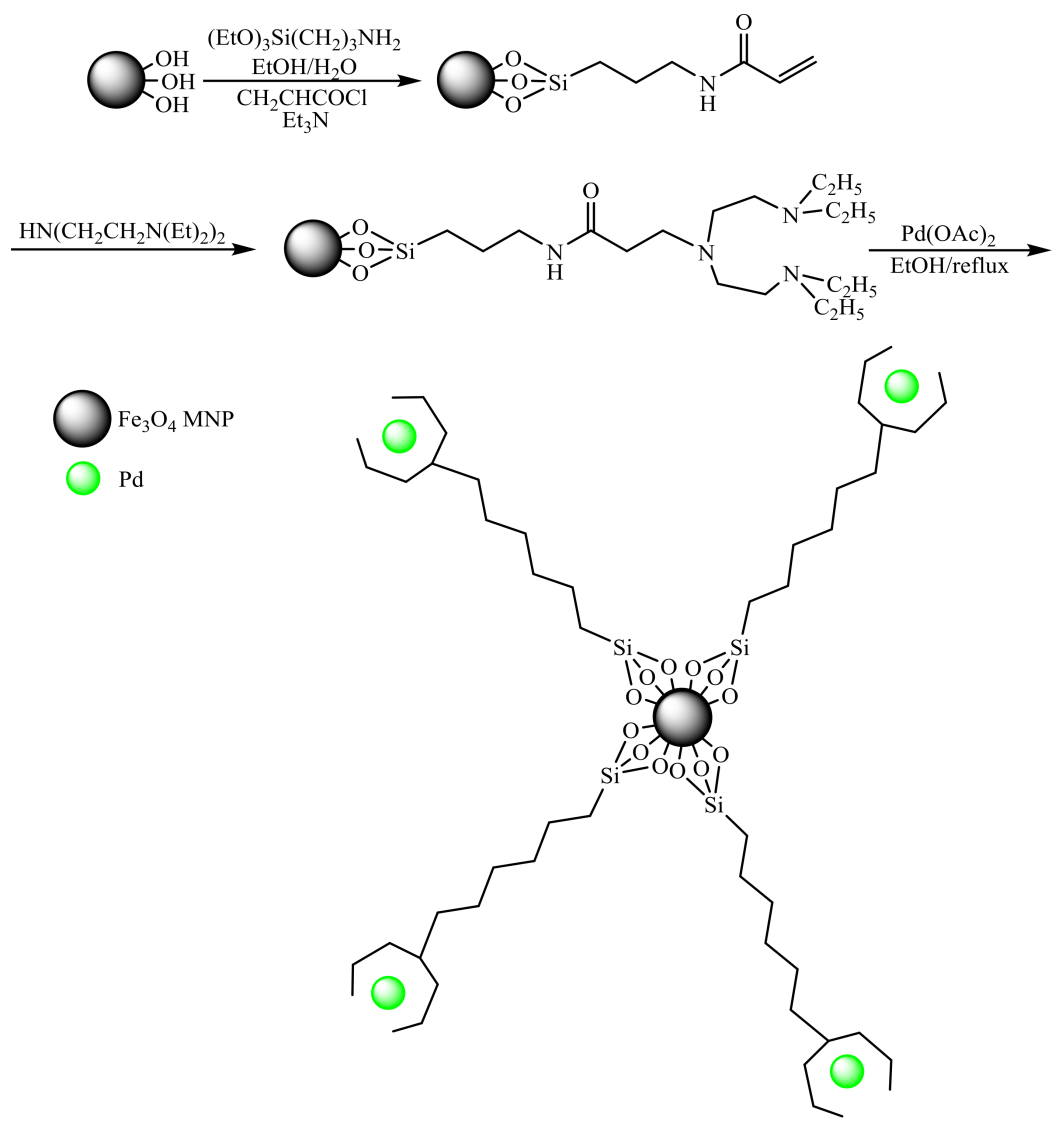
Synthesis of Fe_3_O_4_@PTA-Pd nanocatalyst [43].

Ghorbani-Choghamarani and co-workers modified the surface of silica-coated Fe_3_O_4_ NPs with a tridentate‒Pd complex. Magnetic NPs were prepared through the coprecipitation method; coating these nanoparticles with tridentate ligand gave functionalized magnetic NPs whose subsequent reaction of Pd(OAc)_2_ with these immobilized ligands resulted in Fe_3_O_4_@PTA-Pd as a recyclable and efficient nanocatalyst (Scheme 43) [43]. The focus of the present research was on the investigation of the recycling, reusability, and stability of the catalyst.

**Scheme 44:**
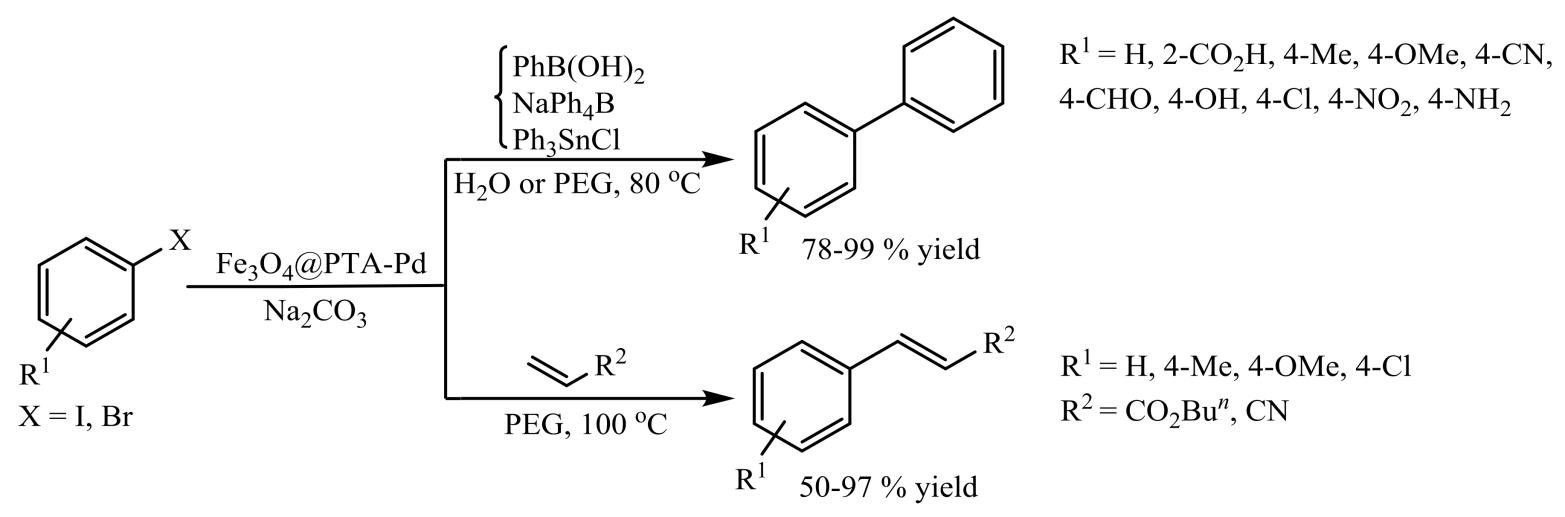
Suzuki‒Miyaura, Stille, and Heck cross-coupling reactions catalyzed by Fe_3_O_4_@PTA-Pd nanocatalyst [43].

The Fe_3_O_4_@PTA-Pd nanocatalyst was applied in Suzuki‒Miyaura, Stille, and Heck cross-coupling reactions of aryl halides with tetrephenylborate, phenylboronic acid, and chlorotriphenylstannane in green solvents, which showed corresponding products in good to excellent yields. The reusability of the catalyst was investigated in a final set of experiments: after completion of the reactions, the catalyst was easily separated magnetically and reused at least five times without any significant loss in its activities. ICP-OES analysis of the recycled catalyst showed minor changes in Pd contents (1.64 mmol/g), in which only 1% leaching was observed (Scheme 44).

**Scheme 45:**
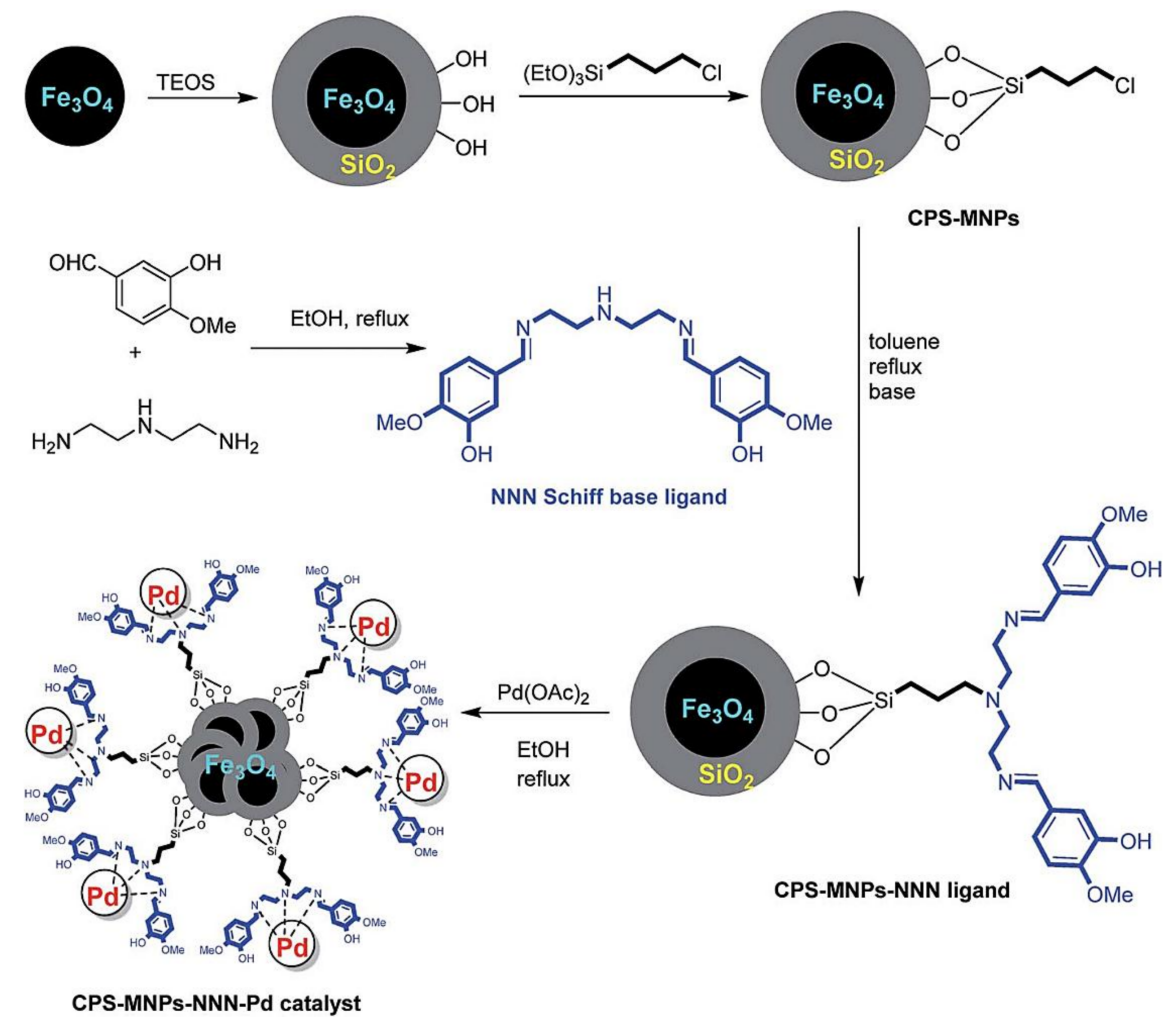
A schematic illustration the preparation of CPS-MNPs-NNN‒Pd catalyst. Reproduced with permission from [44].

**Figure 11:**
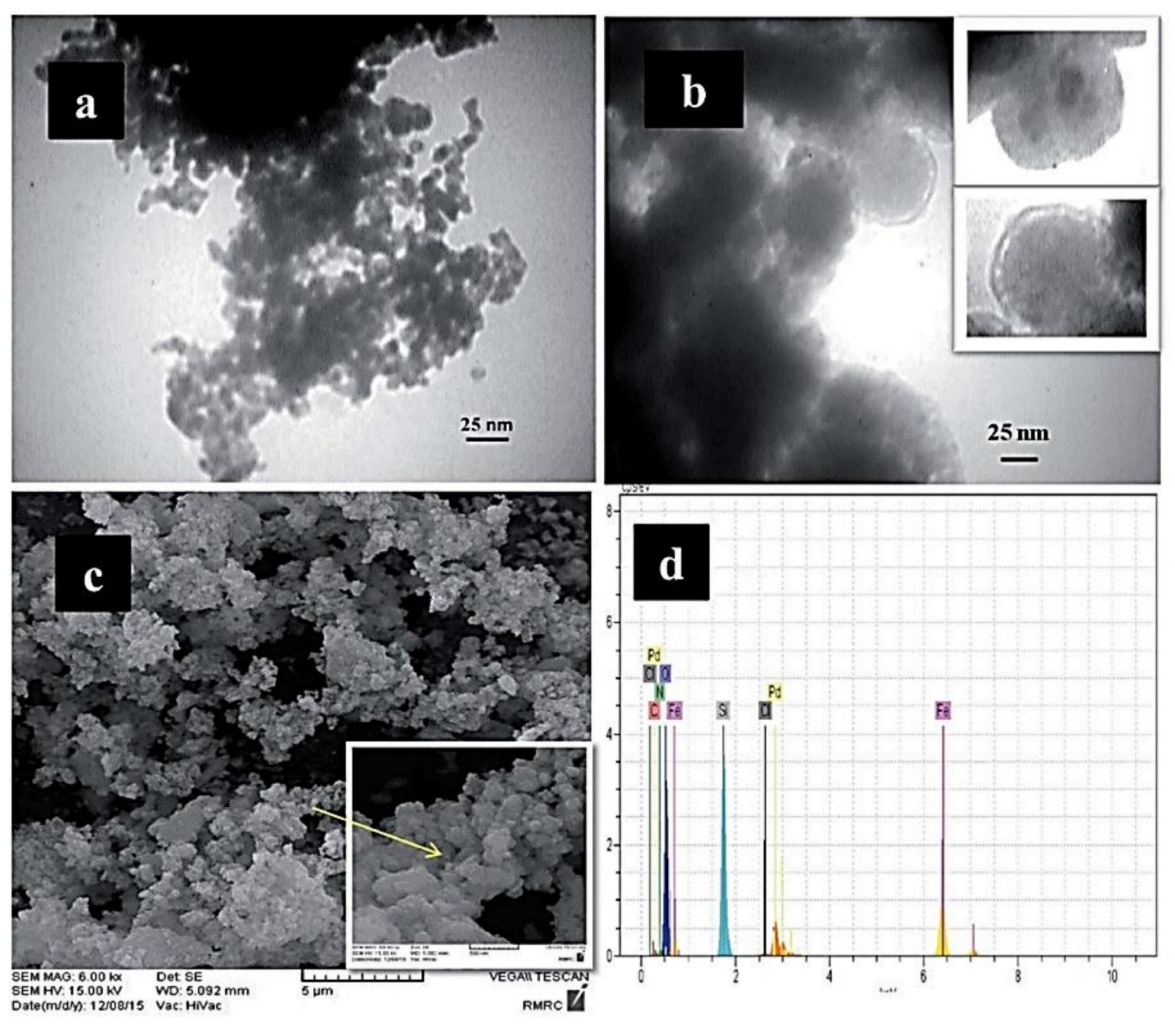
TEM pictures of the CPS-MNPs-NNN‒Pd catalyst (**a**,**b**), SEM image of the CPS-MNPs-NNN‒Pd catalyst (**c**), EDX pattern of CPSMNPs-NNN‒Pd catalyst (**d**). Reproduced with permission from [44].

Asadi and co-workers synthesized a catalyst for Heck and Sonogashira reactions based on NNN Pd-complex supported on silica coated magnetic NPs (MNPs) by treatment of the magnetic NPs with (3-chloropropyl)-trimethoxysilane (3-CPTMS), followed by covalent connection with synthetic NNN ligand, gave a CPS-MNPs-NNN ligand for the formation of a complex with palladium. Finally, immobilization of the palladium species on the CPS-MNPs-NNN ligand surface afforded a CPS-MNPs-NNN‒Pd catalyst (Scheme 45) [44]. TEM images of the prepared catalyst showed that the sizes of the palladium catalyst are in the range of 8–15 nm (Figure 11).

**Scheme 46:**
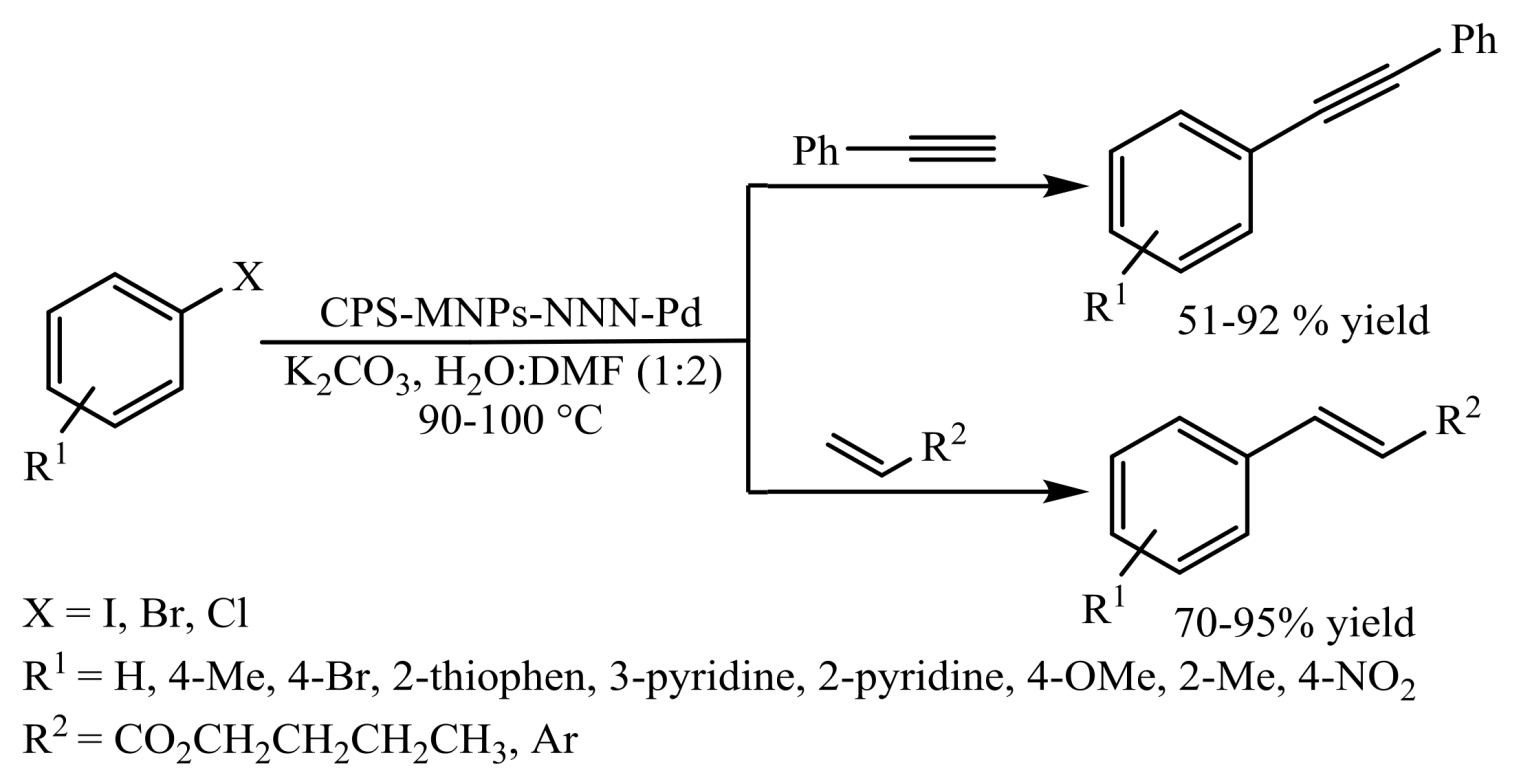
Heck and Sonogashira coupling reactions in the present of CPS-MNPs-NNN‒Pd catalyst [44].

**Figure 12:**
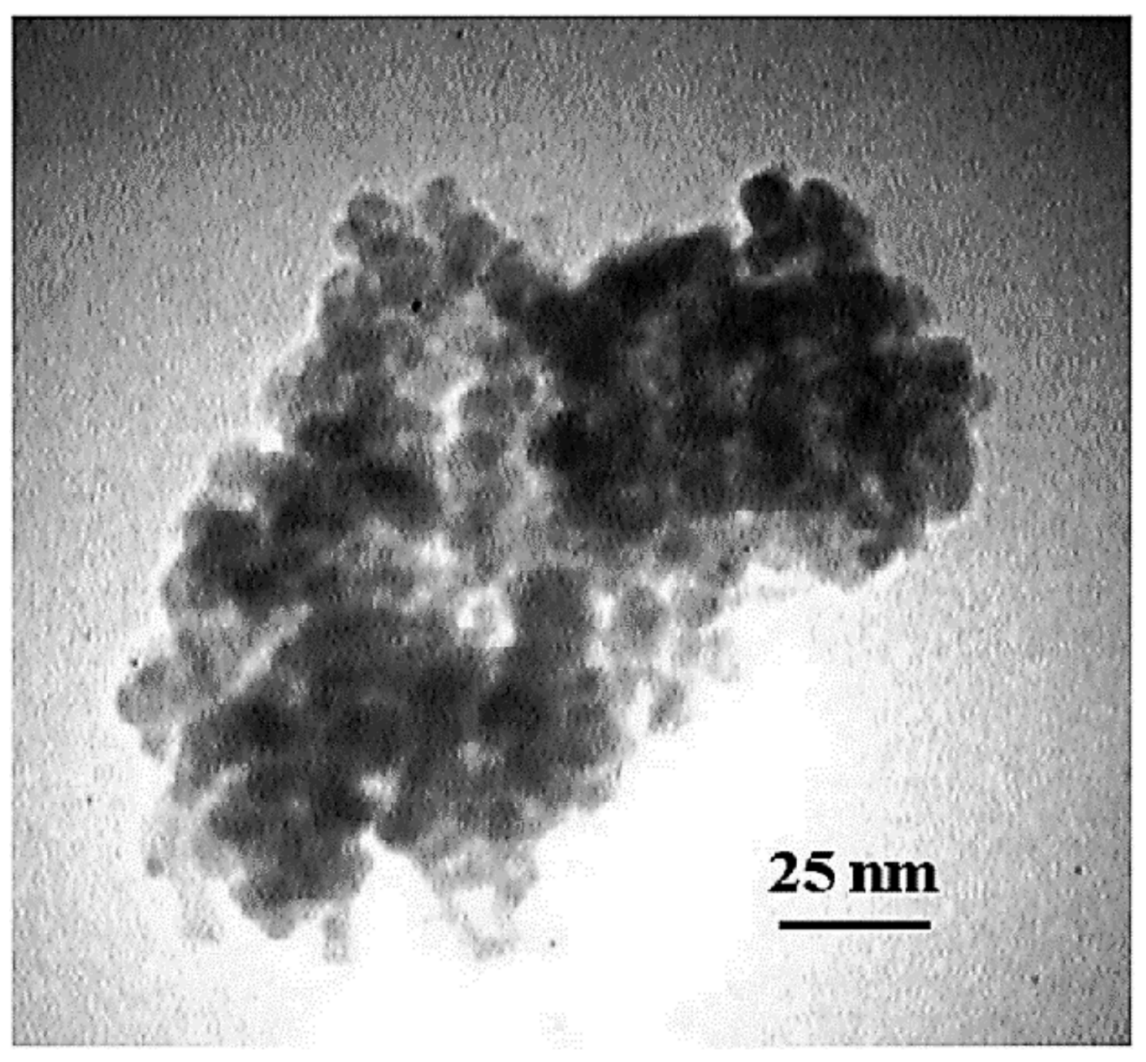
TEM image of CPS-MNPs-NNN‒Pd catalyst after four cycles in the Heck reaction of iodobenzene with *n*-butyl acrylate. Reproduced with permission from [44].

The CPS-MNPs-NNN‒Pd complex, an air- or moisture-stable catalyst was applied in C‒C coupling protocols, namely Heck and Sonogashira reactions. Firstly, the catalyst was tested for Heck coupling reaction of various aryl halides with alkenes in the presence of K_2_CO_3_ as a base in DMF/H_2_O (1:2) at 90 °C. Furthermore, the catalyst was tested in the Sonogashira coupling reaction of various substituted aryl halides with phenylacetylene under similar conditions and generated the corresponding products in good to excellent yields (Scheme 46). A recycling experiment was performed in the Heck reaction of iodobenzene with *n*-butyl acrylate, after magnetic separation of catalyst and washing with ethanol, the catalyst was used directly in the next run and could be recycled at least five times with a negligible loss in its catalytic activity and negligible amount of Pd leaching (0.3 ppm). The TEM image of used catalyst in Figure 12 shows no morphology change of the catalyst after four runs.

**Scheme 47:**
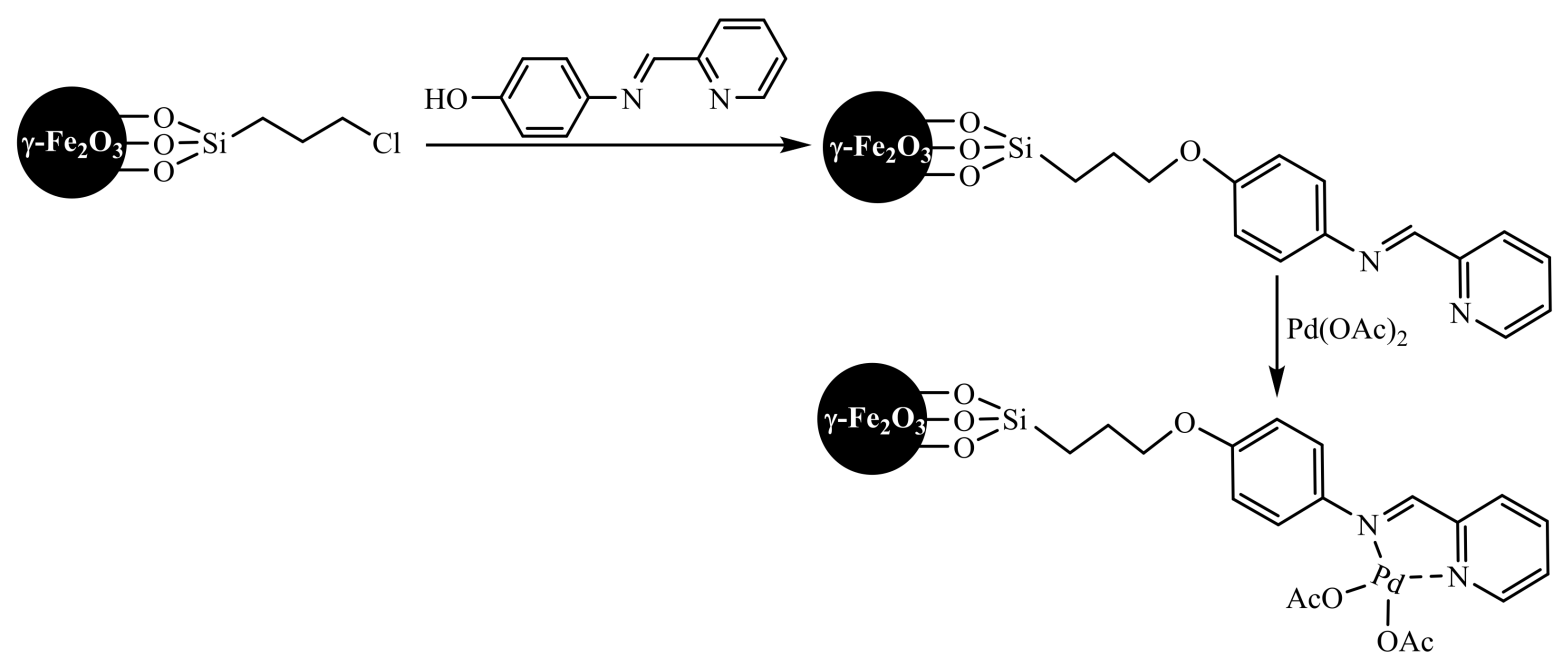
Synthesis of Pd-imino-Py-γ-Fe_2_O_3_.

**Figure 13:**
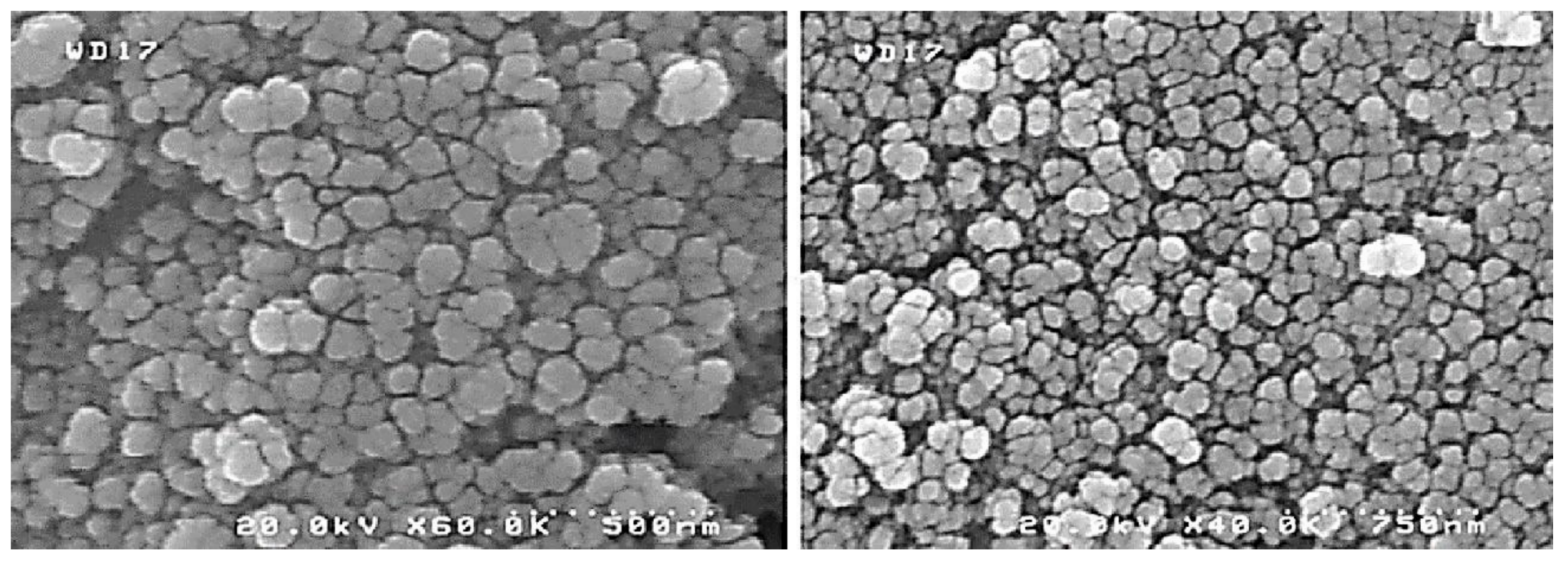

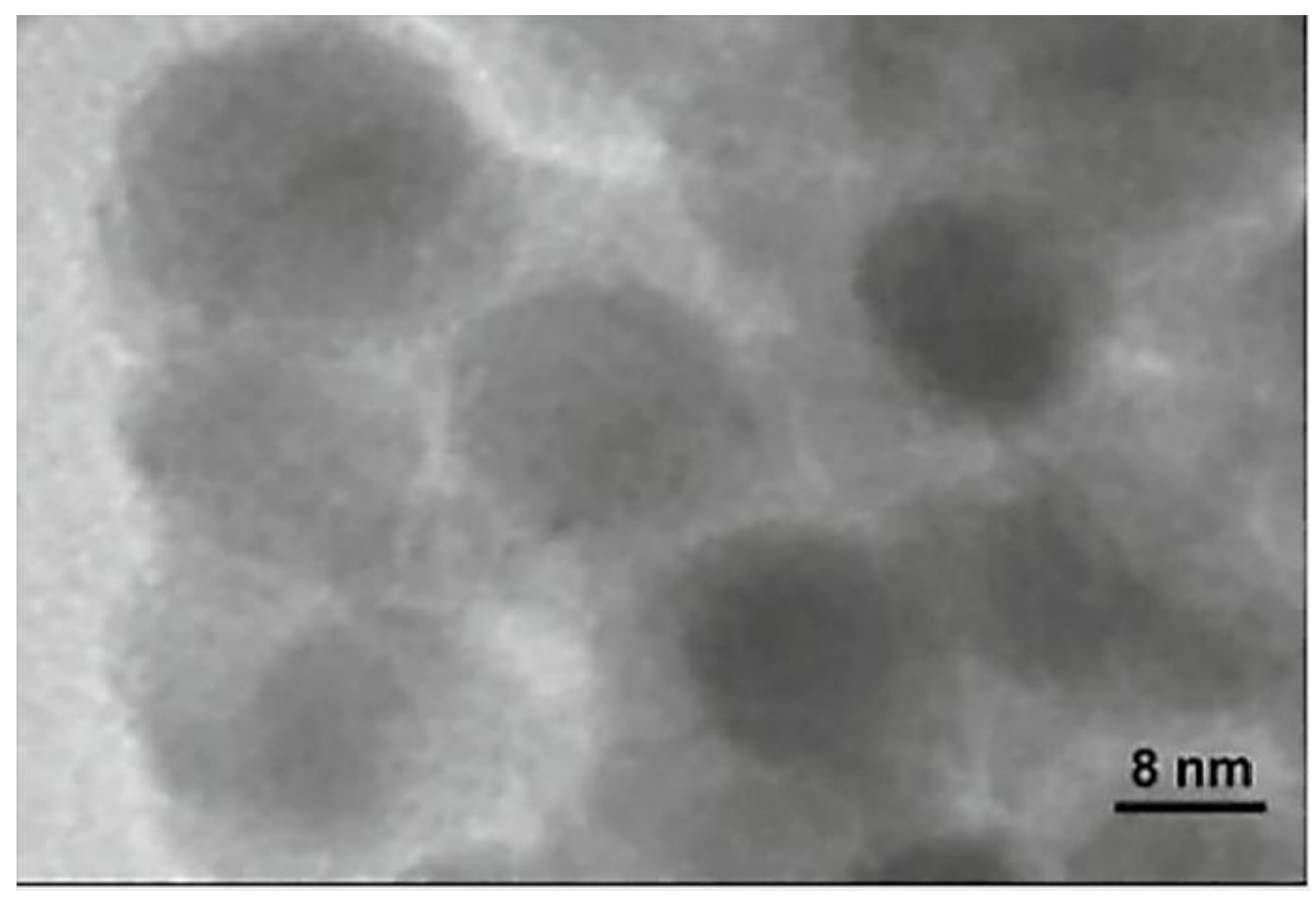
SEM and TEM images of Pd-imino-Py-γ-Fe_2_O_3_. Reproduced with permission from [45].

Sobhani and co-workers reported a palladium‒Schiff base complex covalently immobilized on magnetic NPs for Heck and Suzuki‒Miyaura cross-coupling reactions [45]. Reaction of chloro-functionalized γ-Fe_2_O_3_ with iminopyridine, followed by addition of palladium acetate, led to preparation of the Pd-imino-Py-γ-Fe_2_O_3_ catalyst (Scheme 47). The uniform and spherical morphology of MNPs with an average diameter of about 15 nm was revealed from SEM and TEM images (Figure 13).

**Scheme 48:**
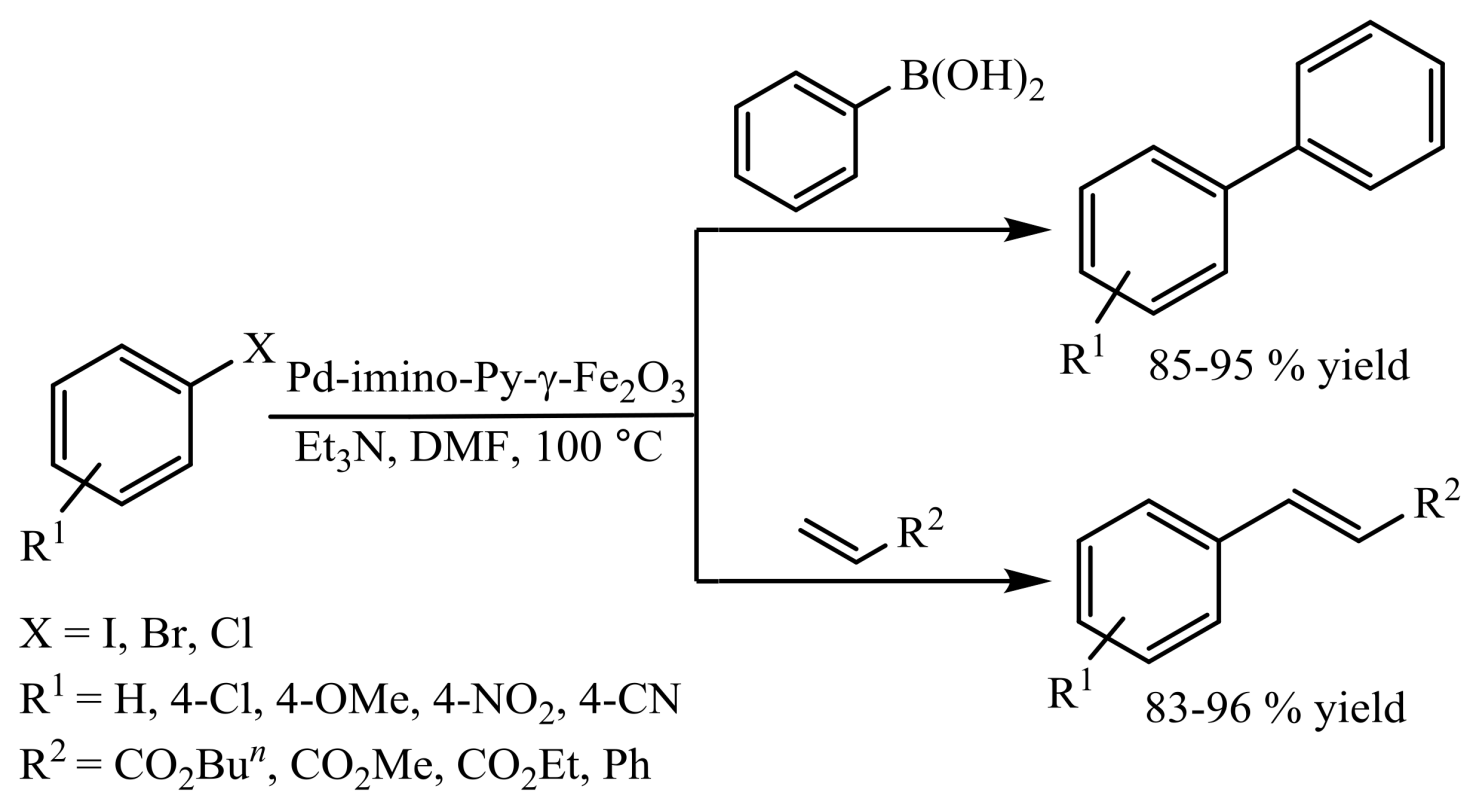
Suzuki‒Miyaura and Heck cross-coupling reactions catalyzed by Pd-imino-Py-γ-Fe_2_O_3_ nanocatalyst [45].

**Figure 14:**
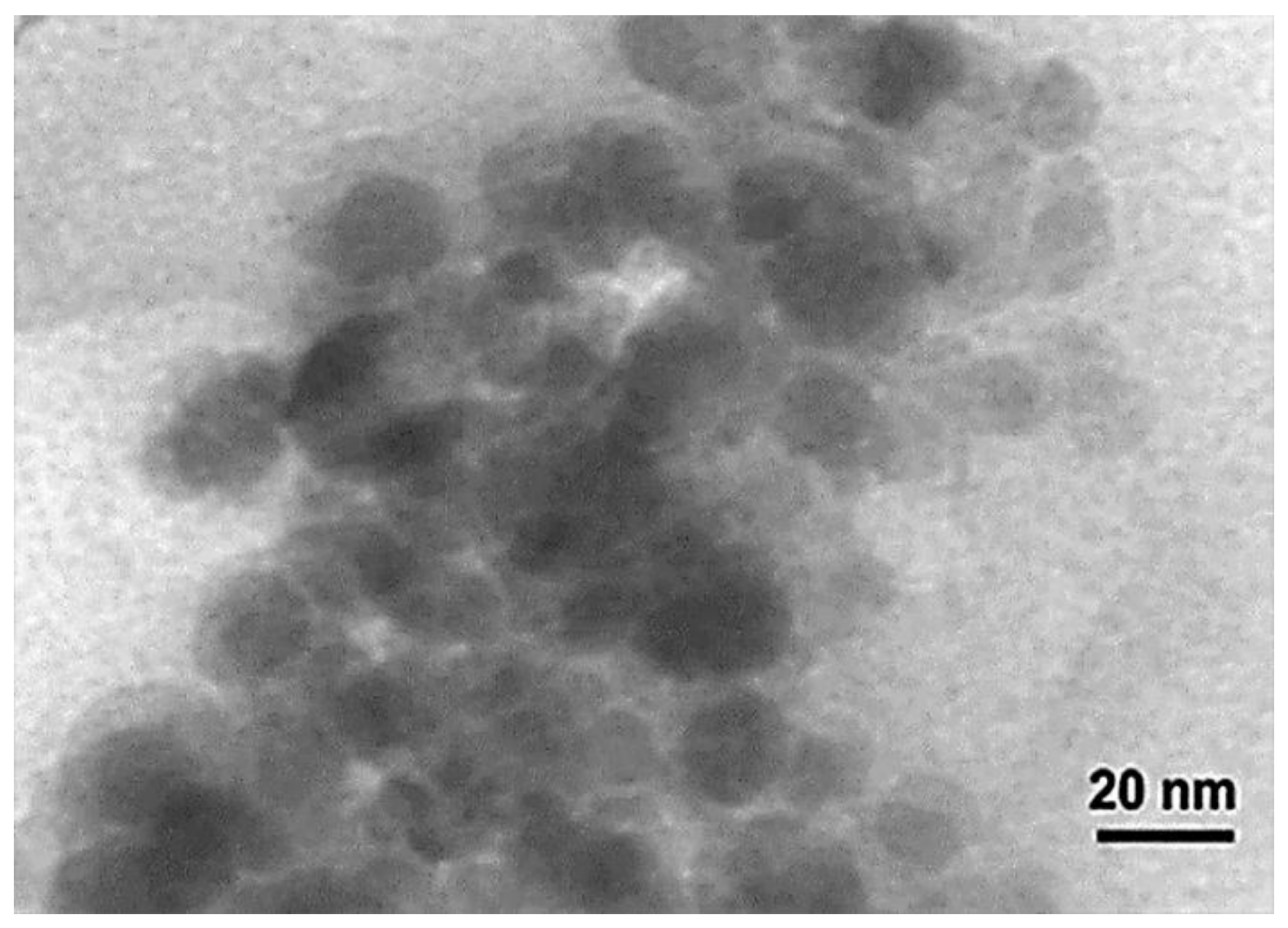
TEM image of Pd-imino-Py-γ-Fe_2_O_3_ after eight reuses. Reproduced with permission from [45].

The heterogenized catalyst was first tested for Heck coupling reaction of various aryl halides with olefins in the presence of Et_3_N as a base in DMF at 100 °C. Furthermore, the catalyst was tested in the Suzuki‒Miyaura coupling reaction of various substituted aryl halides with arylboronic acids under similar conditions as for the Heck reaction (Scheme 48). As magnetic nanoparticles were used as the solid support, the present catalyst was simply separated from the reaction mixture by an external magnet, and reused for at least eight runs without Pd leaching (<1%) and appreciable loss of its catalytic activity. TEM image of used catalyst in Figure 14 showed no morphology change of the catalyst after eight recoveries.

**Scheme 49:**
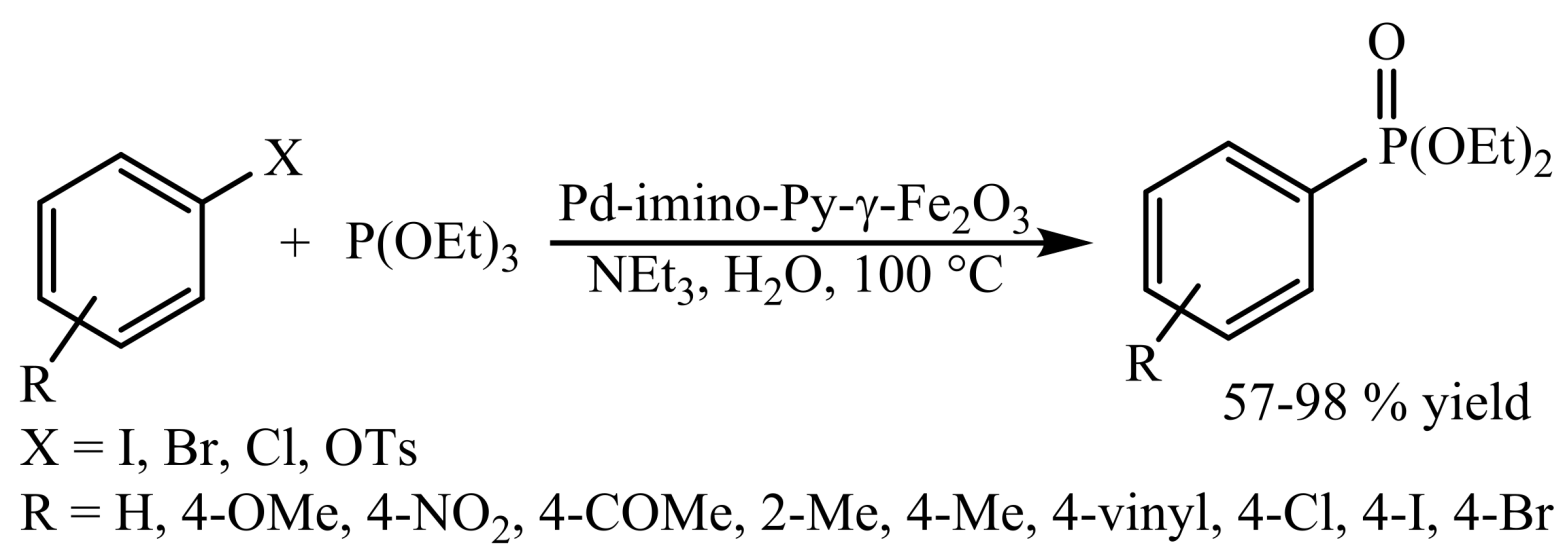
Synthesis of arylphosphonates via C_sp2_-P coupling reaction catalyzed by Pd-imino-Py-γ-Fe_2_O_3_.

In a separate study, the Pd-imino-Py-γ-Fe_2_O_3_ nanocatalyst was also employed for the synthesis of arylphosphonates via a C_sp2_-P coupling reaction in water, affording the coupling products in high yields [46]. The recyclability of the catalyst was investigated in the reaction of iodobenzene and triethylphosphite. After completion of the reaction, the catalyst could simply be recovered from the reaction mixture with a magnetic bar; after washing with EtOAc and drying, it was applied for the next reaction and showed good yield for eight runs. ICP analysis of the catalyst after eight runs showed that less than 1% of Pd metal was removed from the catalyst (Scheme 49).

**Scheme 50:**
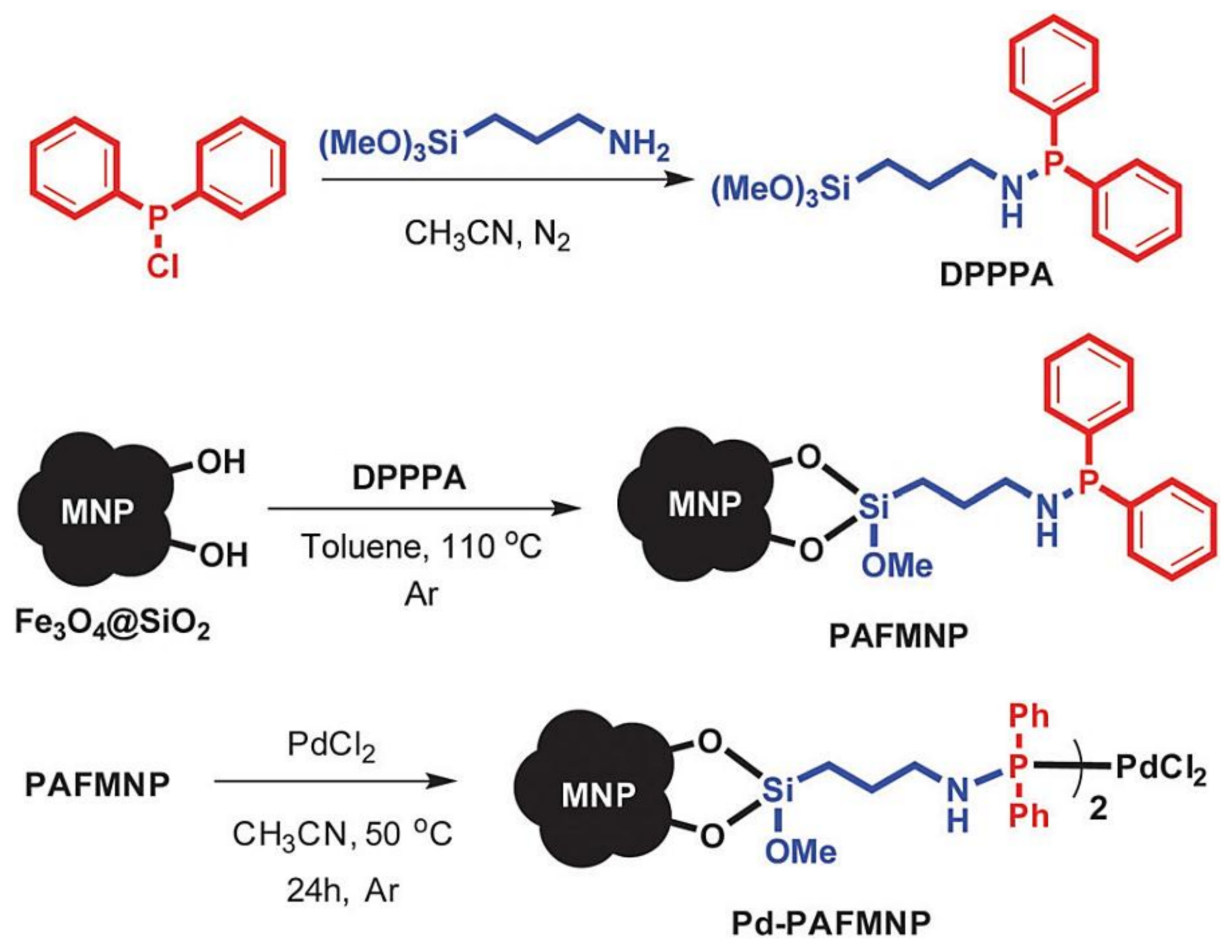
Synthesis of the Pd-PAFMNP catalyst. Reproduced with permission from [47].

**Figure 15:**
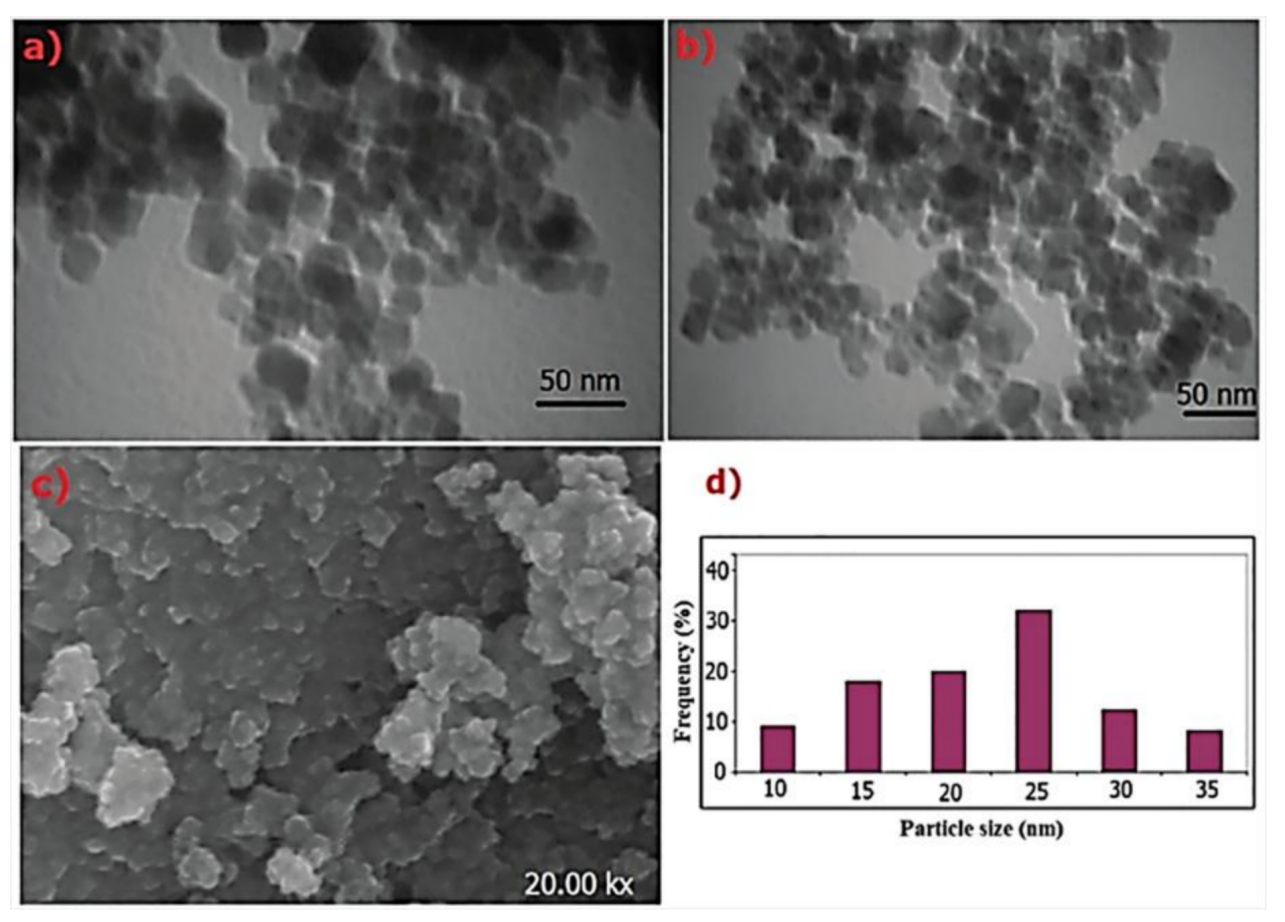
(**a**,**b**) TEM images of the PAFMNP ligand; (**c**) an SEM image of the PAFMNP ligand. (**d**) A histogram representing the size distribution of the PAFMNP NPs. Reproduced with permission from [47].

Panahi et al. reported the synthesis of a new magnetic reusable phosphorous ligand for the preparation of phosphanamine-functionalized magnetic NPs (PAFMNP) [47]. PAFMNP was obtained by reaction of phosphanamine-functionalized trimethoxysilyl compound (DPPPA) with magnetic NPs. The obtained material reacted with palladium chloride, leading to an efficient magnetic recyclable Pd catalyst (Scheme 50). TEM images of the Pd-PAFMNP catalyst showed nearly spherical NPs with an average particle size of 25 nm and good monodispersity (Figure 15).

**Scheme 51:**
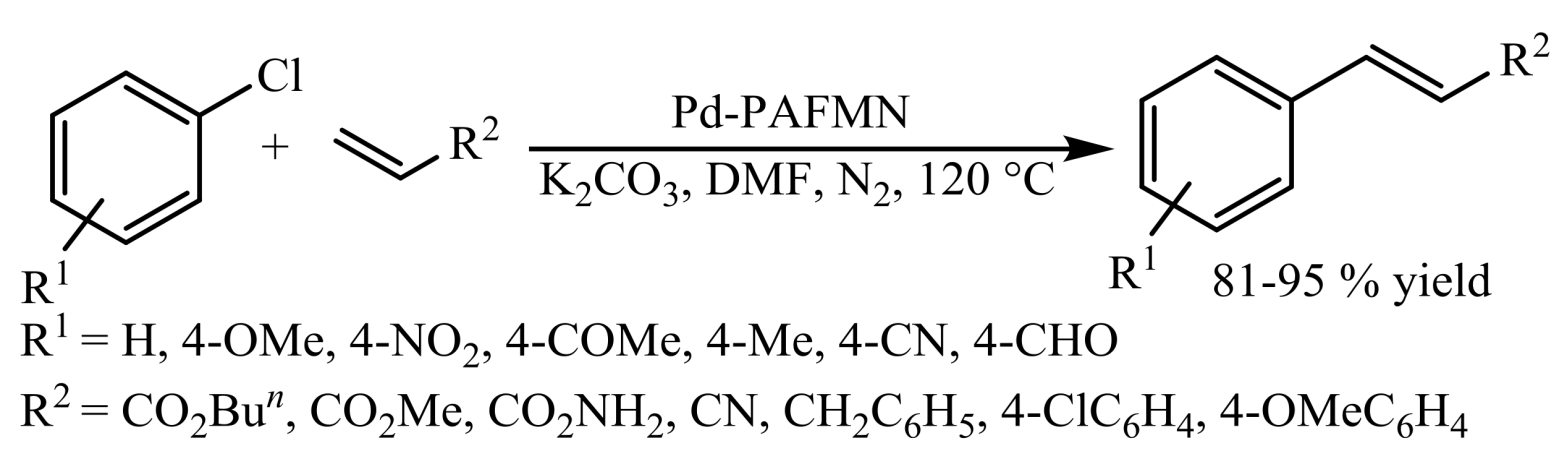
Heck reaction catalyzed by Pd-PAFMNP catalyst [47].

Pd-PAFMNP acts as an efficient magnetic recyclable catalyst for the Heck reaction of chloroarenes as inactive substrates in the presence of only 1.0 mol % of catalyst (Scheme 51). The catalyst with magnetic nature could be recycled and reused at least five times without a significant loss of activity. ICP results after fifth runs showed that only 0.5% of the Pd was lost.

**Scheme 52:**
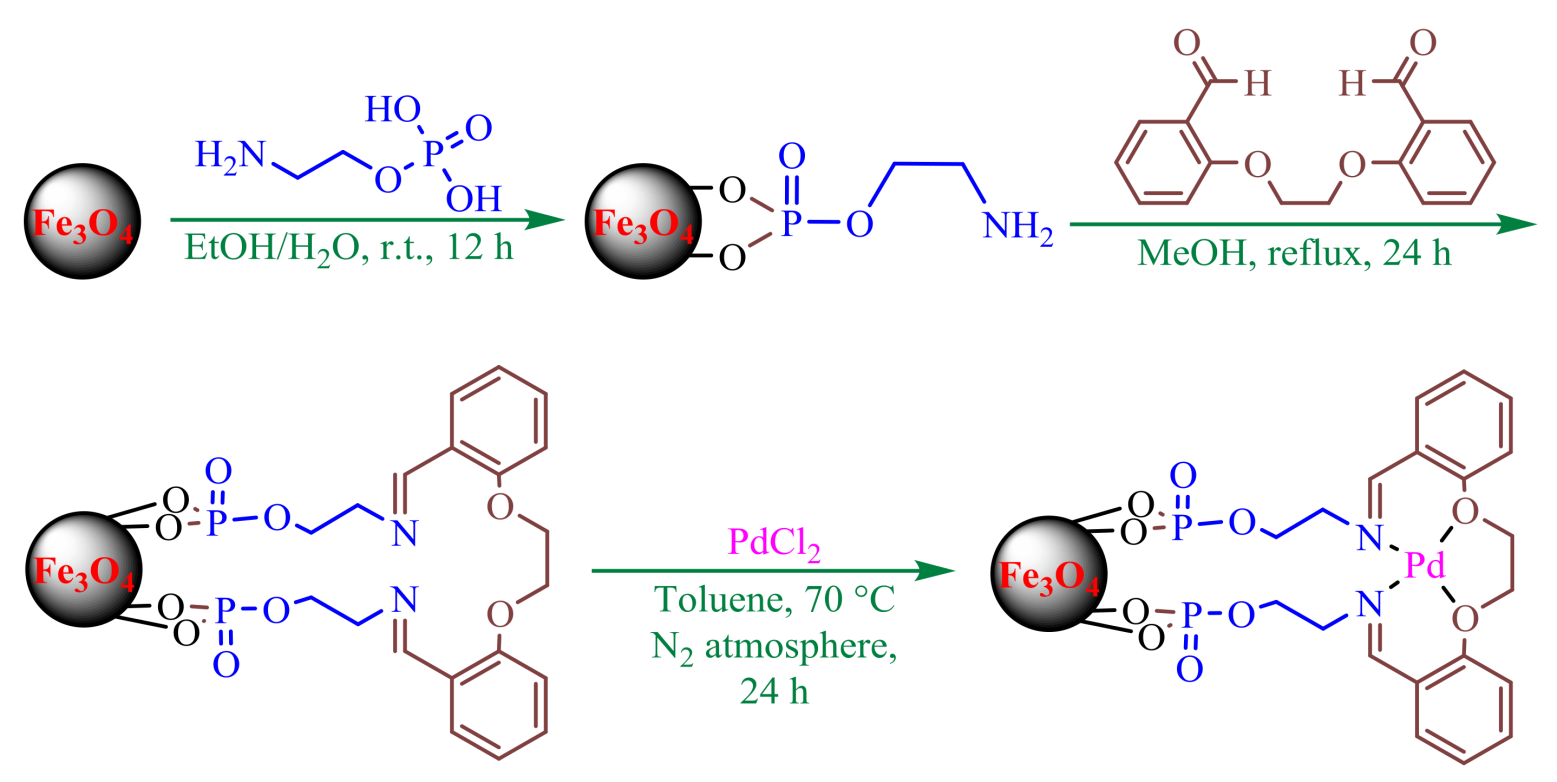
Preparation of novel task-specific nano-magnetic Schiff base ligand with phosphate spacer and its Pd nano-magnetic heterogeneous catalyst [48].

**Figure 16:**
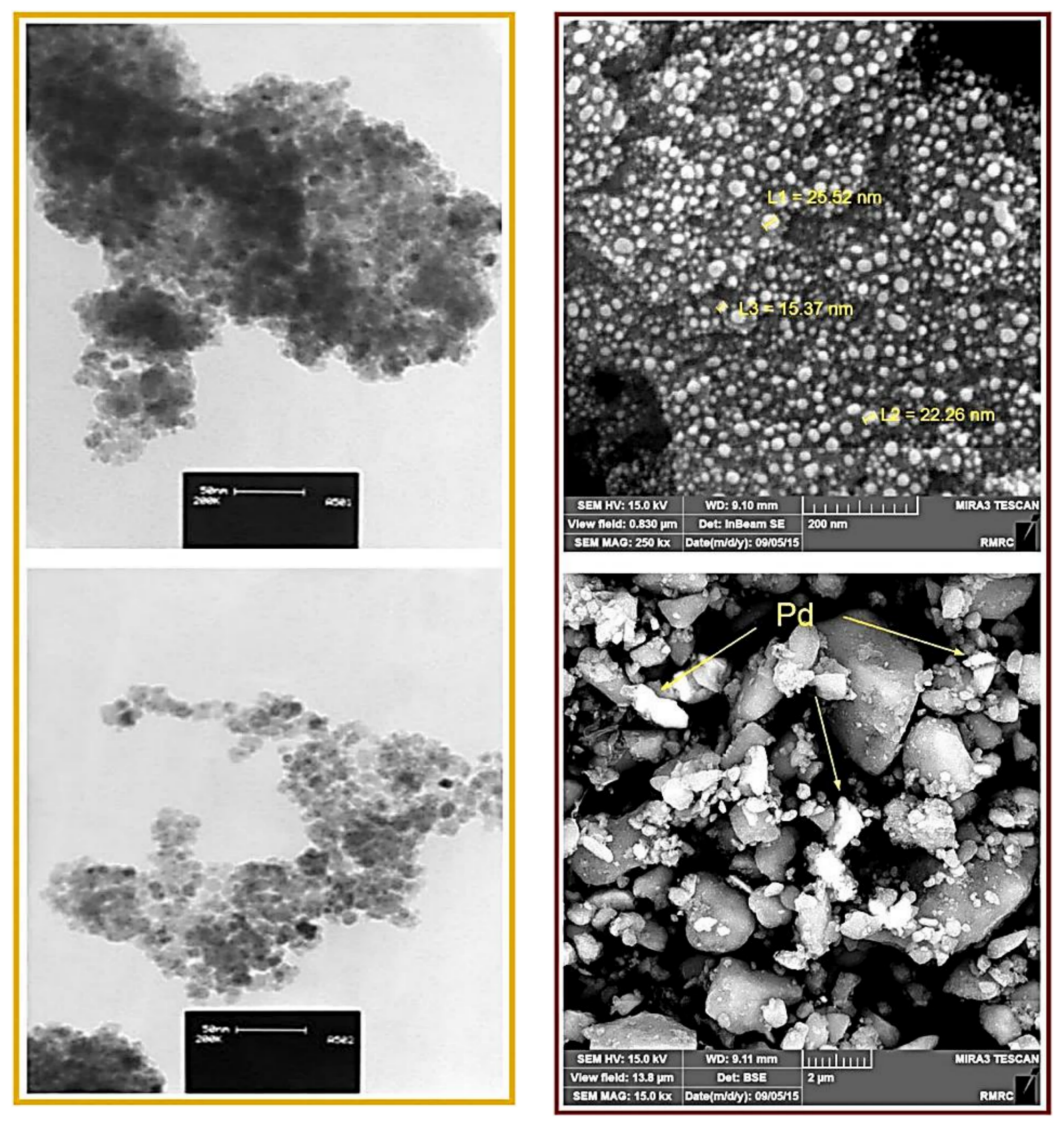
TEM and SEM images of the magnetic nano-palladium Schiff base complex. Reproduced with permission from [48].

Zolfigol and co-workers described the formation of a novel magnetic nano-palladium complex with task-specific nano-magnetic Schiff base ligand, wherein 2-aminoethyl dihydrogen phosphate instead of the usual coating agents for coating of nano-magnetic Fe_3_O_4_ was introduced (Scheme 52) [48]. The Pd-based nano-magnetic catalyst consisted of particles of less than 50 nm (Figure 16).

**Scheme 53:**
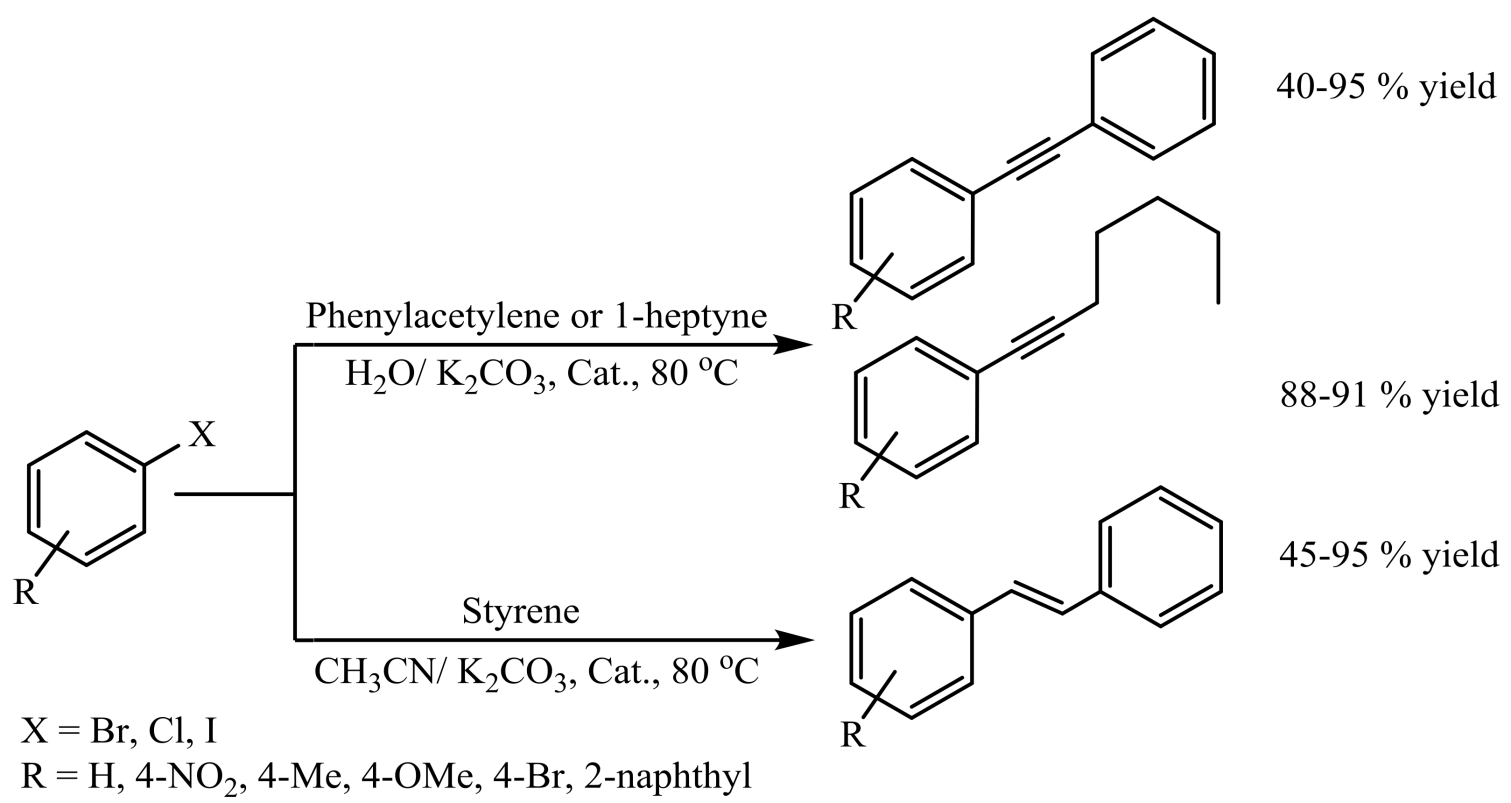
Applicability of the magnetic nano-palladium Schiff base complex in C‒C coupling reactions [48].

The resulting catalyst showed high activity in the Sonogashira and Heck C‒C coupling reactions (Scheme 53). The catalyst was recycled nine times without any significant loss of its initial catalytic activity, confirming the high stability of the prepared catalyst.

**Figure 17:**
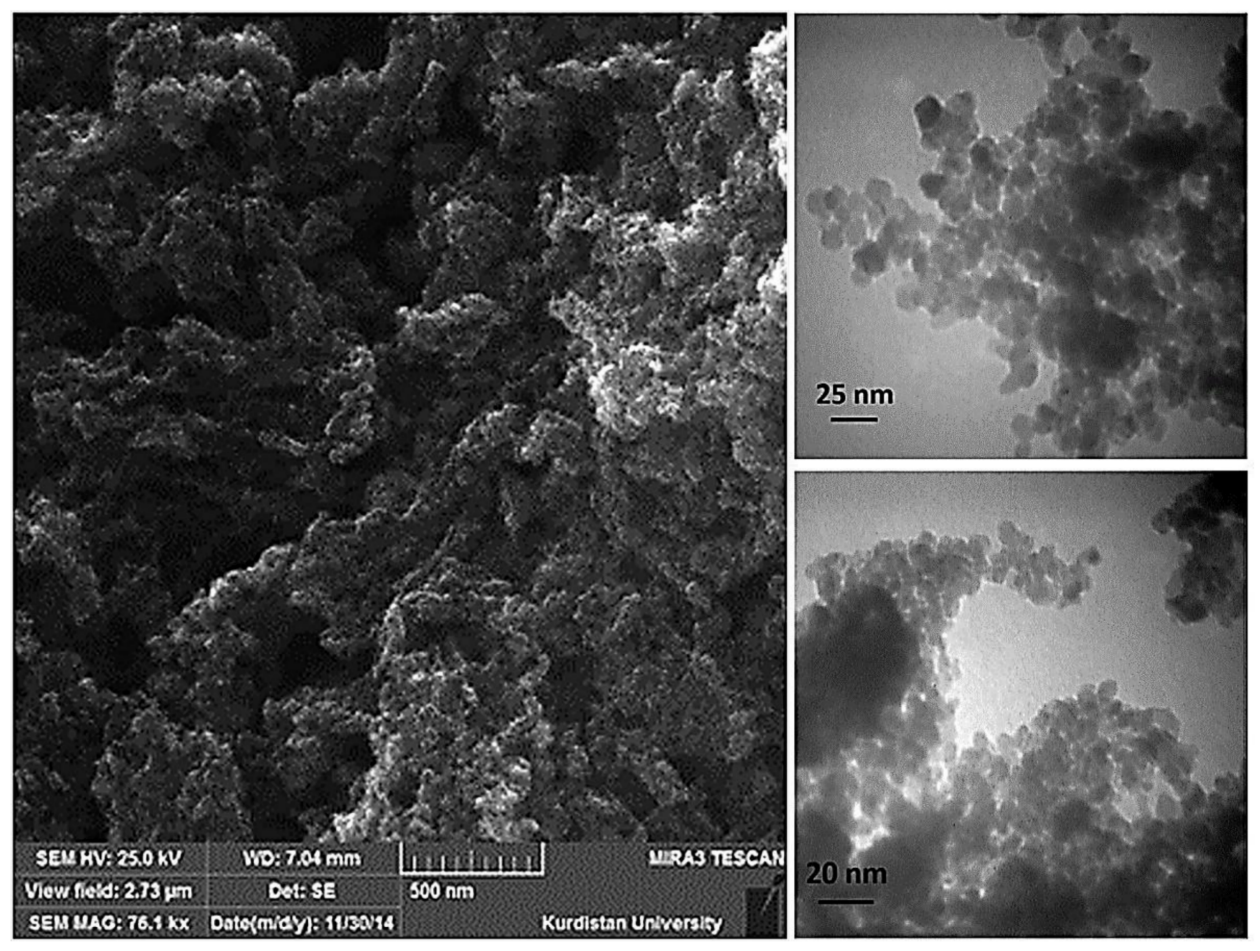
SEM and TEM images of Schiff-base-Pd@MNPs. Reproduced with permission from [49].

Ghorbani-Choghamarani and co-workers reported the Schiff base complex of palladium immobilized on magnetic NPs. Fe_3_O_4_ NPs were synthesized through a co-precipitation procedure: the coating of Fe_3_O_4_ NPs with APTES followed by the reaction of 5-bromosalicylaldehyde with amino groups led to a 5-bromosalicylaldehyde Schiff base supported on Fe_3_O_4_ NPs (Schiff-base@MNPs) for the formation of a complex with palladium. The resulting Schiff-base-Pd@MNPs catalyst was composed of quite homogeneous and quasi-spherical particles with an average diameter of about 10 nm (Figure 17) [49].

**Scheme 54:**
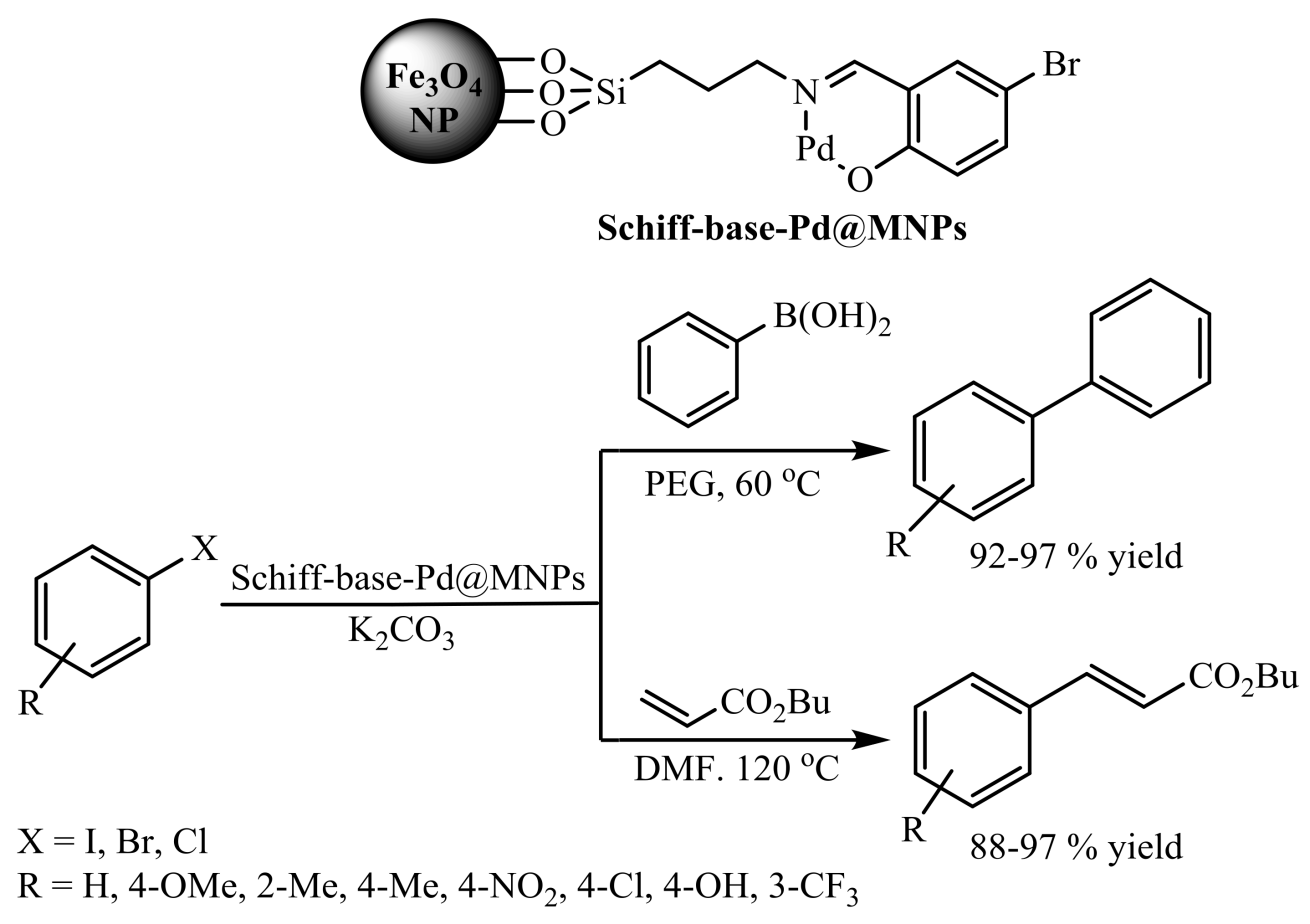
Schiff-base-Pd@MNPs catalyzed C‒C coupling through Suzuki‒Miyaura and Heck reactions [49].

The Schiff-base-Pd@MNPs acted as recyclable nanocatalyst for C‒C bond formation through Heck and Suzuki‒Miyaura reactions. The Suzuki‒Miyaura reaction of various aryl halides and phenylboronic acid was studied in the presence of K_2_CO_3_ in poly(ethylene glycol) (PEG)-400 at 60 °C to furnish diverse biaryl products in yields of 92–97%. Also, coupling aryl halides with butyl acrylate through Heck reaction was conducted in the presence of K_2_CO_3_ in DMF at 120 °C and pure products were obtained in 88‒97% yields (Scheme 54). In addition, this catalyst could be separated from the reaction mixture by employing a magnetic bar, followed by washing with diethyl ether, and could be reused for up to six runs without any significant loss of its activity or palladium leaching with average isolated yield for six runs in 93.5%.

**Scheme 55:**
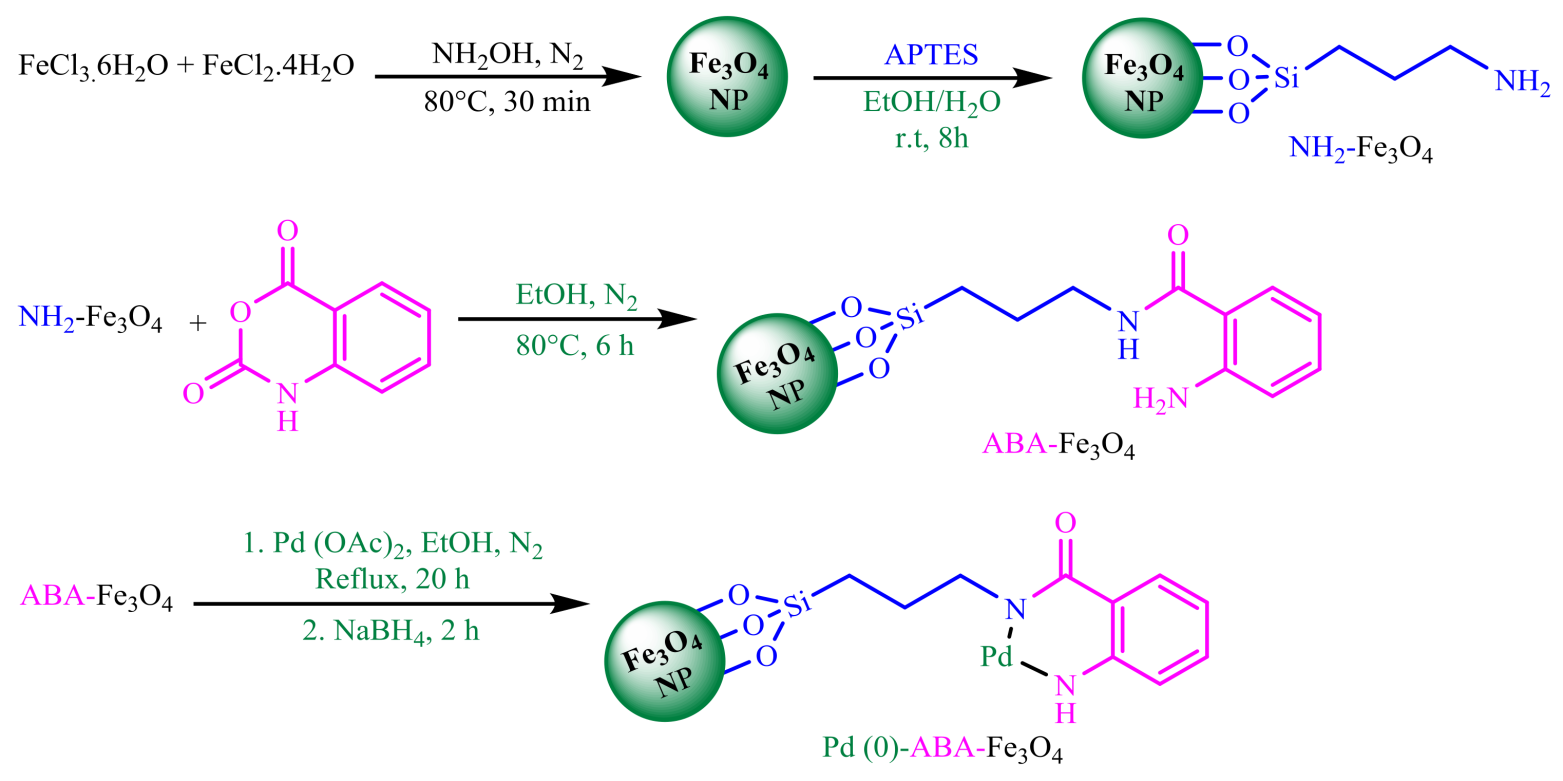
Synthesis of Pd(0)-ABA-Fe_3_O_4_ NPs. Reproduced with permission from [50].

**Figure 18:**
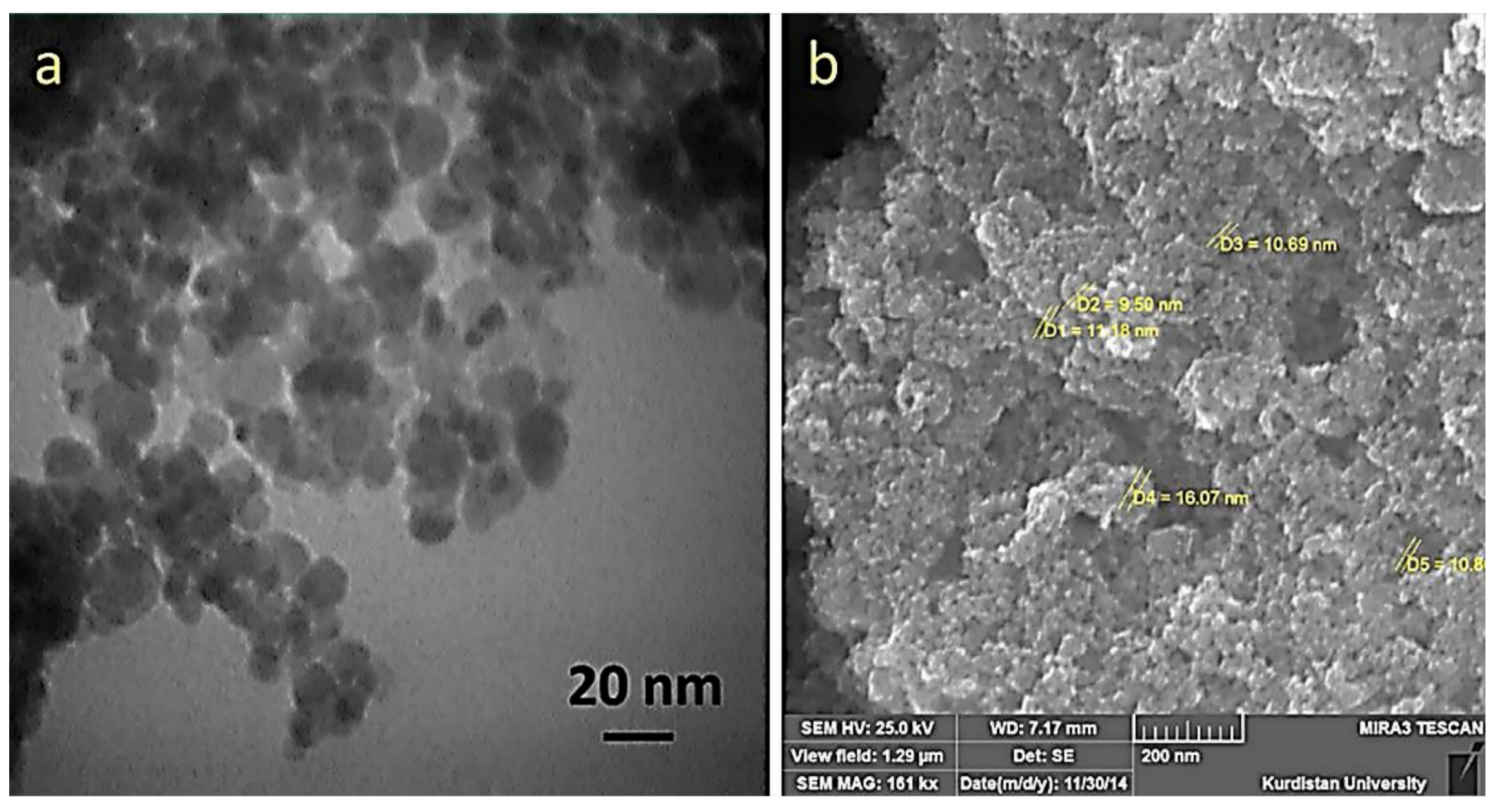
TEM (**a**) and SEM (**b**) image of Pd(0)-ABA-Fe_3_O_4_. Reproduced with permission from [50].

In another study, the same authors synthesized a catalyst for amination of aryl halides, Heck and Suzuki‒Miyaura reactions based on palladium supported on magnetite (Pd(0)-ABA-Fe_3_O_4_) by pretreatment of the magnetite particles with (3-aminopropyl) trimethoxysilane (APTES). By immobilizing isatoicanhydride to the surface of amino-functionalized magnetic NPs fixed ligand system was obtained for coordination with palladium(II) (Scheme 55) [50]. A TEM image of the obtained catalyst shows spherical and quite homogeneous NPs with an average size of about 15–25 nm (Figure 18).

**Scheme 56:**
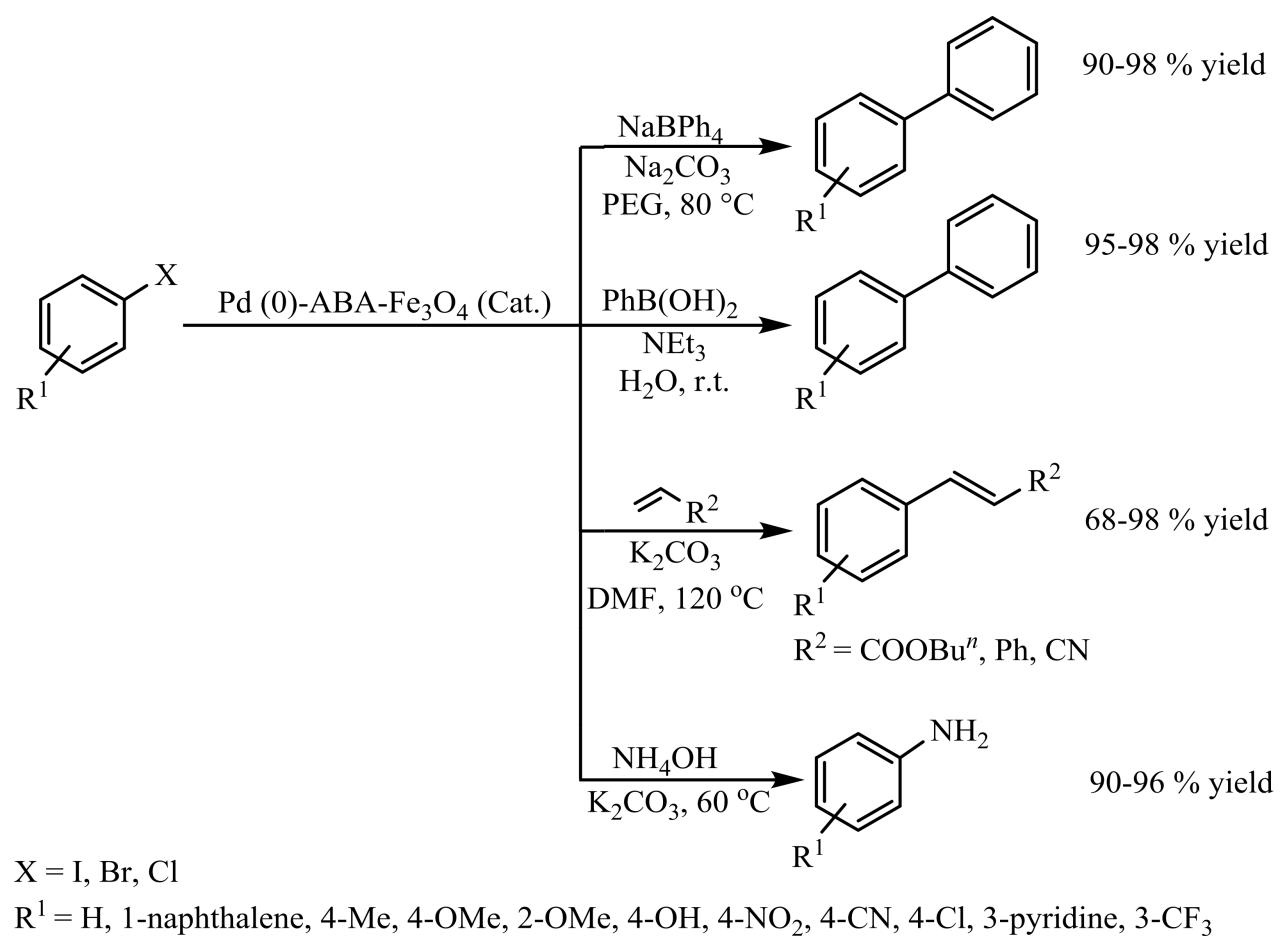
Pd(0)-ABA-Fe_3_O_4_ catalyzed Heck and Suzuki‒Miyaura reactions [50].

The Pd(0)-ABA-Fe_3_O_4_ catalyst was applied for the amination of aryl halides, Heck and Suzuki‒Miyaura reactions. The Suzuki‒Miyaura reaction was carried out in water or PEG using phenylboronic acid (PhB(OH)_2_) or sodium tetraphenyl borate (NaBPh_4_). Also Pd(0)-ABA-Fe_3_O_4_ has been used for Heck reaction of butyl acrylate, styrene or acrylonitrile with aryl halides in the presence of Na_2_CO_3_ as the base in DMF at 120 °C (Scheme 56). Recycling experiments conducted for the coupling reaction of 4-nitrobromobenzene with phenylboronic acid and sodium tetraphenyl borate, after completion the reaction, the catalyst was easily separated using an external magnet washed with diethyl ether and was used over five runs without any significant loss of its activity.

**Scheme 57:**
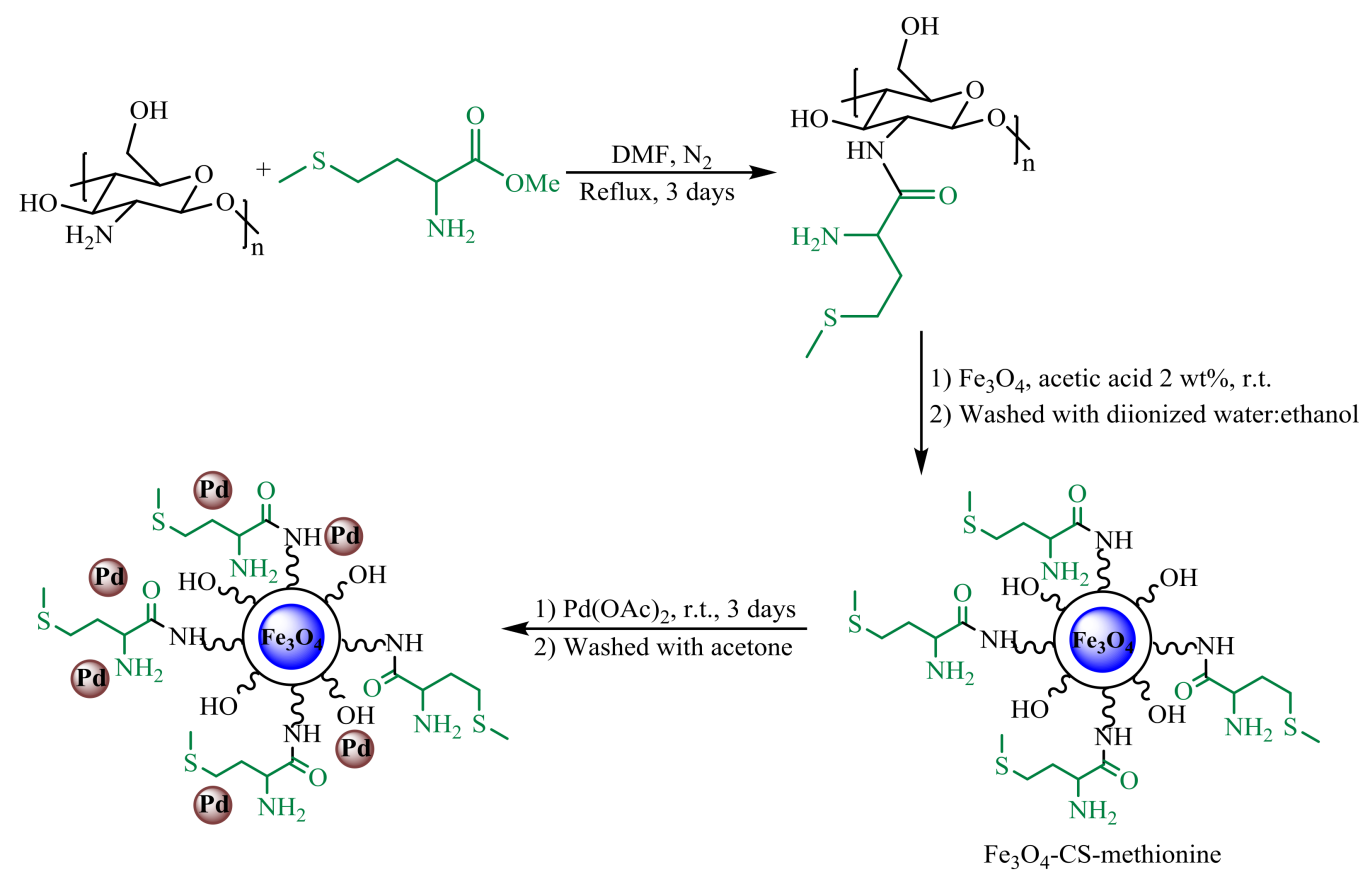
Synthetic paths for the preparation of ImmPd‒MNPs catalyst [51].

**Figure 19:**
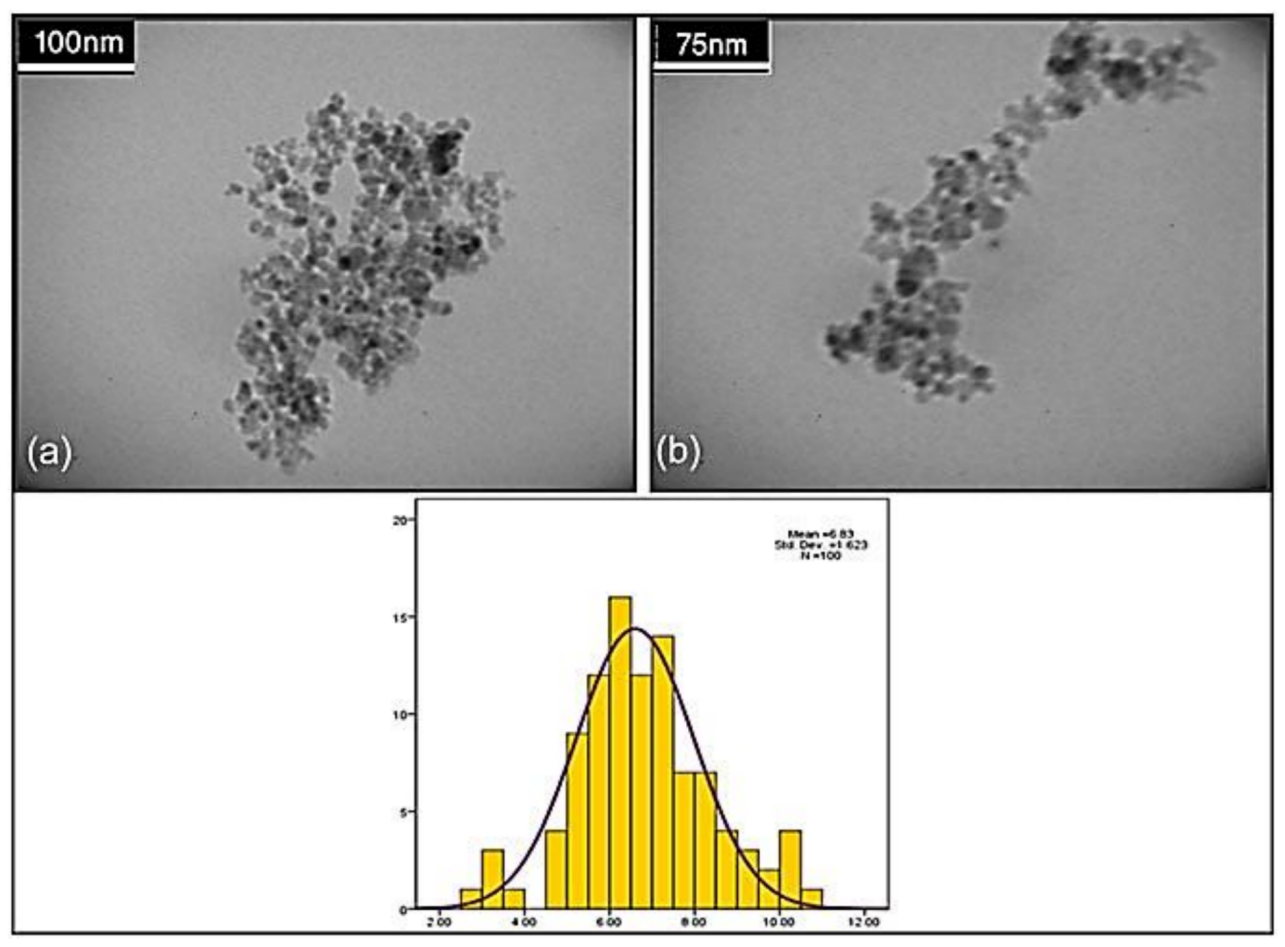
(**a**) TEM image of ImmPd(0)‒MNPs catalyst showing the morphology of Pd nanoparticles on the magnetic chitosan support. Beneath is the histogram of the particle size distribution. (**b**) TEM image of ImmPd(0)‒MNPs catalyst after eight reuses. Reproduced with permission from [51].

Hajipour and co-workers reported the preparation of a methionine-functionalized chitosan‒Pd(0) complex. The magnetic catalyst was obtained by functionalization of chitosan with methionine, followed by treatment with Fe_3_O_4_ NPs; finally, Fe_3_O_4_-CS-methionine reacted with palladium acetate and led to high palladium loadings (Scheme 57) [51]. A TEM image of the catalyst (ImmPd-MNPs) showed near-spherical Pd NPs that accumulated onto the magnetic functionalized chitosan with good monodispersity (Figure 19).

**Scheme 58:**
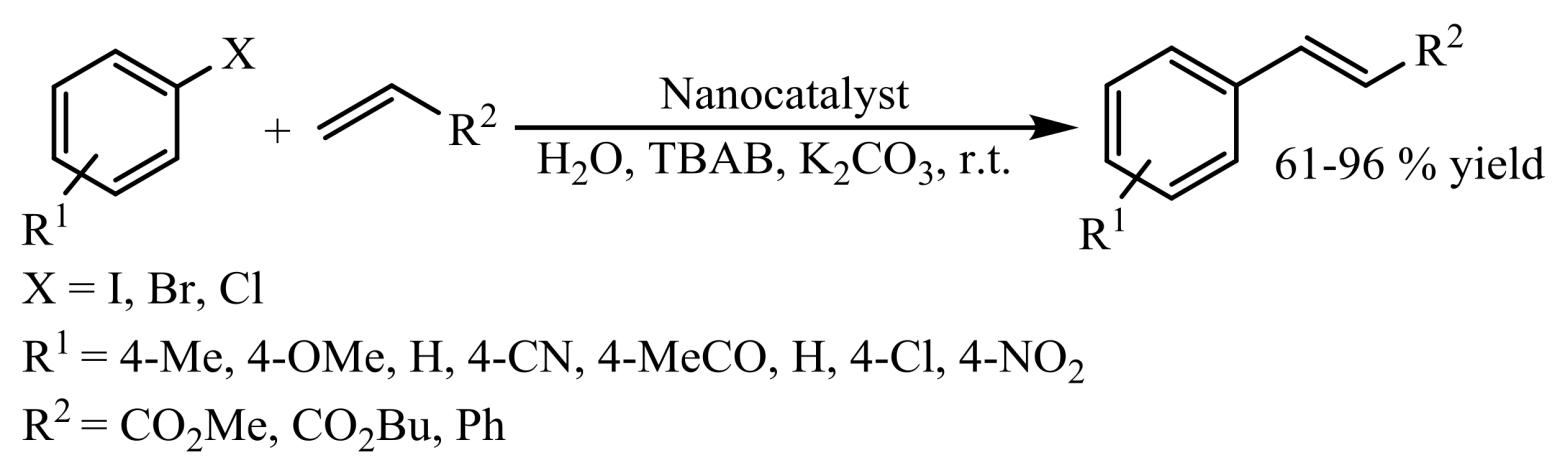
ImmPd(0)-MNPs-catalyzed Heck reaction between various aryl halides and olefins [51].

The ImmPd‒MNPs catalyst was found to be active for Heck reactions of aryl iodides and aryl bromides under green reaction conditions. A high yield of alkene derivatives was achieved in the presence of 0.21 mol% of nanocatalyst using water as the solvent, K_2_CO_3_ as the base and the phase-transfer reagent TBAB (Scheme 58). The recyclability of the catalyst was evaluated with six consecutive coupling reactions without loss of its activity.

**Scheme 59:**
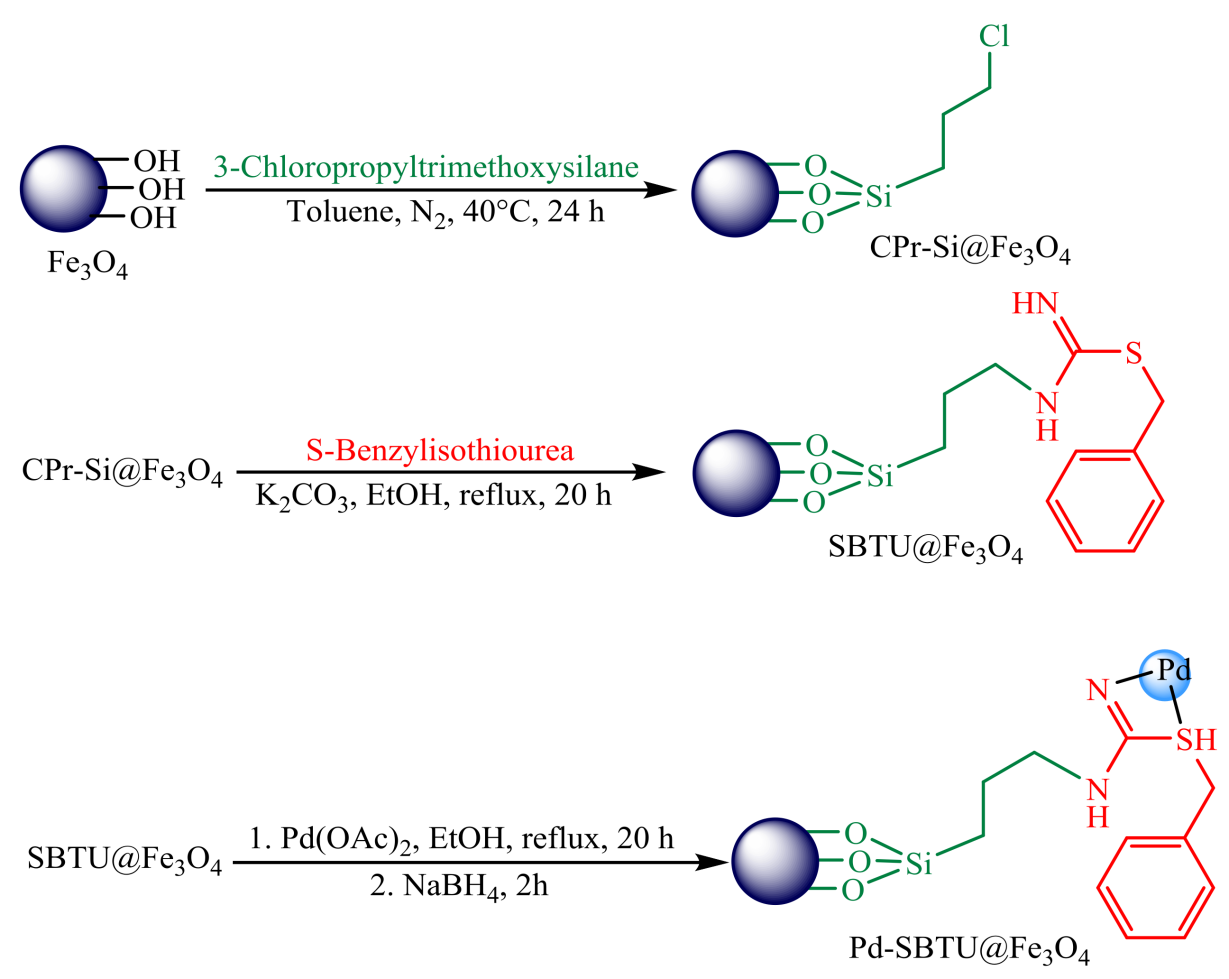
Synthesis of Pd-SBTU@Fe_3_O_4_. Reproduced with permission from [52].

**Figure 20:**
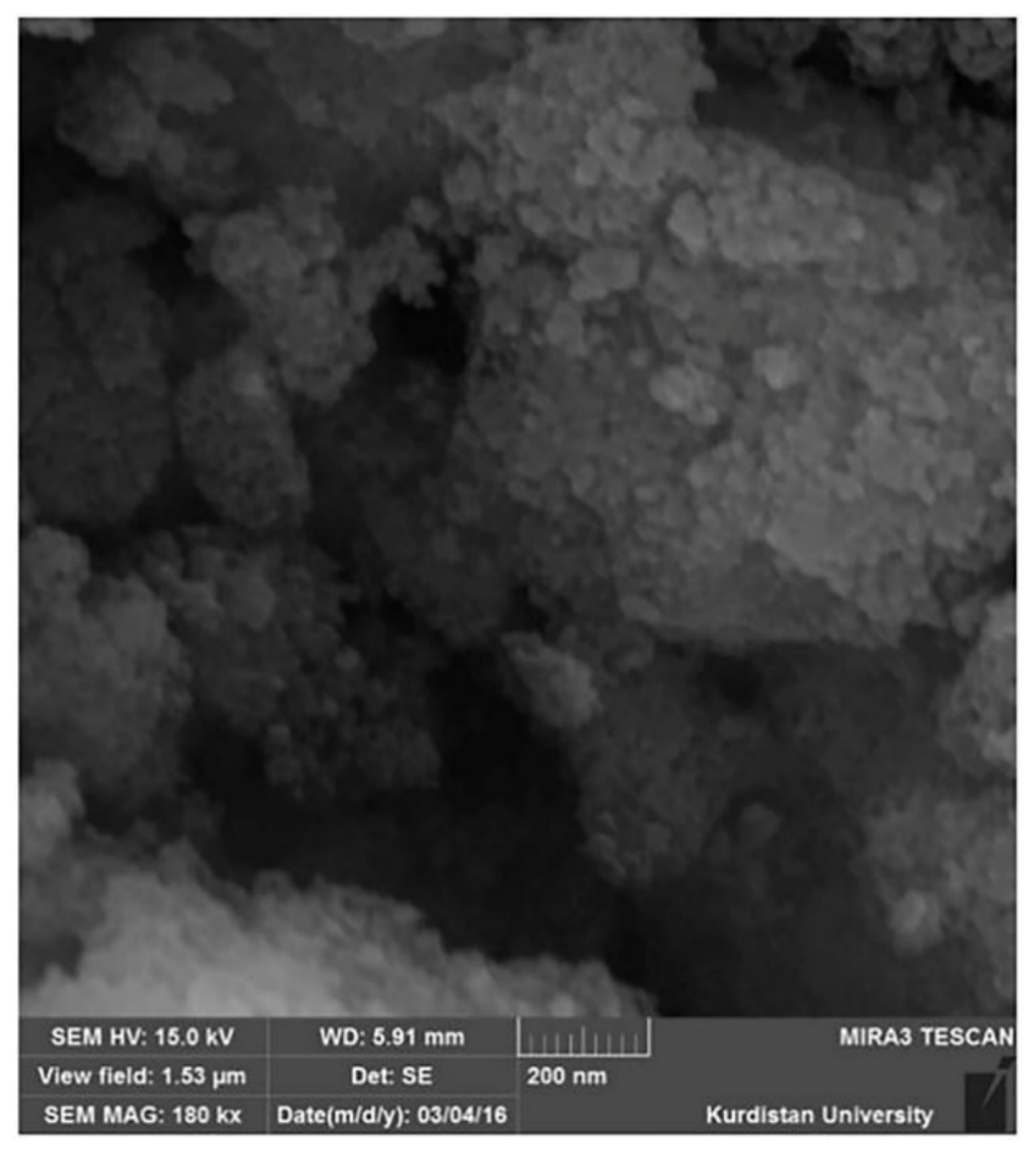
SEM image of Pd-SBTU@Fe_3_O_4_. Reproduced with permission from [52].

Ghorbani-Choghamarani and co-workers published a moisture- and air-stable palladium complex supported on Fe_3_O_4_ magnetic NPs (Pd-SBTU@Fe_3_O_4_). Modification of magnetic core with 3-chloropropyltrimethoxysilane followed by addition of *S*-benzylisothiourea led to a covalently fixed ligand system that was coordinated to palladium(II) (Scheme 59) [52]. The SEM image of Pd-SBTU@Fe_3_O_4_ showed quasi-spherical particles with an average diameter of about 20 ± 5 nm (Figure 20).

**Scheme 60:**
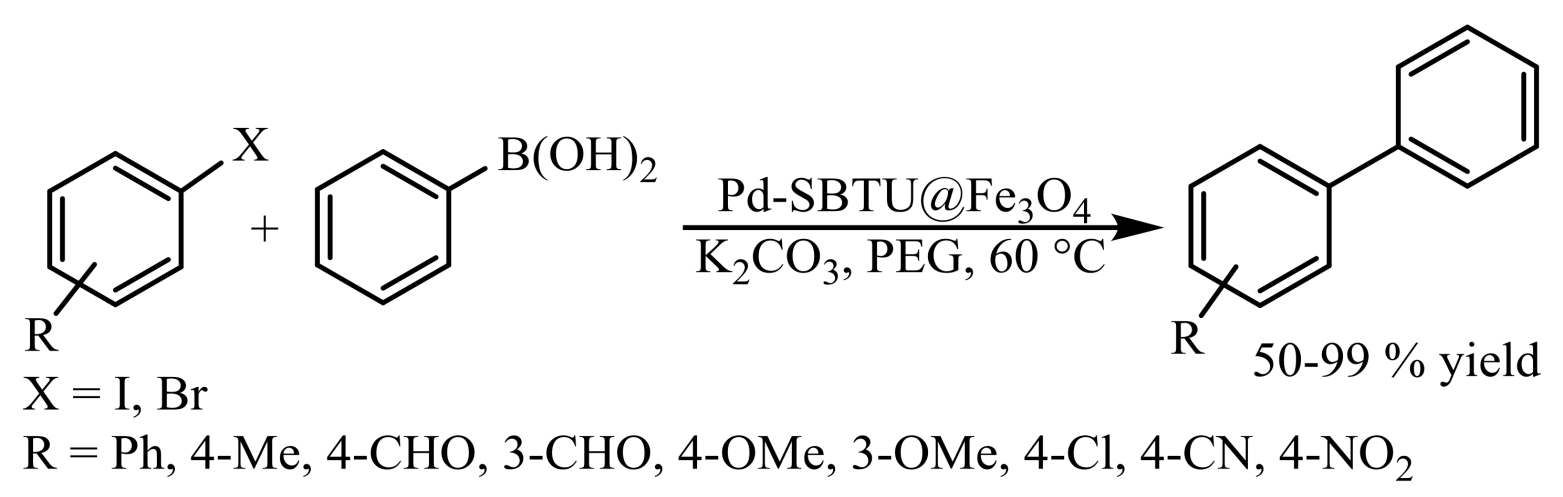
Pd-SBTU@Fe_3_O_4_-catalysed Suzuki‒Miyaura reaction [52].

The catalyst Pd-SBTU@Fe_3_O_4_ performed as an organometallic reusable nanocatalyst in the synthesis of polyhydroquinolines and Suzuki‒Miyaura coupling reaction. In the presence of this catalyst, the best results for Suzuki‒Miyaura cross-coupling of different aryl halides with phenylboronic acid were obtained with K_2_CO_3_ as the base in PEG-400 as a green solvent at 60 °C (Scheme 60). Furthermore, the catalyst could be easily recovered using an external magnet and reused five times without any significant loss of its activity.

**Scheme 61:**
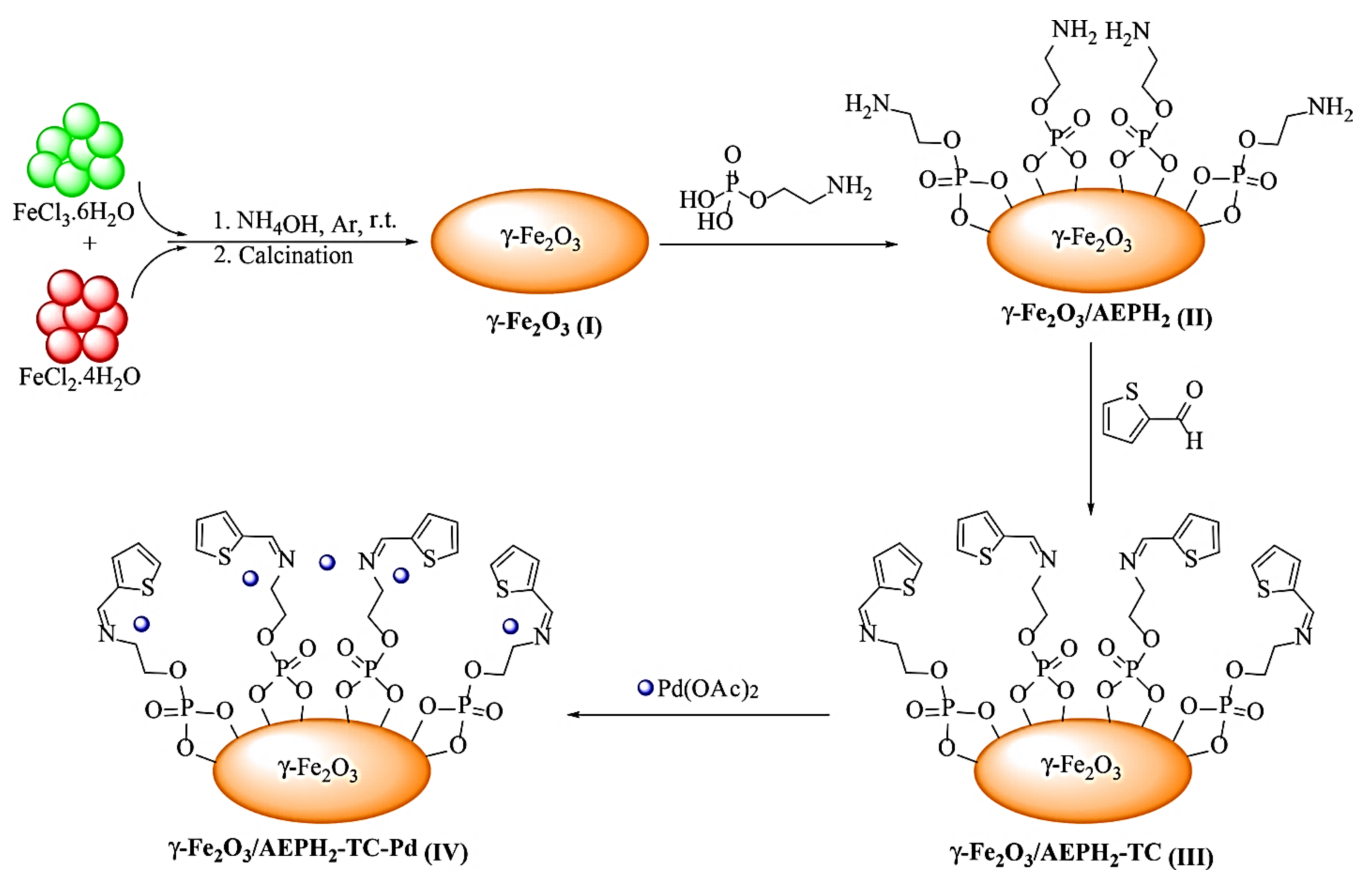
An overview on the synthesis of γ-Fe_2_O_3_/AEPH_2_-TC-Pd nanocatalyst. Reproduced with permission from [53].

**Figure 21:**
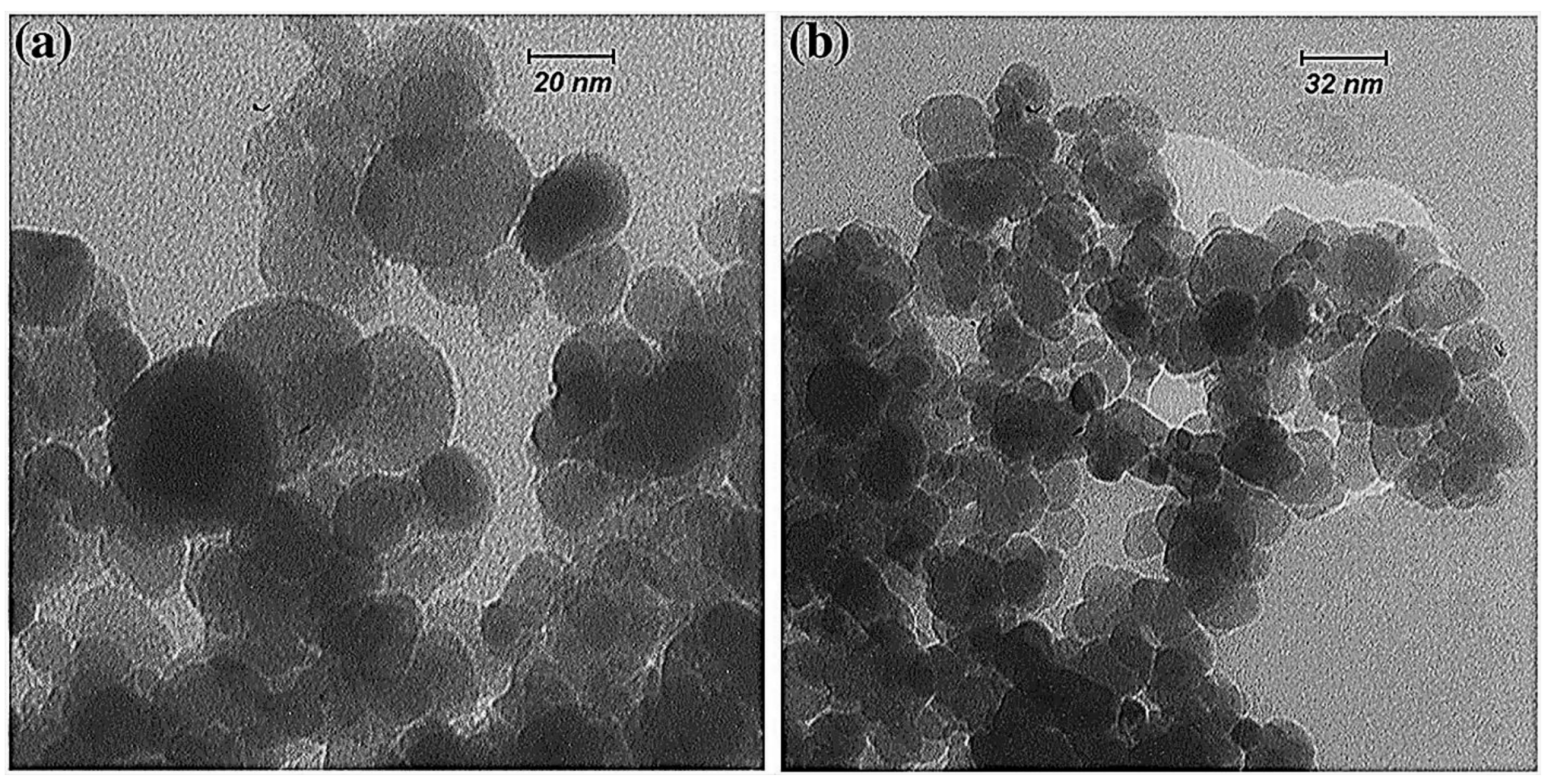
TEM images of γ-Fe_2_O_3_/AEPH_2_-TC-Pd. Reproduced with permission from [53].

Akhlaghinia and coworker described the formation of a magnetic organometallic catalyst wherein a thiophene methanimine-palladium Schiff base complex was immobilized on decorated γ-Fe_2_O_3_ with 2-aminoethyl dihydrogen phosphate (γ-Fe_2_O_3_/AEPH_2_-TC-Pd) (Scheme 61) [53]. This nanocomposite has spherical morphology with an average diameter of about 15 nm (Figure 21).

**Scheme 62:**
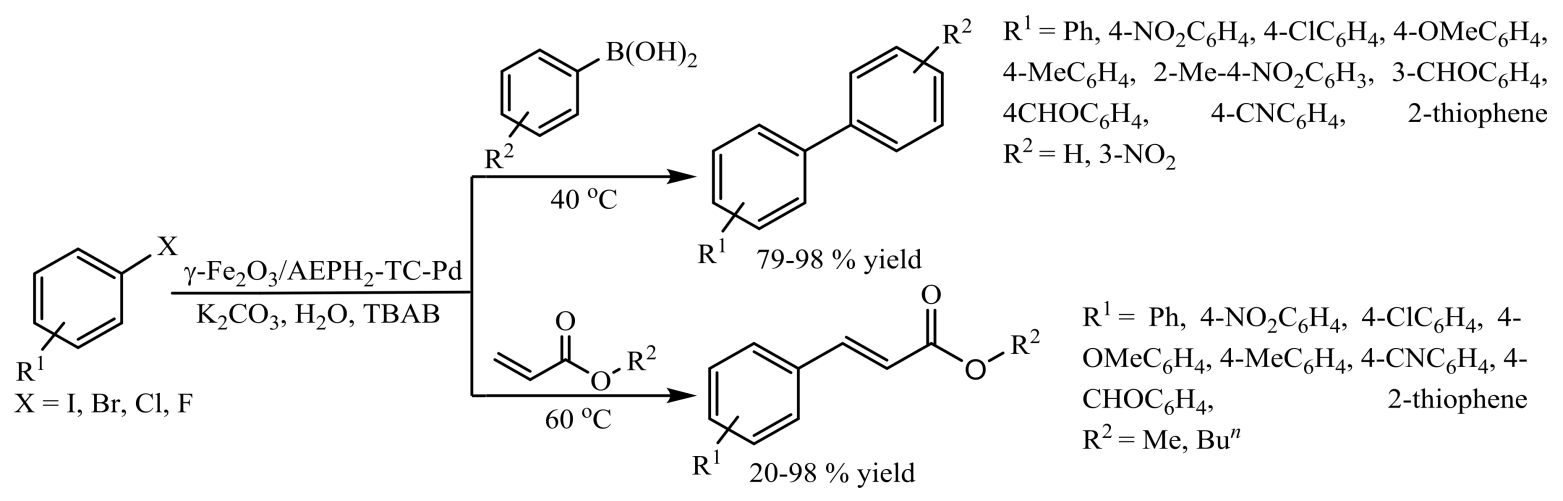
Suzuki‒Miyaura and Heck cross-coupling reactions in the presence of γ-Fe_2_O_3_/AEPH_2_-TC-Pd nanocatalyst [53].

The catalyst exhibited high activities for Suzuki‒Miyaura and Heck cross-coupling reactions in aqueous media (Scheme 62). Also, this catalyst can be separated from the reaction mixture by using a magnetic bar and then washing it with ethanol, and can subsequently be reused at least nine times without a significant decrease in activity. The authors believed that the high catalytic activity of this catalyst could be attributed to the nature of the applied ligand with stability against air and moisture, and the stabilizer acted to prevent agglomeration of palladium NPs.

**Scheme 63:**
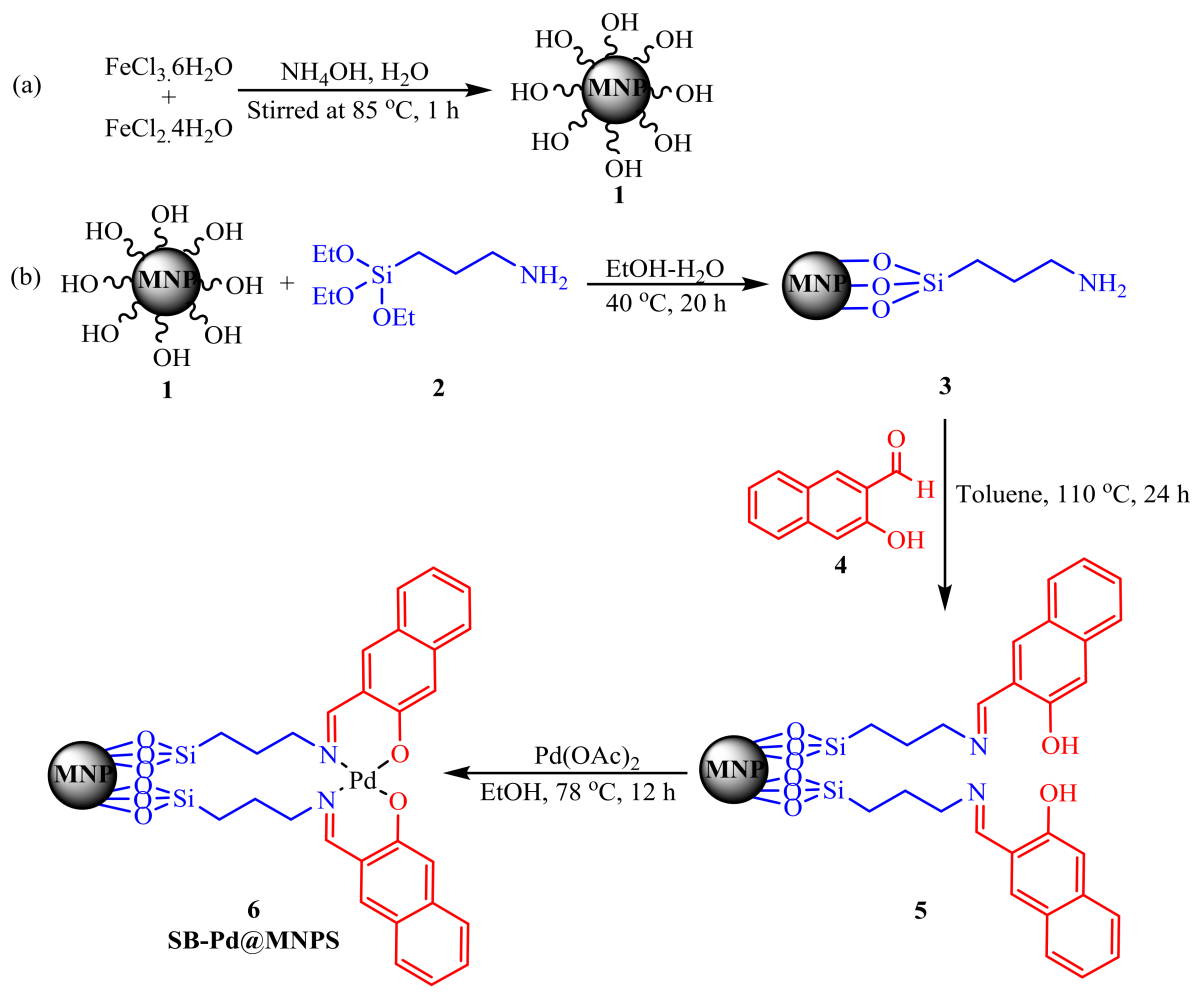
Synthetic route to (**a**) MNPs (**1**) and (**b**) SB-Pd@MNPs nanomagnetic catalyst (**6**). Reproduced with permission from [54].

Manjunatha and co-workers, using an anchoring Schiff base‒palladium(II) complex on magnetic NPs, synthesized a magnetically separable SB-Pd@MNPs catalyst (Scheme 63) [54]. The SB-Pd@MNPs nanomagnetic catalyst was prepared in four steps. **1** was prepared from a 2:1 molar ratio of Fe^3+^ and Fe^2+^ with ammonium hydroxide as base. Chemical co-precipitation. **3** was prepared via functionalization of **1** with **2** in EtOH–H_2_O mixture at 40 °C. Then, **5** was synthesized from the reaction between **4** and **3** in toluene at 110 °C. In the final step, **5** was treated with palladium(II) acetate in ethanol at 78 °C to afford the desired SB-Pd@MNPs nanomagnetic catalyst (**6**).

**Scheme 64:**
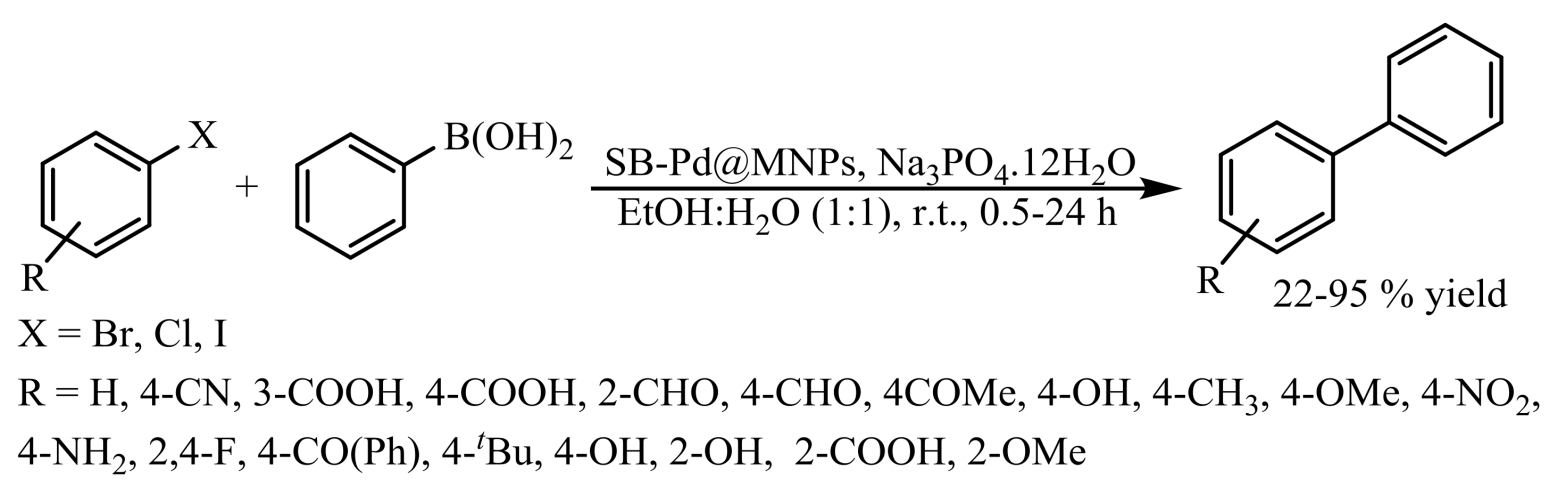
Suzuki‒Miyaura cross-coupling reactions between aryl halides and phenylboronic acid catalyzed by SB-Pd@MNPs nanomagnetic catalyst [54].

The SB-Pd@MNPs nanomagnetic catalyst was found to be an efficient catalyst for Suzuki‒Miyaura cross-coupling of various aryl halides with phenylboronic acid and for the reduction of nitroarenes in aqueous medium at room temperature. The best yields were obtained by carrying out the reaction at room temperature in EtOH:H_2_O (1:1) as the solvent with Na_3_PO_4_∙12H_2_O as the base (Scheme 64). The procedure was obtained for cross-coupling and reduction reactions by carrying out the reaction at ambient temperature and in water as an environmentally benign solvent. The catalyst can be recovered by external magnetic separation and reused up to five cycles without loss of catalytic activity.

### 2.5. Magnetic NanoparticleSupported N-Heterocyclic Carbene Complexes

**Scheme 65:**
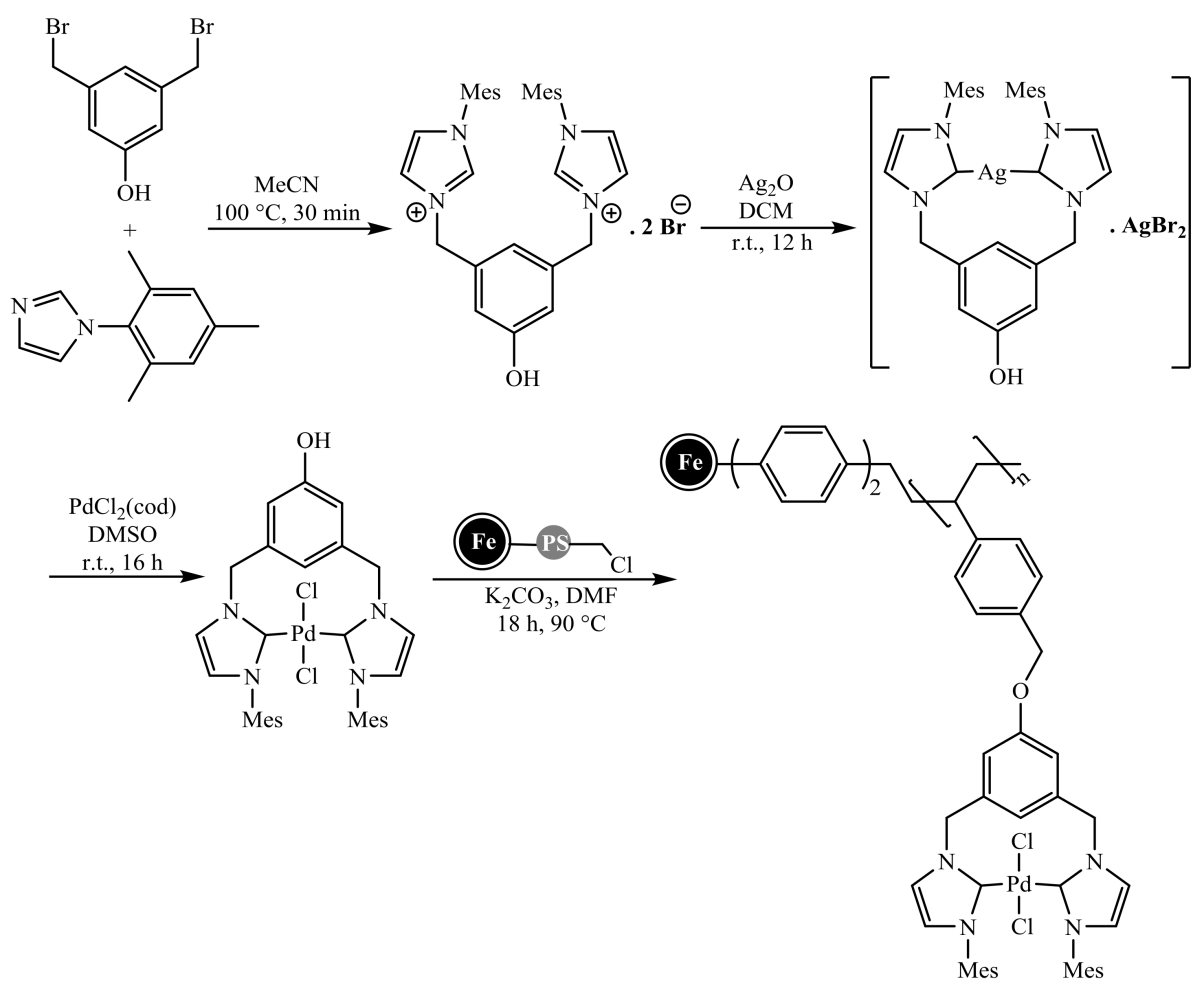
Synthesis of polystyrene-modified, carbon-coated iron NPs supported palladium complex. Reproduced with permission from [55].

In recent years, *N*-heterocyclic carbene (NHC) ligands, due to their high stability against heat, moisture, and air, have attracted increasing interest in the field of Pd-catalyzed C‒C coupling reactions. Immobilization of NHC-Pd catalysts on heterogeneous supports, by allowing facile recycling, enhanced the turnover numbers of the catalyst. A palladium bis-*N*-heterocyclic carbene complex was immobilized on polystyrene-modified carbon-coated iron NPs by Reiser and co-workers (Scheme 65) [55]. The authors suggested the encapsulation of palladium NPs in the polystyrene matrix of the support. 

**Scheme 66:**
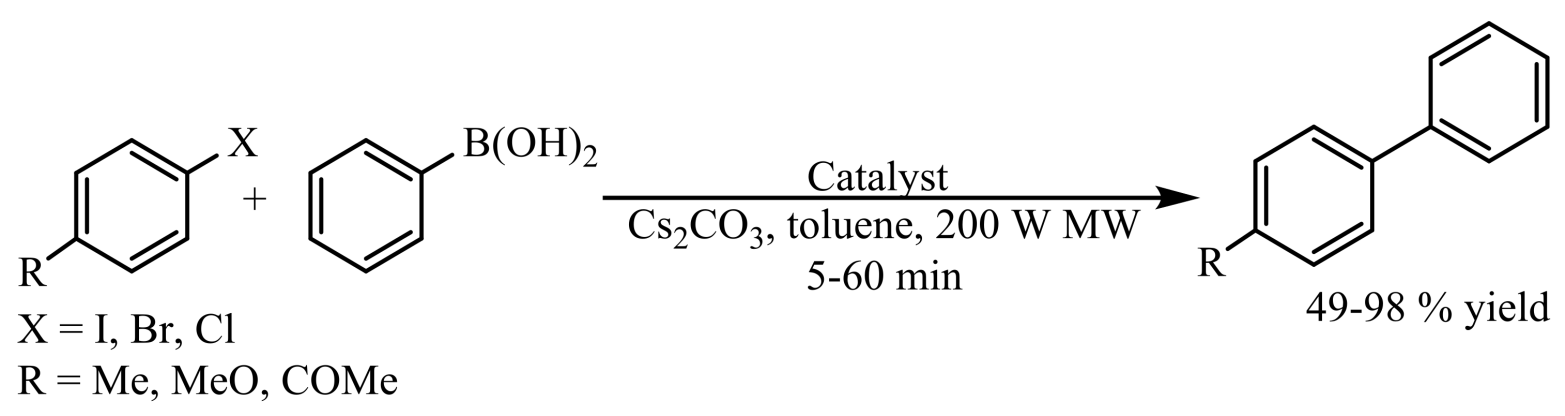
Suzuki‒Miyaura reaction catalyzed by carbon coated iron NPs supported palladium complex under microwave conditions.

The catalytic activity and recyclability of obtained catalyst has been tested in Suzuki‒Miyaura cross-coupling reactions of aryl halides and phenylboronic acid in toluene by using Cs_2_CO_3_ as the base under microwave irradiation (Scheme 66). An external magnetic was utilized to recycle the catalyst, and the effectiveness of the catalyst is retained for at least four cycles. 

**Scheme 67:**
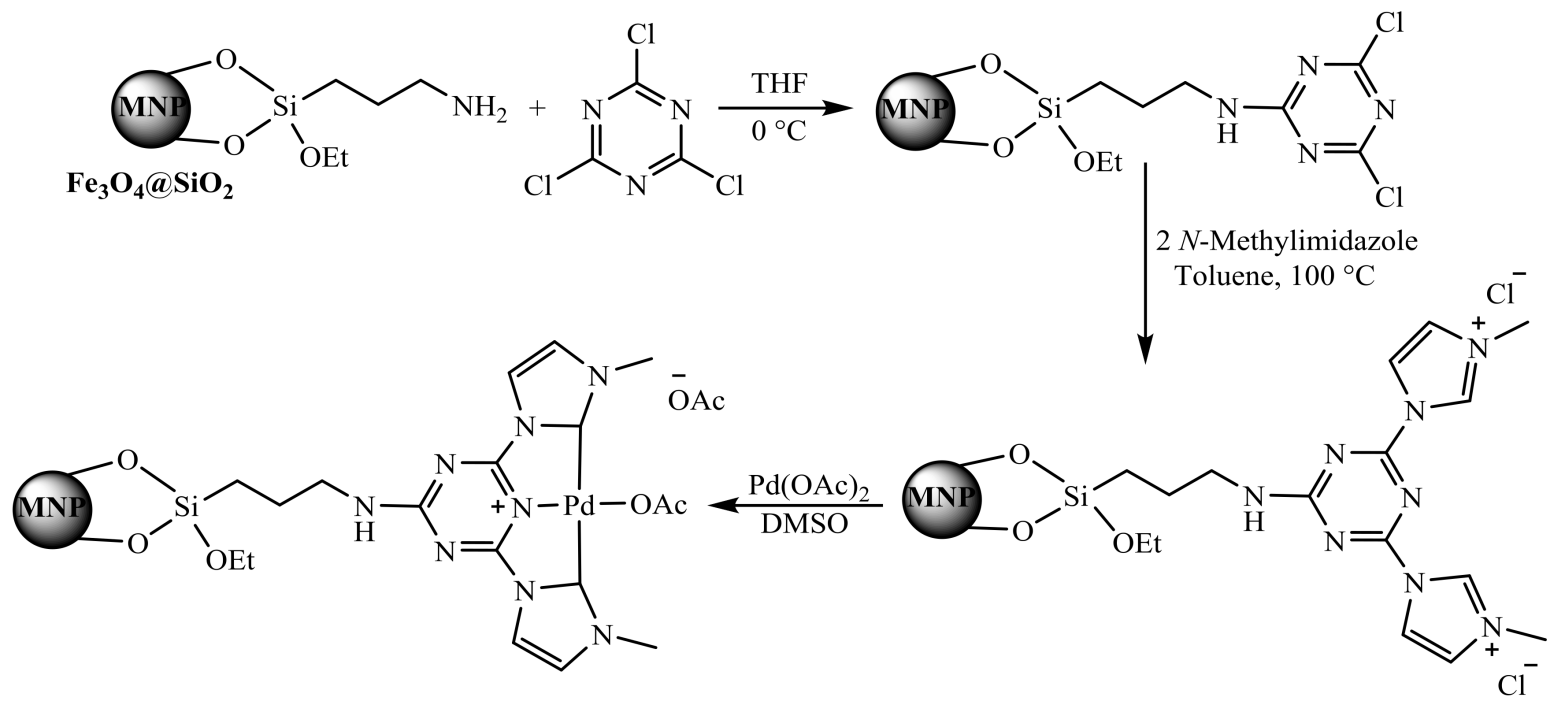
Synthesis of the catalyst SPIONs-bis(NHC)-palladium(II) diacetate. Reproduced with permission from [56].

Khosropour and co-workers reported designation and preparation of SPIONs (superparamagnetic iron oxide NPs)-bis(NHC)-palladium(II) as Pd-NHC-tagged MNPs [56]. Reaction of amino functionalized Fe_3_O_4_ with 1,3,5-trichlorotriazine (TCT) was studied by controlling the temperature, followed by replacing *N*-methylimidazole with two other chlorides, resulting in the preparation of a bidentate NHC ligand via the formation of a C‒N bond between imidazole and triazine parts (Scheme 67). Finally, SPIONs-bis(NHC)-palladium(II) diacetate was prepared from the reaction between supported *N*-heterocyclic carbene ligand with Pd(OAc)_2_ in DMSO.

**Scheme 68:**
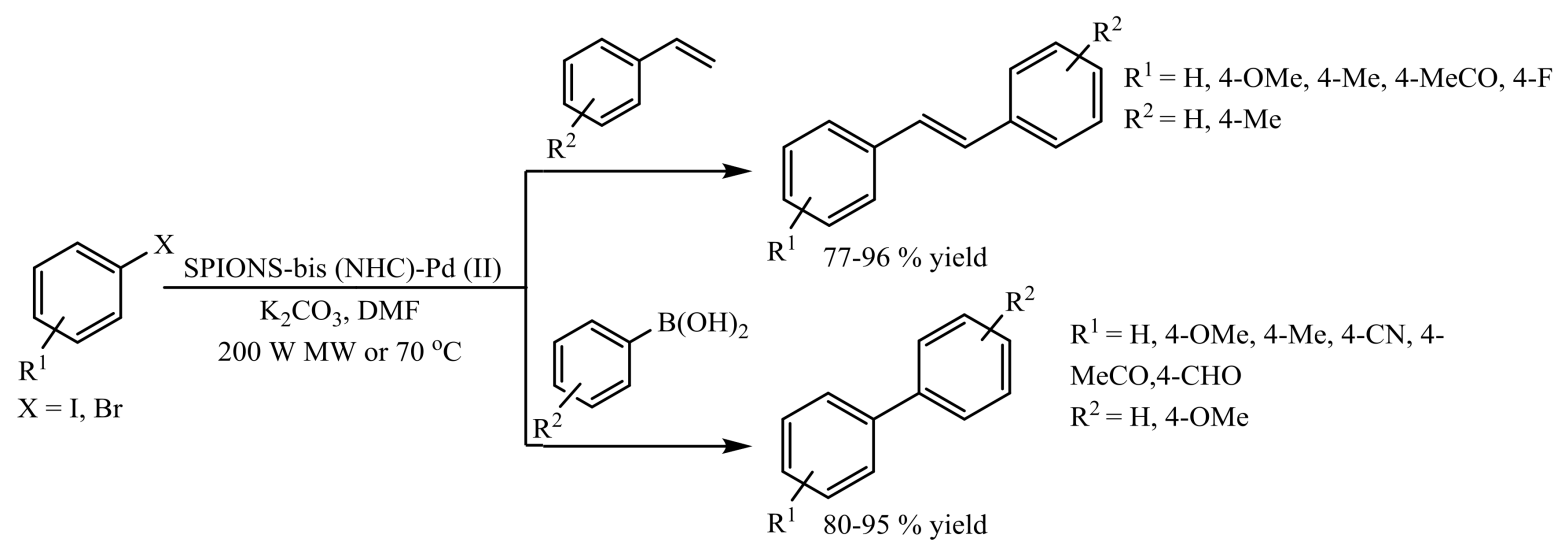
Suzuki‒Miyaura and Heck cross coupling reactions in the presence of SPIONS-bis (NHC)-Pd(II) [56].

The obtained Pd‒NHC complex acted as a powerful nanocatalyst in Heck and Suzuki‒Miyaura C‒C coupling reactions under heating or microwave irradiation, which provided high yields and TOF (Scheme 68). Moreover, the catalyst could be recovered by using an external magnet and reused for seven runs without any change in catalytic activity.

**Scheme 69:**
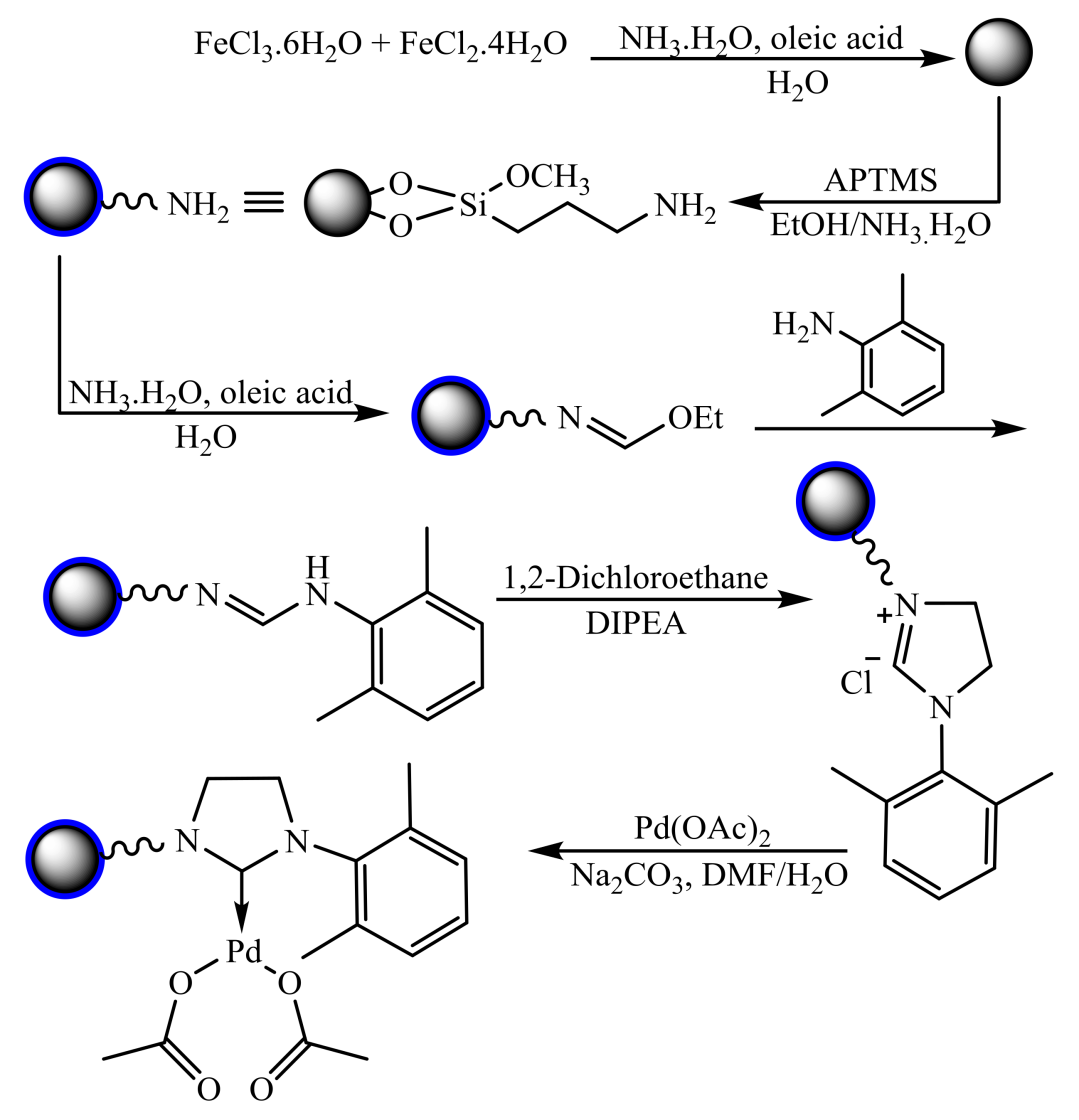
Synthesis of MNP@NHC-Pd. Reproduced with permission from [57].

Direct synthesis of imidazolinium salt supported on magnetic NPs and immobilization of Pd(OAc)_2_ onto the surface of modified Fe_3_O_4_ NPs has been reported by Wilczewska and Misztalewska [57]. Nanocatalyst (MNP@NHC-Pd) was prepared by direct synthesis of imidazolinium salt on amino functionalized Fe_3_O_4_ NPs through the “grafting from” immobilization in a three-step approach followed by a reaction with a solution of Pd(OAc)_2_ and aqueous solution of Na_2_CO_3_ in DMF (Scheme 69). In this work, the silane shell was prepared by condensation reaction of APTMS ((3-aminopropyl)-trimethoxysilane) on the surface of the MNP.

**Scheme 70:**
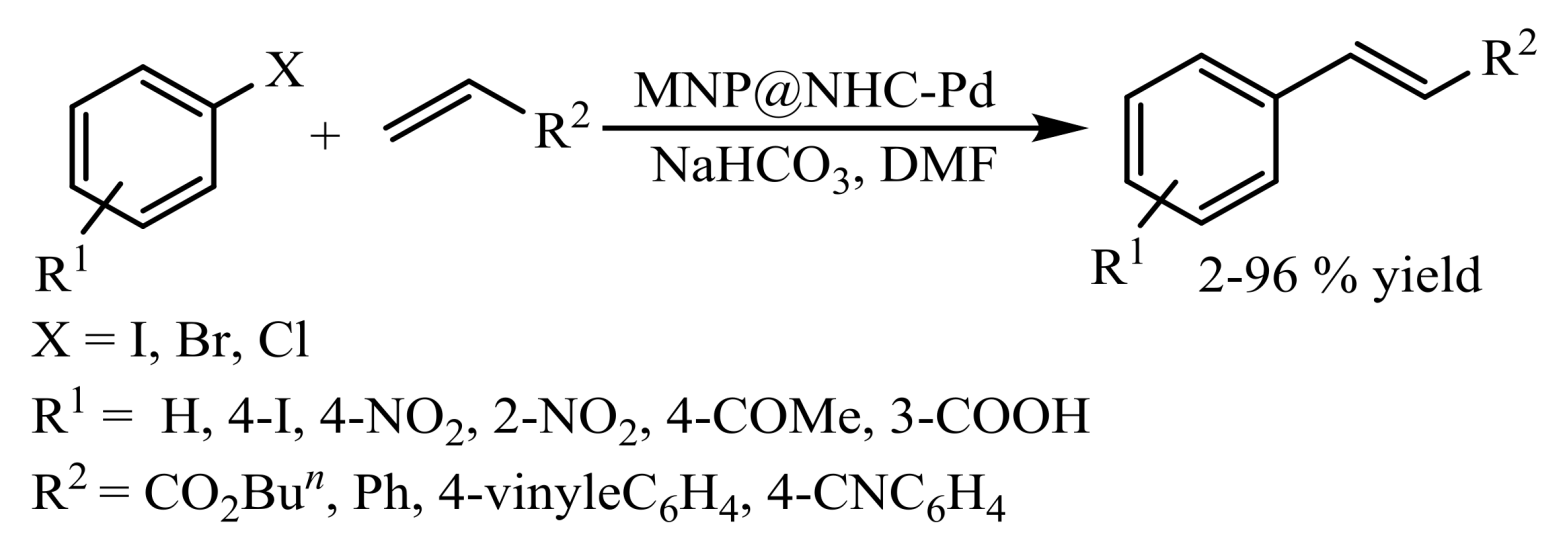
Heck coupling reaction of various aryl halides and vinyl compounds under MNP@NHC-Pd.

The activity of obtained MNP@NHC-Pd has been evaluated in the Heck coupling reaction of various aryl halides and vinyl compounds in the presence of NaHCO_3_ as the base in DMF (Scheme 70). The supported catalyst can be removed from the reaction mixture and reused at least five times without loss of its activity.

**Scheme 71:**
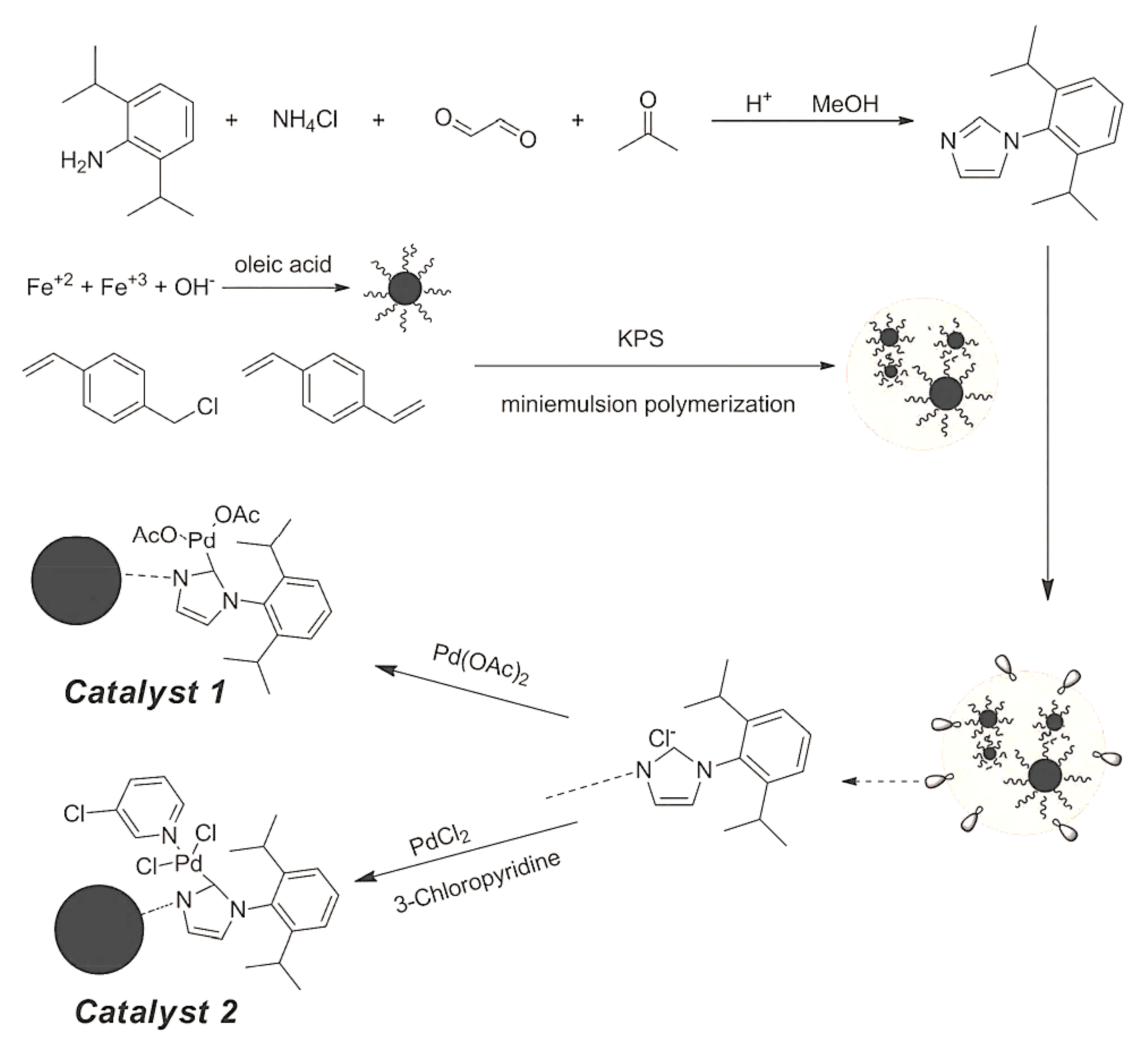
The preparation procedures of magnetic palladium catalysts. Reproduced with permission from [58].

**Scheme 72:**
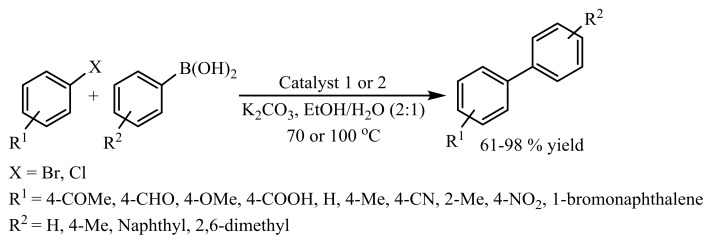
Catalytic efficiencies of magnetic catalysts 1 and 2 in Suzuki‒Miyaura coupling reaction.

Wang et al. prepared a palladium catalyst with *N*-heterocyclic carbene (NHC) ligand on polymer magnetic carrier with different sources of Pd salts, Pd(OAc)_2_ and (PdCl_2_ and 3-Cl-pyridinyl) and investigated the catalytic activity and stability of the obtained catalyst in a Suzuki‒Miyaura cross-coupling reaction [58]. Magnetic carriers were synthesized through mini-emulsion polymerization; after modification of the surface of these magnetic carriers with 1-arylimidazole, different palladium salts were employed for the preparation of catalyst-1 and catalyst-2 (Scheme 71). Catalyst-1 showed high catalytic activity in the reaction of phenylboronic acids with aryl bromides in the ethanol‒water solution (Scheme 72). The catalytic activity of catalyst-1 slightly decreased after 21 runs, whereas catalyst-2 showed more stability and activity, as performed for Suzuki‒Miyaura reaction with aryl chlorides at 100 °C. 

**Scheme 73:**
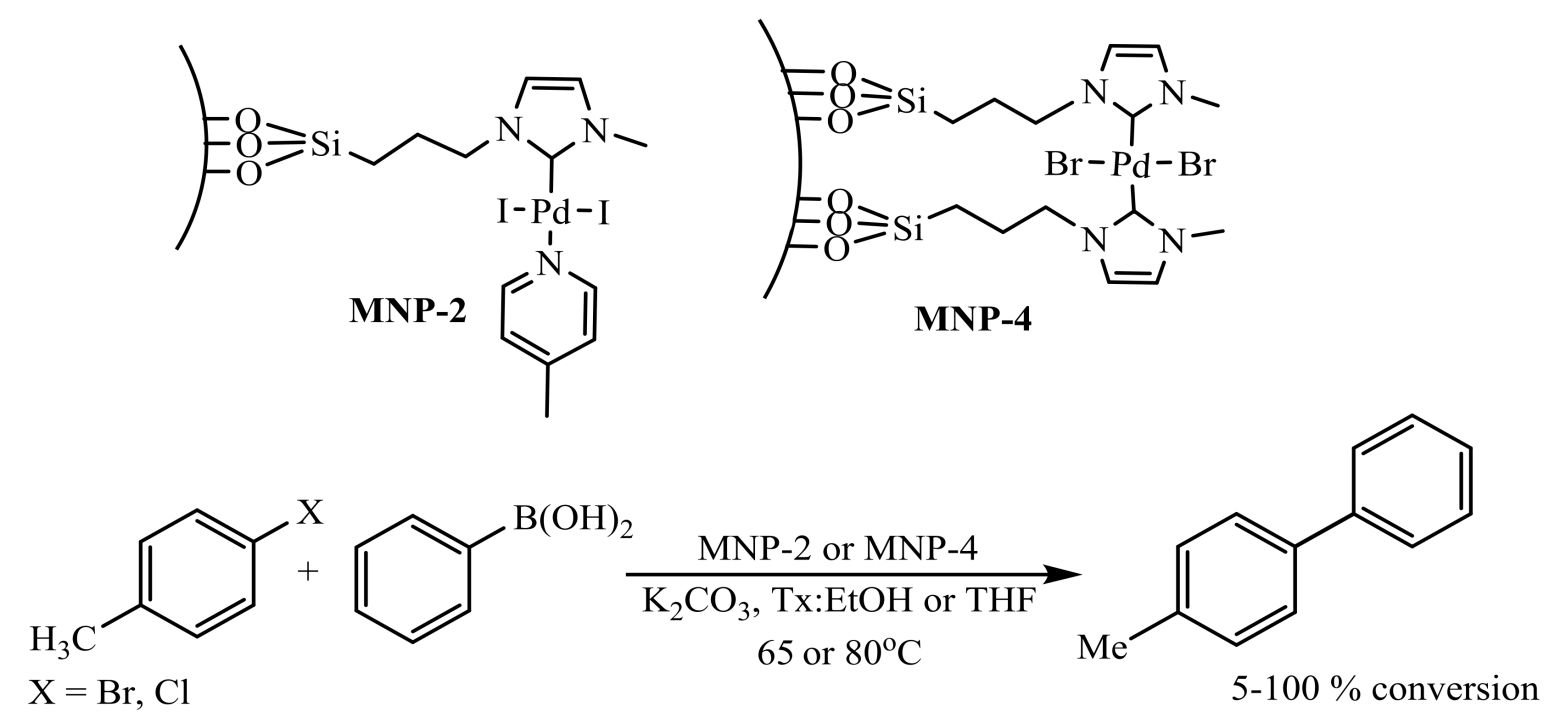
Comparison of catalysts (MNP-2 and MNP-4) for the Suzuki‒Miyaura reaction of 4-halotoluenes with phenylboronic acid.

Martínez-Olid and co-workers reported mono- or bis-(NHC) complexes of palladium and a magnetically recoverable catalyst based on these complexes for the Suzuki‒Miyaura reaction [59]. The precatalysts were based on a PEPPSI-type complex and a bis(NHC) related compound. Synthesized palladium complexes containing alkoxyorganosilyl groups undergo condensation reactions with magnetic core/shell γ-Fe_2_O_3_/silica particles, and, through covalent immobilization, yielded magnetically recoverable catalysts based on mono- or bis-(NHC) complexes of palladium (MNP-2 and -4). Comparative studies of the catalytic performance of complexes and their immobilization were conducted. For this purpose, unsupported (2 and 4) and supported complexes (MNP-2 and -4) were tested in the Suzuki‒Miyaura reaction of 4-halotoluenes and phenylboronic acid in a 1:1 mixture of Tx (a 0.21 wt % aqueous solution of Triton™ X-405) and ethanol. Overall, the magnetic catalysts with high performance and magnetic recovery ability were found to be more reactive than the homogeneous complexes. A comparative study of two heterogeneous catalysts revealed that the bis(NHC) immobilized complex with lower Pd loading is a robust catalyst that can operate under very mild conditions, even for the activation of chloroarenes, whereas its analog based on the mono(NHC) compound rapidly deactivates (Scheme 73).

**Figure 22:**
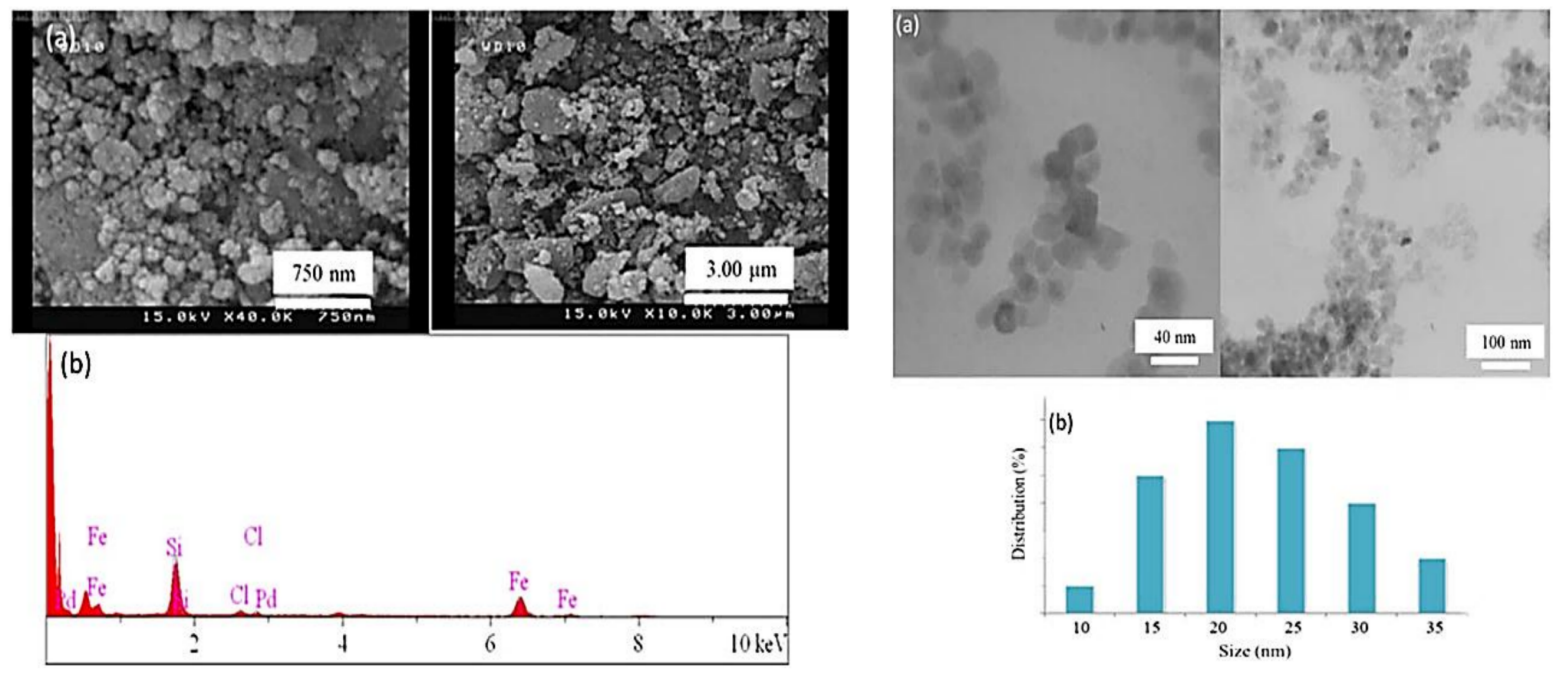
Left. (**a**) FE-SEM images and (**b**) SEM-EDX, right. (**a**) TEM images and (**b**) particle size distribution for magnetic iron oxide NPs-*N*-heterocyclic carbine-palladium(II). Reproduced with permission from [60].

The immobilization of a palladium NHC complex on magnetic iron oxide NPs was achieved by Hajipour and co-workers [60]. They first anchored phenyl-1*H*-imidazole on the surface of silica modified Fe_3_O_4_ NPs and finished the complex formation by treatment with NaPdCl_3_. FE-SEM and EDX results showed the presence of Pd NPs in the catalyst and TEM images revealed well-defined dispersed spherical palladium particles with a size of about 15–35 nm in a silica bed (Figure 22).

**Scheme 74:**
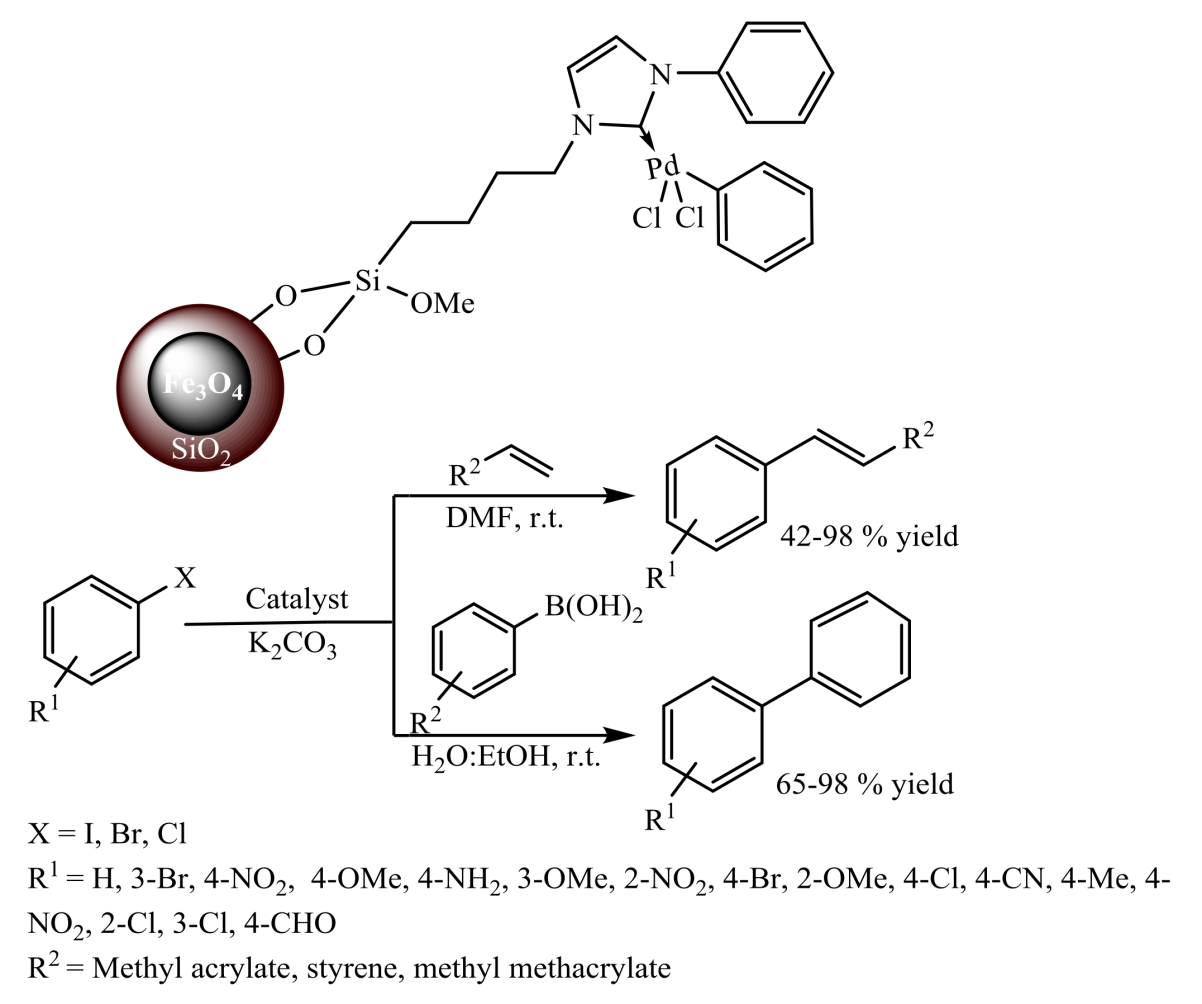
Suzuki‒Miyaura and Heck cross-coupling in the presence of magnetic iron oxide NPs-*N*-heterocyclic carbine-palladium(II) [60].

An air- and moisture-stable catalyst was found to be an efficient and robust recyclable catalyst in the Heck and Suzuki‒Miyaura cross-coupling reactions under mild conditions and products were obtained in good to excellent yields (Scheme 74). At the end of each reaction, catalyst recycling was performed; the catalyst was separated using a magnetic field, washed with ethanol and acetone, and reused at least 10 times without significant loss of its catalytic activity (90% yield after the tenth run) or Pd leaching (less than 1%). 

**Scheme 75:**
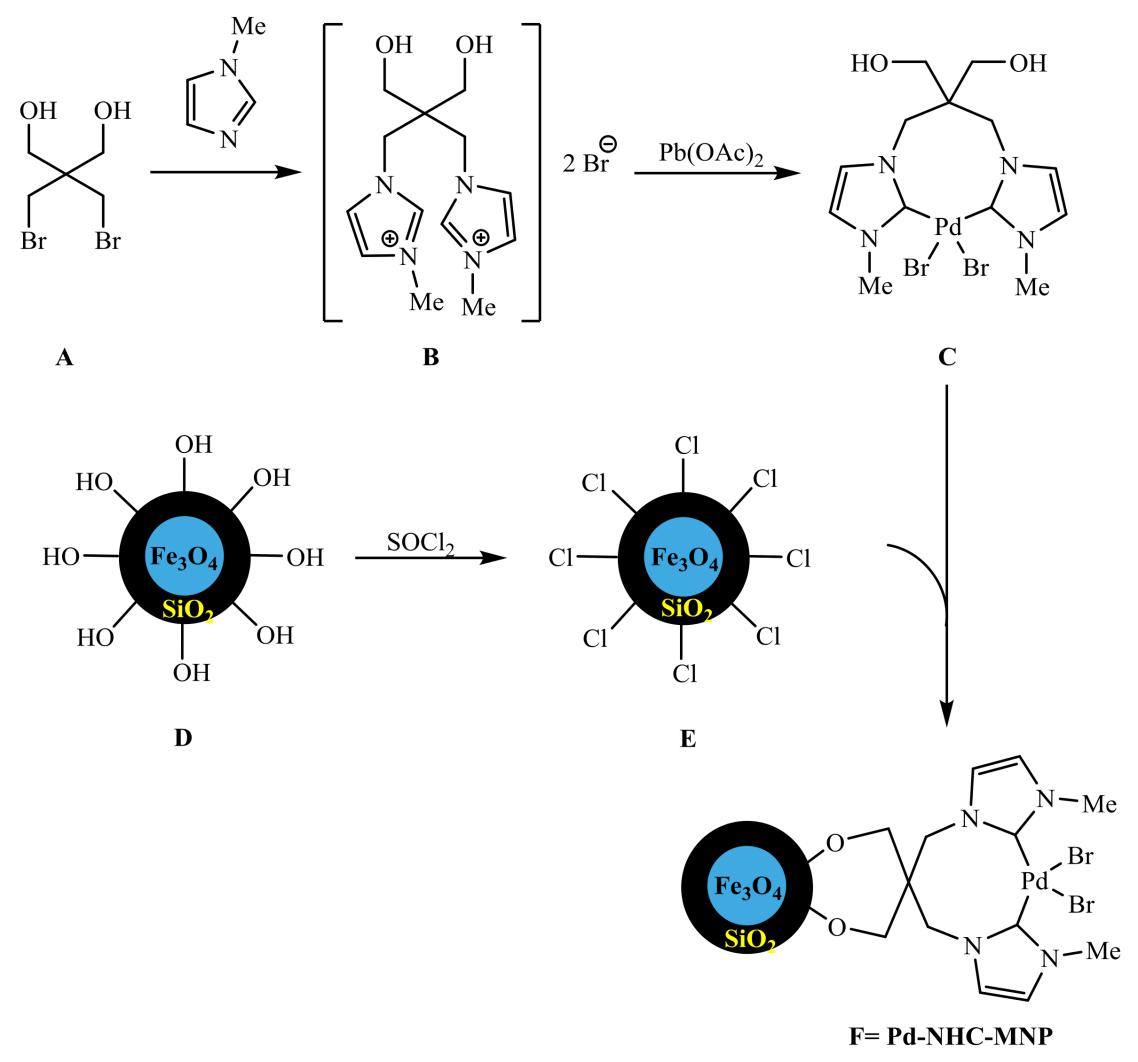
Synthetic route toward synthesis of Pd-NHC-MNP catalyst. Reproduced with permission from [61].

Faregh-Alamdari and co-workers synthesized a catalyst for a Suzuki‒Miyaura cross-coupling reaction based on bis(*N*-heterocyclic carbene) palladium complex supported on silica-coated magnetic NPs (MNPs) by treatment of the magnetic NPs (D) with thionylchloride. In this approach, the Pd-NHC complex (C) was prepared from the reaction of a hydroxyl-functionalized bis-imidazolium ionic liquid (B) with Pd(OAc)_2_ (Scheme 75) [61].

**Scheme 76:**
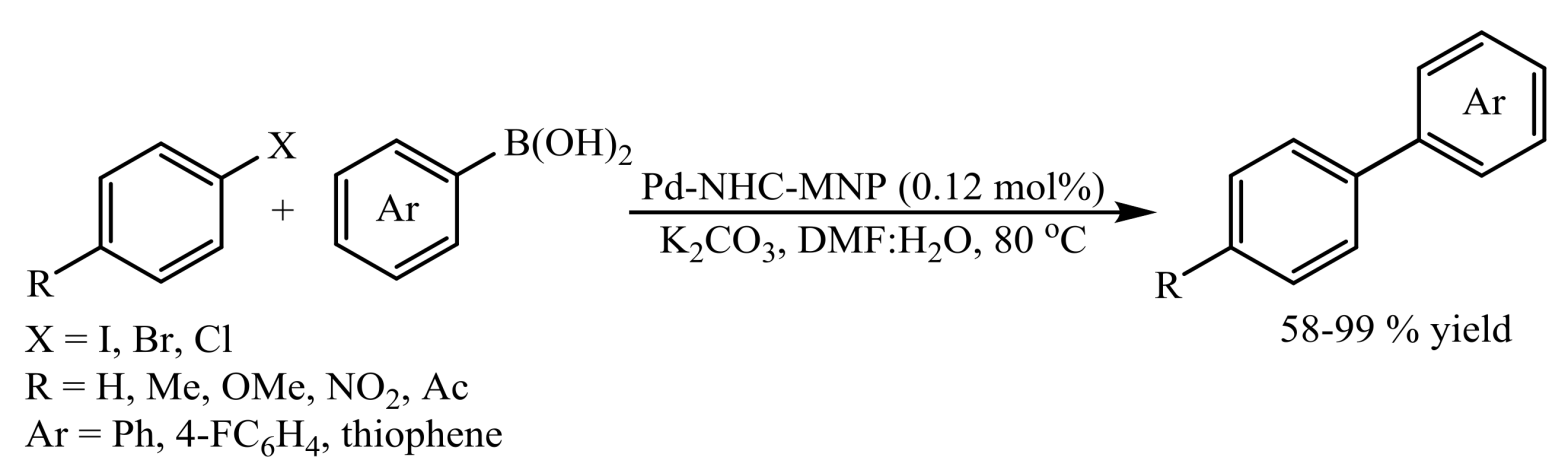
Suzuki‒Miyaura cross-coupling reactions of aryl halides and arylboronic acids catalyzed by Pd-NHC-MNP.

The Pd-NHC-MNP catalyst was applied for the Suzuki‒Miyaura reaction of various aryl halides and arylboronic acids in the presence of K_2_CO_3_ in DMF/H_2_O (2:1) at 80 °C to furnish diverse biaryl products after 0.8–2.15 h in yields of 58–99% (Scheme 76). Also, this catalyst can be magnetically separated from the reaction mixture of 4-iodoanisole and phenylboronic acid and washed thoroughly with acetone, diethyl ether, and water, and subsequently reused in six reaction runs with only a slight decrease in yield (97% to 88%).

**Scheme 77:**
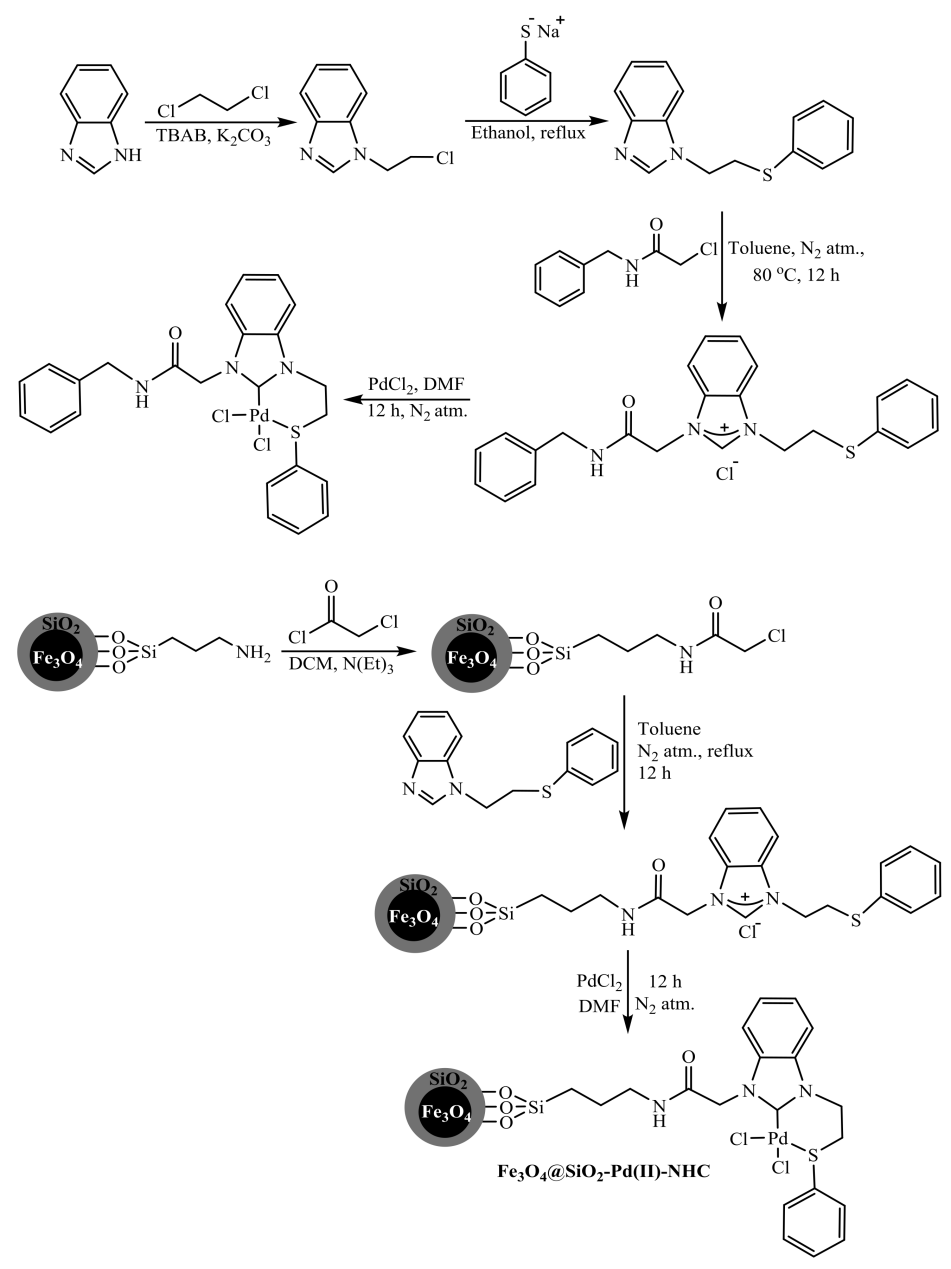
Strategy used for the preparation of Pd(II)-NHC complex and solid-supported magnetically retrievable Fe_3_O_4_@SiO_2_-Pd(II)-NHC catalyst. Reproduced with permission from [62].

Sharma and co-workers synthesized a novel homogeneous Pd(II) catalyst incorporating a chelating thioether-NHC ligand. Moreover, to overcome the issue of the reusability of the catalyst and study the catalytic activity of the homo- and heterogeneous versions of the catalyst, the Pd complex was supported on aminopropyl-functionalized silica-coated magnetite NPs (Scheme 77) [62].

**Scheme 78:**
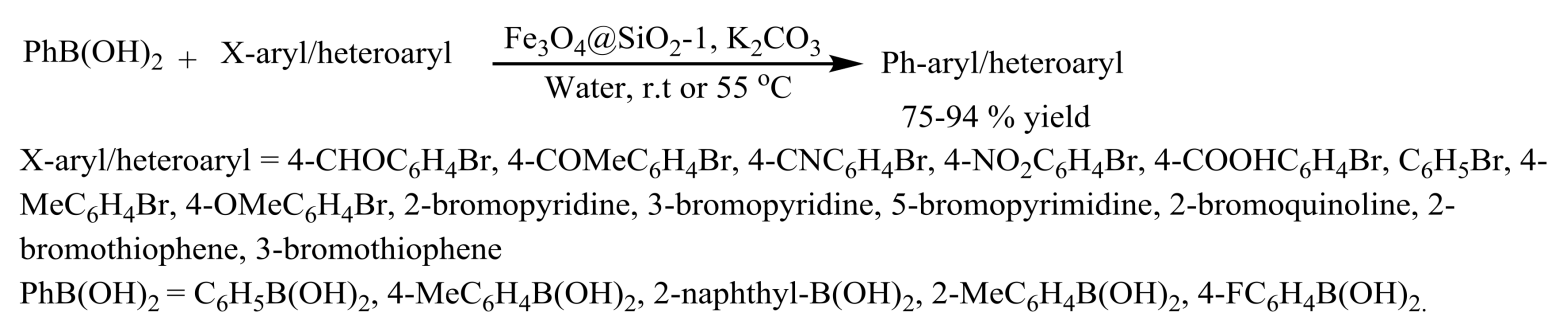
Catalytic investigation of Fe_3_O_4_@SiO_2_-Pd(II)-NHC catalyst in the Suzuki‒Miyaura coupling of aryl/heteroaryl bromides with arylboronic acids [62].

The catalyst was applied for the coupling reactions of various aryl/heteroaryl bromides in water in the presence of K_2_CO_3_ at room temperature (Scheme 78). Pd leaching was investigated by ICP for both Pd(II)-NHC complex 1 and Pd(II)-NHC catalyst Fe_3_O_4_@SiO_2_-Pd(II)-NHC. The results revealed the homogeneity of the Pd(II)-NHC complex; also, the negligible concentration of palladium in Fe_3_O_4_@SiO_2_-Pd(II)-NHC showed the largely heterogeneous nature of this catalyst. A comparative study of the catalytic activity of the homo- and heterogeneous versions of the catalyst demonstrated higher catalytic activity in terms of catalyst loading for Pd(II)-NHC complex, but deactivated after one cycle. However, recycling experiments for Fe_3_O_4_@SiO_2_-Pd(II)-NHC showed that the catalyst was easily separated from the reaction medium by using an external magnet and showed its activity for up to seven cycles.

**Figure 23:**
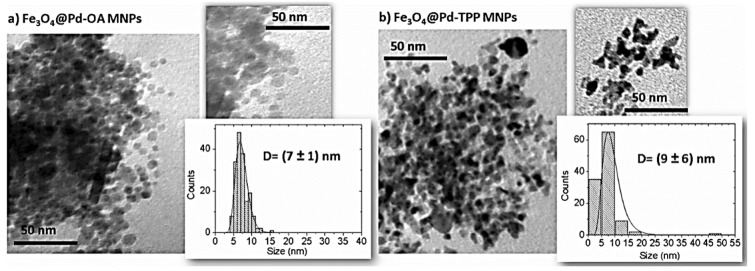
TEM micrographs for core-shell Fe_3_O_4_@Pd-OA (**a**) and Fe_3_O_4_@Pd-TPP (**b**). Reproduced with permission from [63].

Martín and co-workers reported the core-shell Fe_3_O_4_@Pd superparamagnetic NPs (MNPs) by metal complex thermal decomposition and investigated the role of different stabilizers such as oleylamine (OA), triphenylphosphine (TPP) and triphenylamine (TPA) in the synthesis and catalytic activity of MNPs [63]. Among the three stabilizers, the OA with dual function (both reducing and stabilizing) was capable of producing spherical Pd NPs with small size and uniform shell around the magnetite core, which is confirmed with HRTEM, TGA, magnetic behavior, and dark-field images (Figure 23). In addition to the roles described above, OA prevents the oxidation of the Pd(0) shell.

**Scheme 79:**
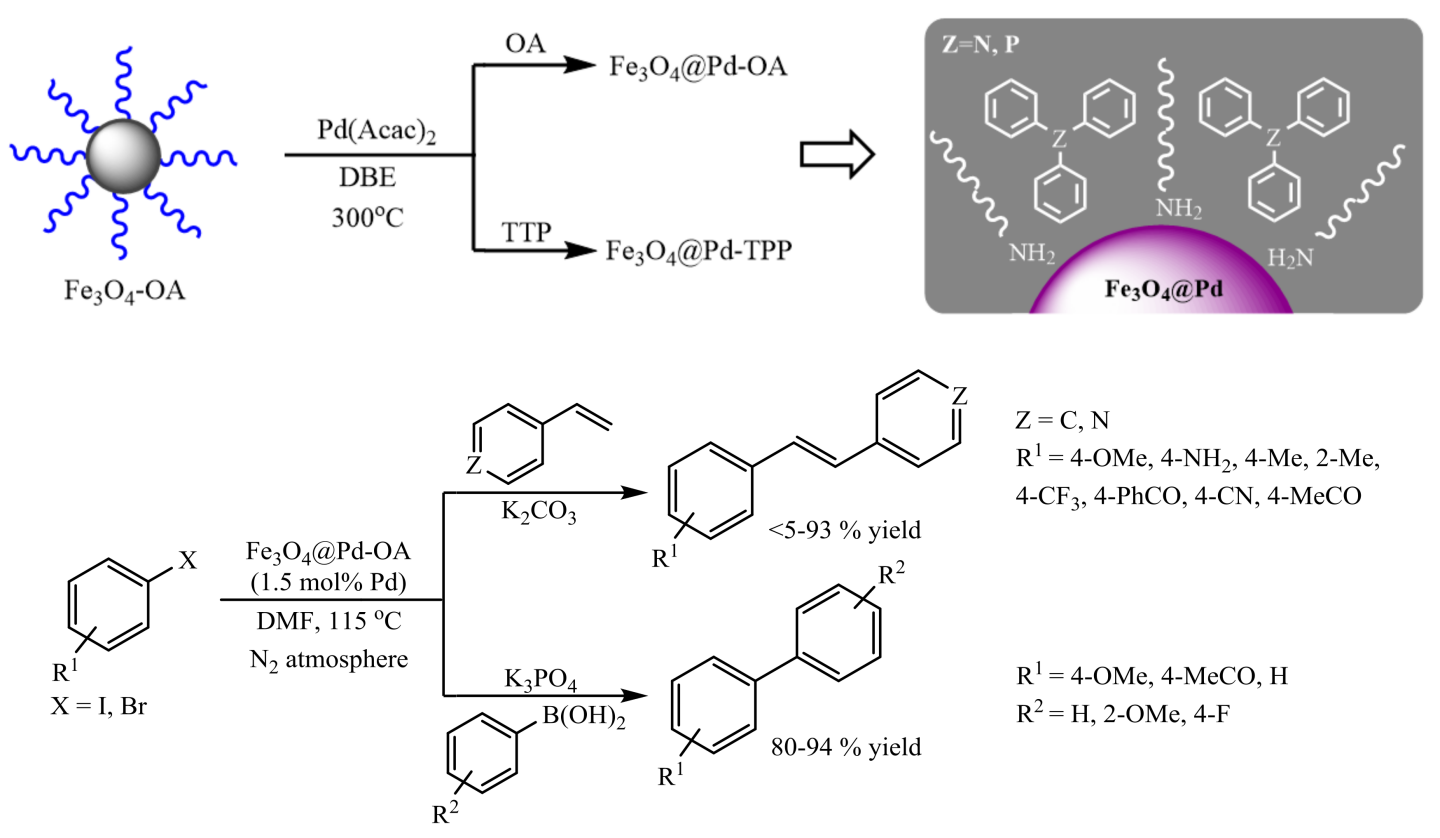
Catalytic activity of Fe_3_O_4_@Pd-OA in Heck coupling and Suzuki‒Miyaura cross-coupling reactions. Reproduced with permission from [63].

The resulting core-shell MNPs catalyst was demonstrated to be highly active for Heck and Suzuki‒Miyaura coupling reactions for the synthesis of stilbenes and biaryl compounds (Scheme 79). In addition, the recyclability of Fe_3_O_4_@Pd-OA MNPs was evaluated in a C‒C coupling reaction and showed good activity for at least four cycles. 

**Figure 24:**
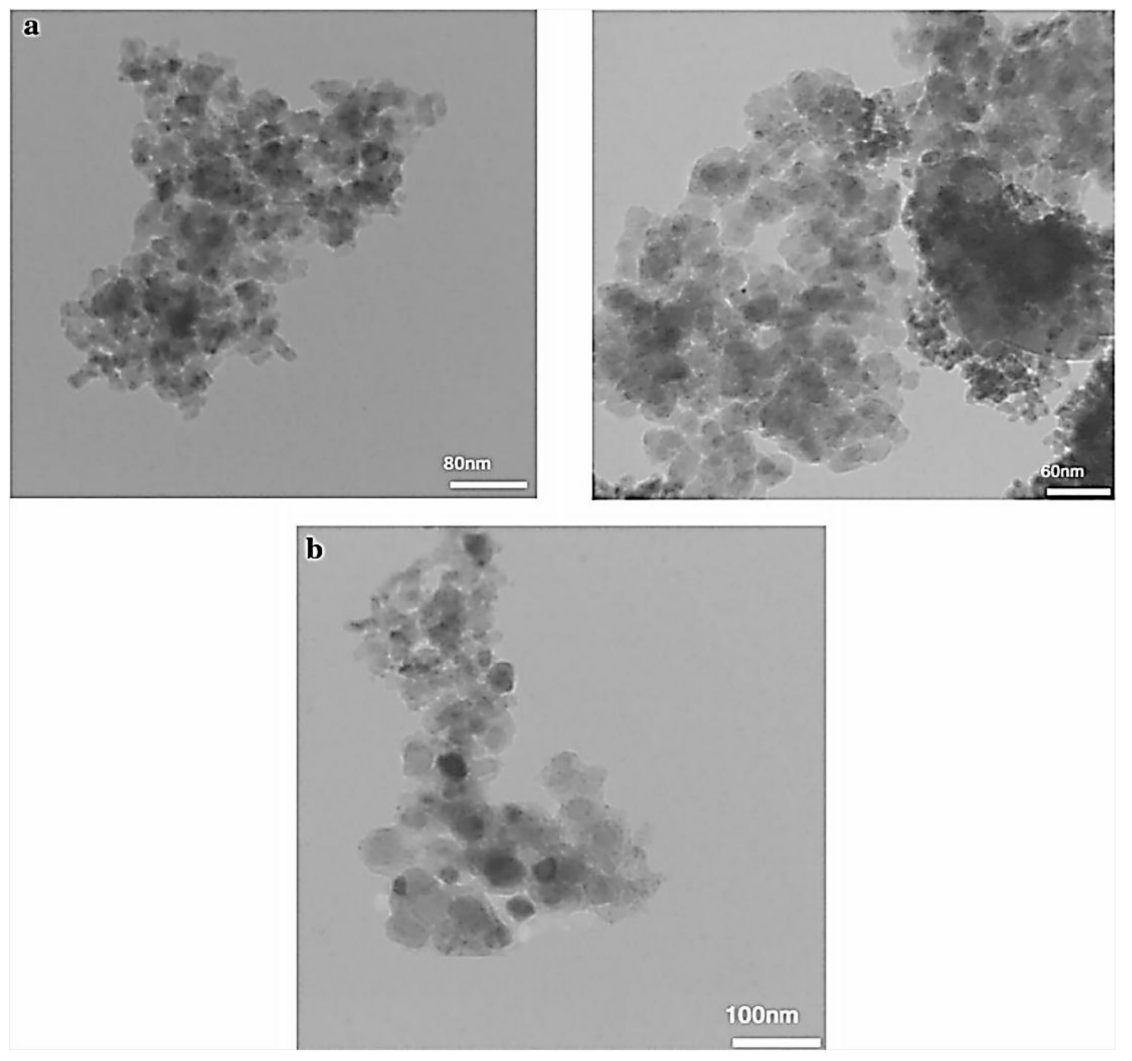
TEM images of fresh (**a**) and four-times reused Fe_3_O_4_@SiO_2_@VB1-Pd nanocatalyst (**b**). Reproduced with permission from [64].

Rafiee and Mehdizadeh modified the surface of silica-coated Fe_3_O_4_ NPs with a palladium *N*-heterocyclic carbene complex of vitamin B1. The Fe_3_O_4_ NPs were synthesized through a co-precipitation procedure, hydrolyzing TEOS in the presence of these NPs followed by covalent connection with thiamine hydrochloride (VB1) to obtain an *N*-heterocyclic carbene ligand for the formation of complex with palladium [64]. The resulting nanocomposite was composed of well-dispersed spherical Pd NPs with average size < 5 nm (Figure 24).

**Scheme 80:**
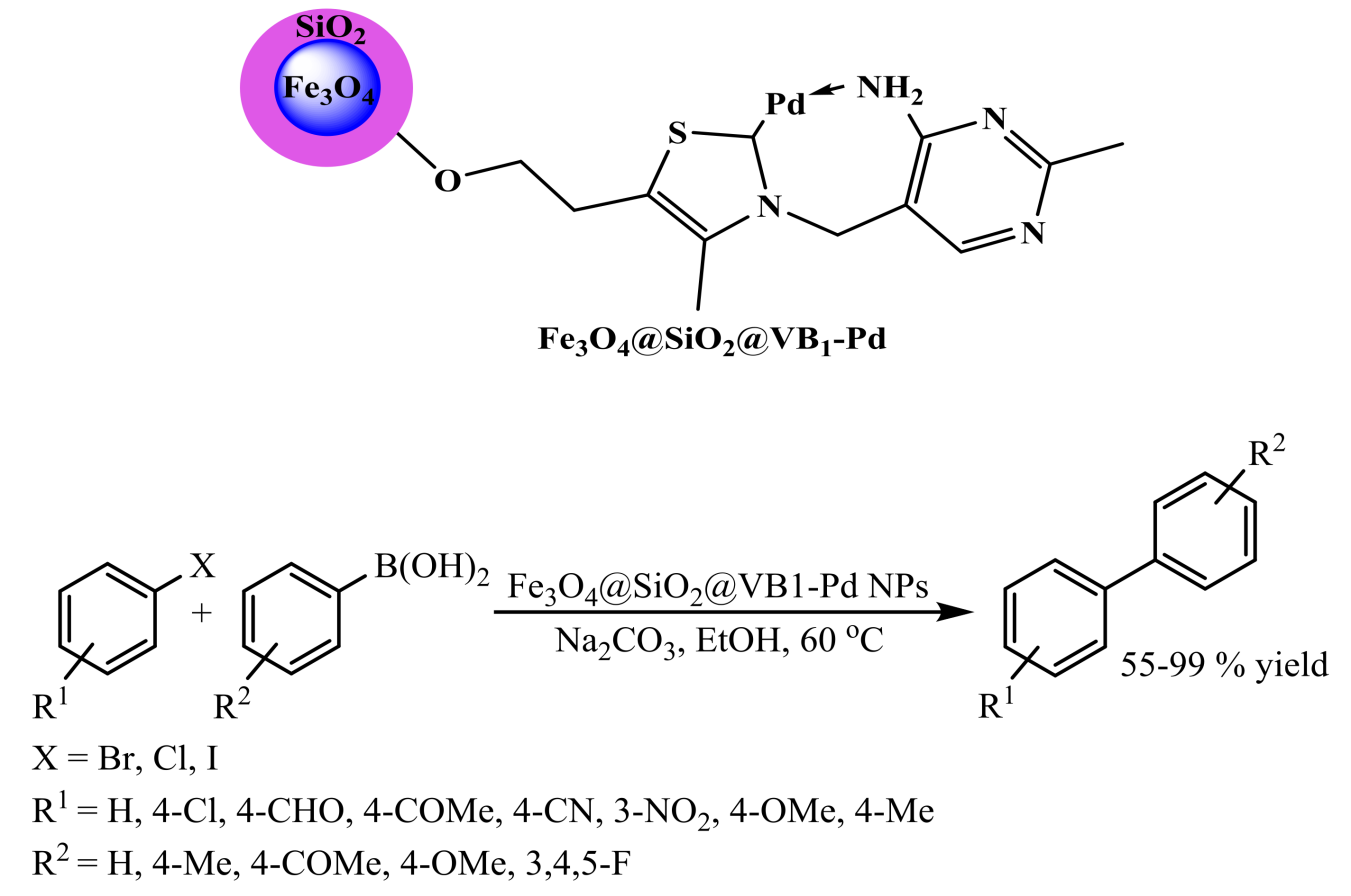
The synthesis of various biphenyl derivatives using Fe_3_O_4_@SiO_2_@VB1-Pd catalyst [64].

This catalyst was active in the Suzuki‒Miyaura coupling reaction of aryl halides with substituted arylboronic acids in the presence of Fe_3_O_4_@SiO_2_@VB1-Pd in EtOH at 60 °C resulted in the formation of biaryl products in 55–99% yield (Scheme 80). After completion of the reaction, the catalyst separated magnetically from the reaction mixture. After washing with ethanol and drying, it was applied for the next reaction and showed good yield and suitable time for five runs.

### 2.6. Magnetic Porous Nanocomposites

**Scheme 81:**
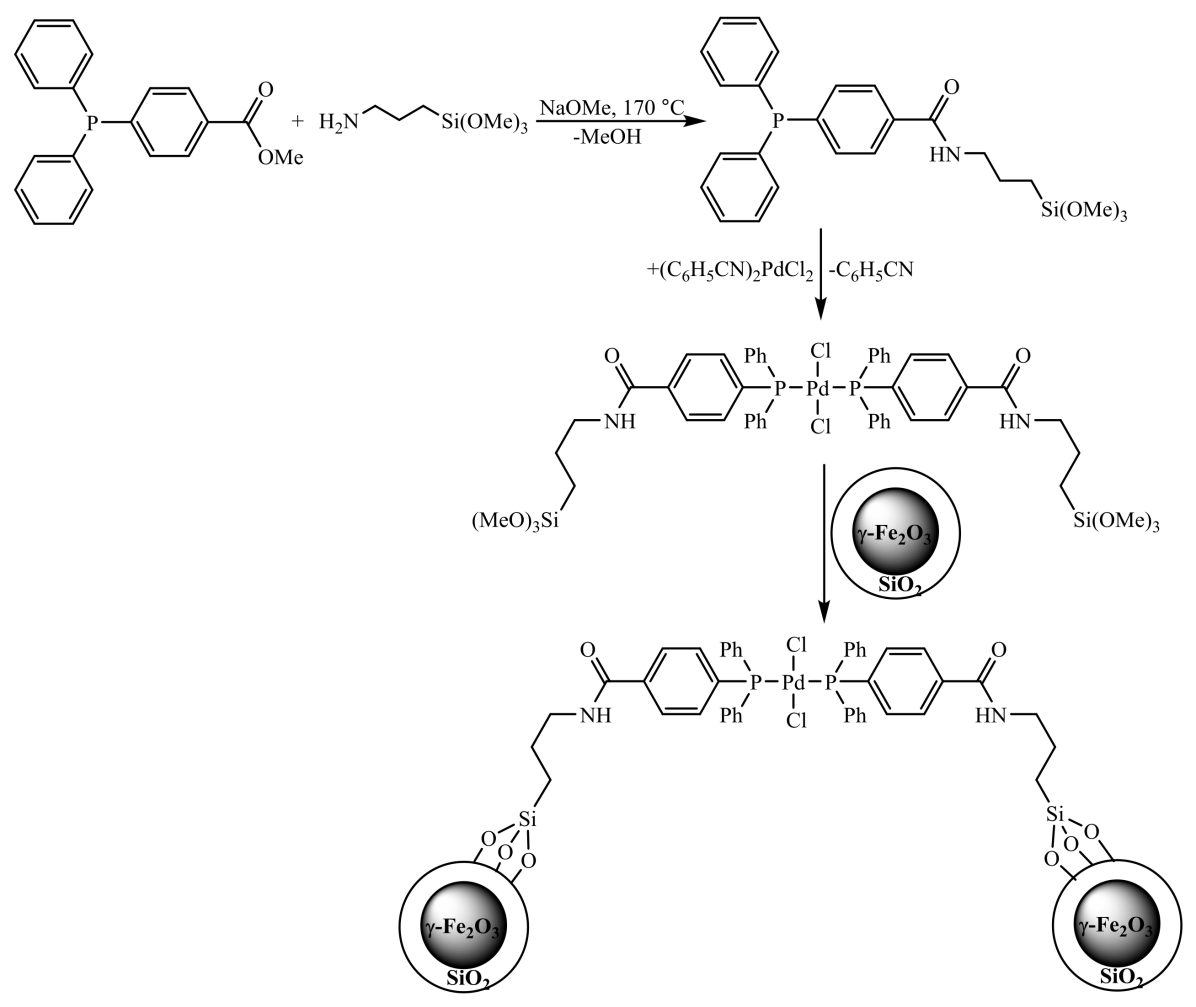
Magnetic mesoporous silica nanosphere immobilized Pd complex [65].

In 2010, Thiel and co-workers prepared a heterogeneous catalyst for the Suzuki‒Miyaura cross-coupling reaction by covalent grafting of a trimethoxysilyl-functionalized palladium(II) phosphane complex on silica-coated maghemite NPs (Scheme 81) [65].

**Scheme 82:**
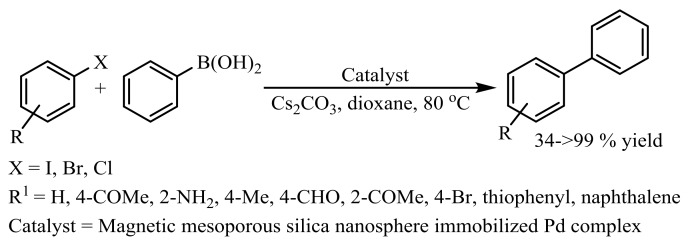
Suzuki‒Miyaura cross coupling of aryl halides and phenylboronic acid in the presence of magnetic mesoporous silica nanosphere immobilized Pd complex [65].

This heterogeneous nanocatalyst in dioxane and in the presence of Cs_2_CO_3_ at 80 °C catalyzed a coupling reaction of aryl iodides or bromides with phenylboronic acid (Scheme 82). The authors reported the efficiency of this catalyst, by comparison of the activity and stability between this reported catalyst and heterogenized catalysts such as Pd supported on commercially available silica gel and mesoporous MCM-41 in a Suzuki‒Miyaura reaction. The PPh_3_-Pd@SiO_2_/Fe_2_O_3_ could easily be recovered by a magnet, and after washing and drying, it can be reused seven times without a marked loss of its catalytic activity.

**Scheme 83:**
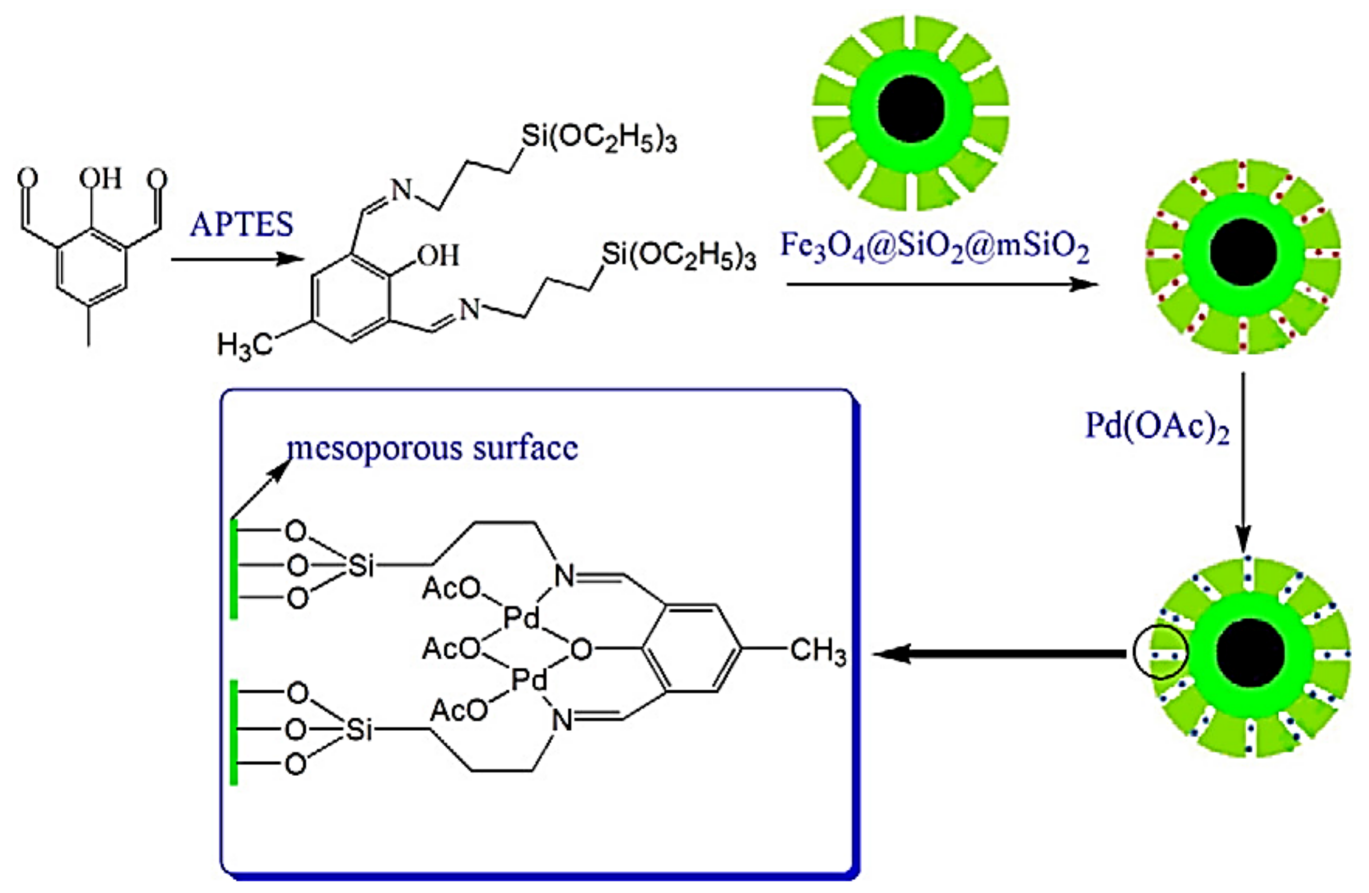
Preparation of the Fe_3_O_4_@SiO_2_@mSiO_2_-Pd(II) catalyst. Reproduced with permission from [66].

Dong and co-workers, through the functionalization of core-shell magnetic mesoporous material with a Pd(II) complex, prepared a recyclable catalyst for a Suzuki‒Miyaura cross-coupling reaction [66]. Refluxing 2,6-diformyl-4-methylphenol with APTES in toluene, followed by the addition of mesoporous material Fe_3_O_4_@SiO_2_@mSiO_2_, resulted in the preparation of ligand-functionalized magnetic mesoporous; finally, treating this material with Pd(OAc)_2_ led to the formation of a magnetic mesoporous Pd(II) complex (Scheme 83).

**Scheme 84:**
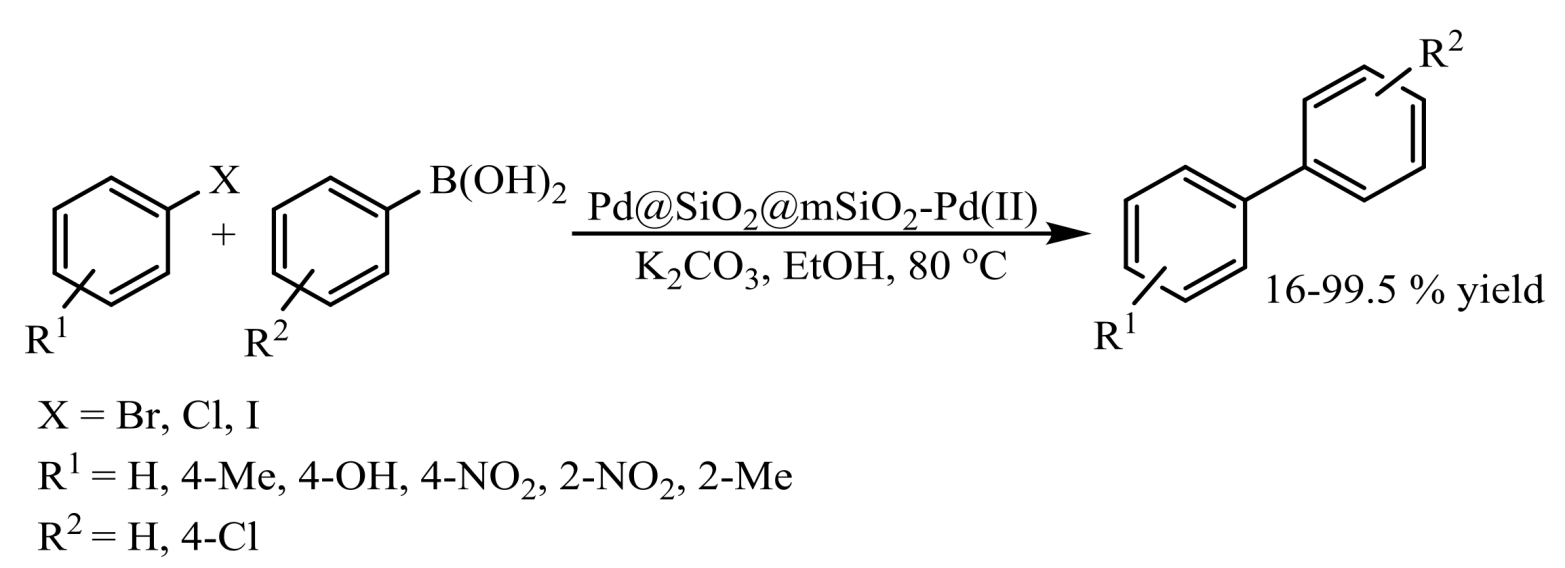
The Suzuki‒Miyaura cross coupling reactions of aryl halides with arylboronic acids using the Fe_3_O_4_@SiO_2_@mSiO_2_-Pd(II) catalyst [66].

Suzuki‒Miyaura cross-coupling reaction between aryl halides and arylboronic acids with 0.5 mol % of obtained catalyst, in the presence of K_2_CO_3_ in EtOH at 80 °C, resulted in the preparation of biaryls products in excellent yields (Scheme 84). Fe_3_O_4_@SiO_2_@mSiO_2_-Pd(II) acted as an efficient and reusable catalyst that could provide high conversion even after six catalytic runs. 

**Scheme 85:**
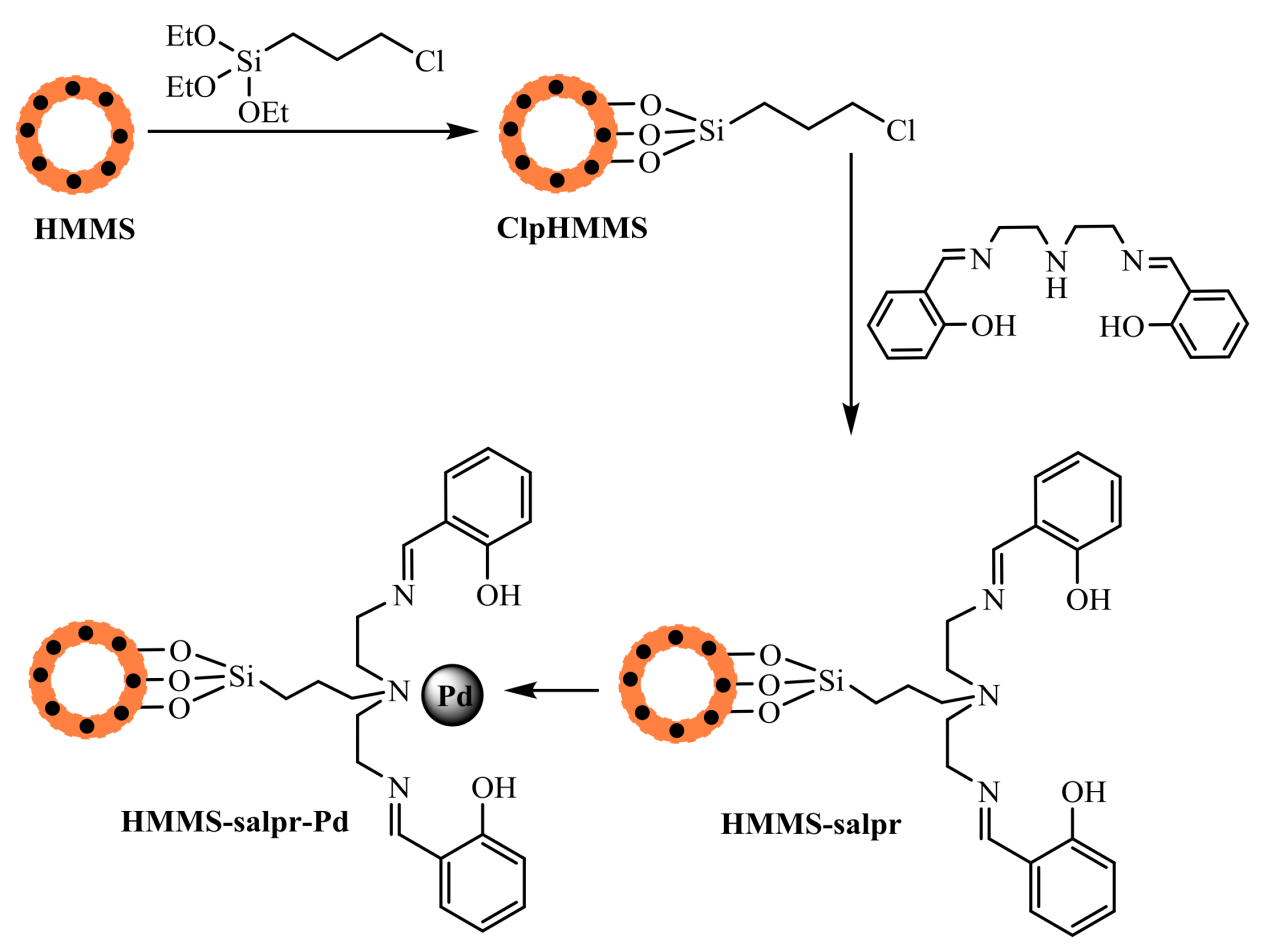
Preparation of the catalysts [67].

**Figure 25:**
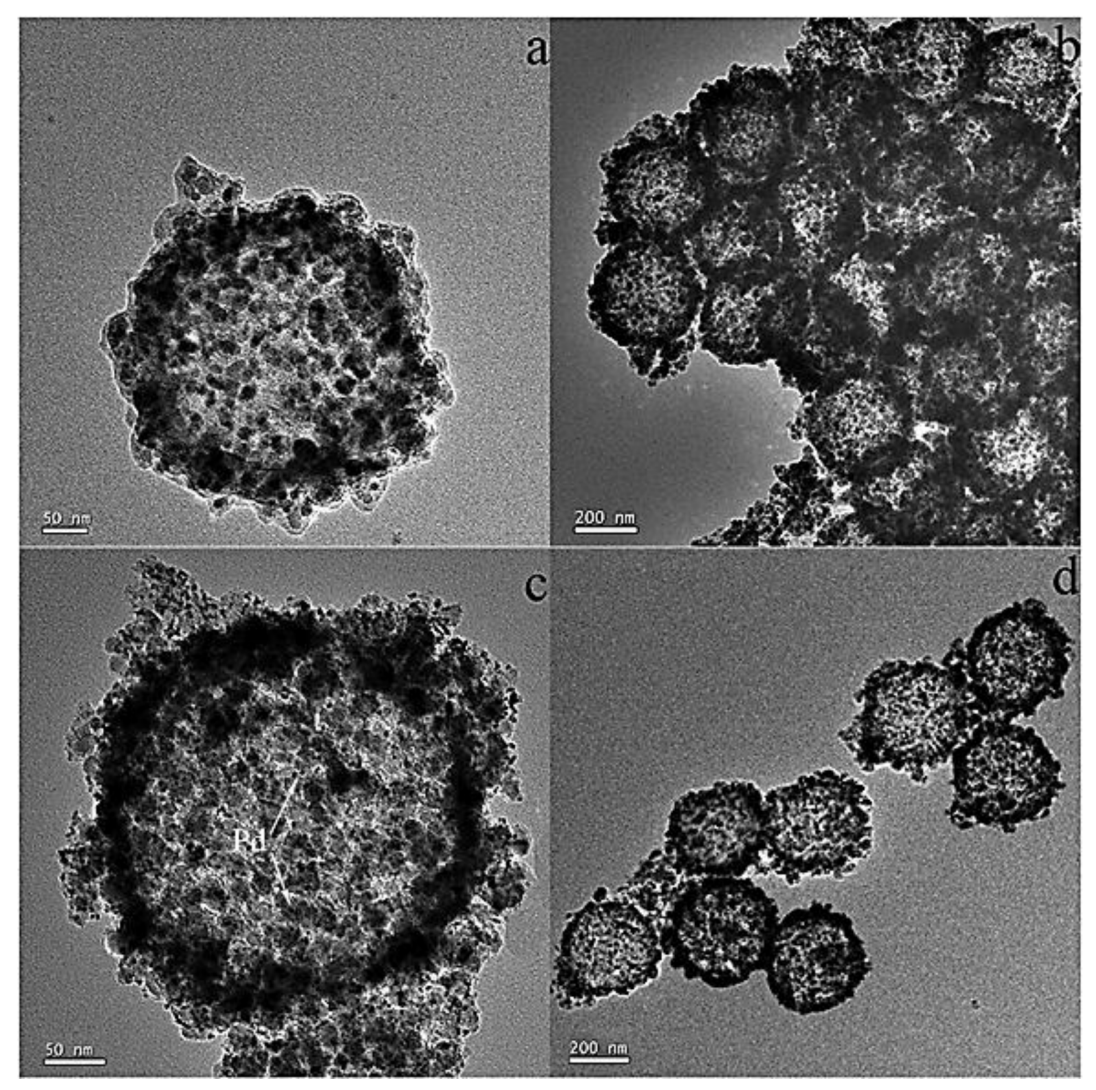
TEM images of (**a**,**b**) HMMS and (**c**) HMMS-salpr-Pd and (**d**) HMMS-salpr-Pd after six reuses [67].

Preparation of hollow magnetic mesoporous spherical catalyst was achieved by Ma and co-workers [67]. They anchored the Schiff base ligand, *N*,*N′*-bis(3-salicylidenaminopropyl)amine (salpr), on the surface of the hollow magnetic mesoporous spheres (HMMS) and finished the complex formation by treatment of the resulting materials with PdCl_2_ (Scheme 85). The TEM images in Figure 25 reveal HMMS with a diameter of about 250 nm via a layer of SiO_2_ with a thickness of about 40 nm. A TEM image of the HMMS-salpr-Pd showed palladium NPs with an average size of about 5 nm on the surface of a hollow magnetic silica sphere.

**Scheme 86:**
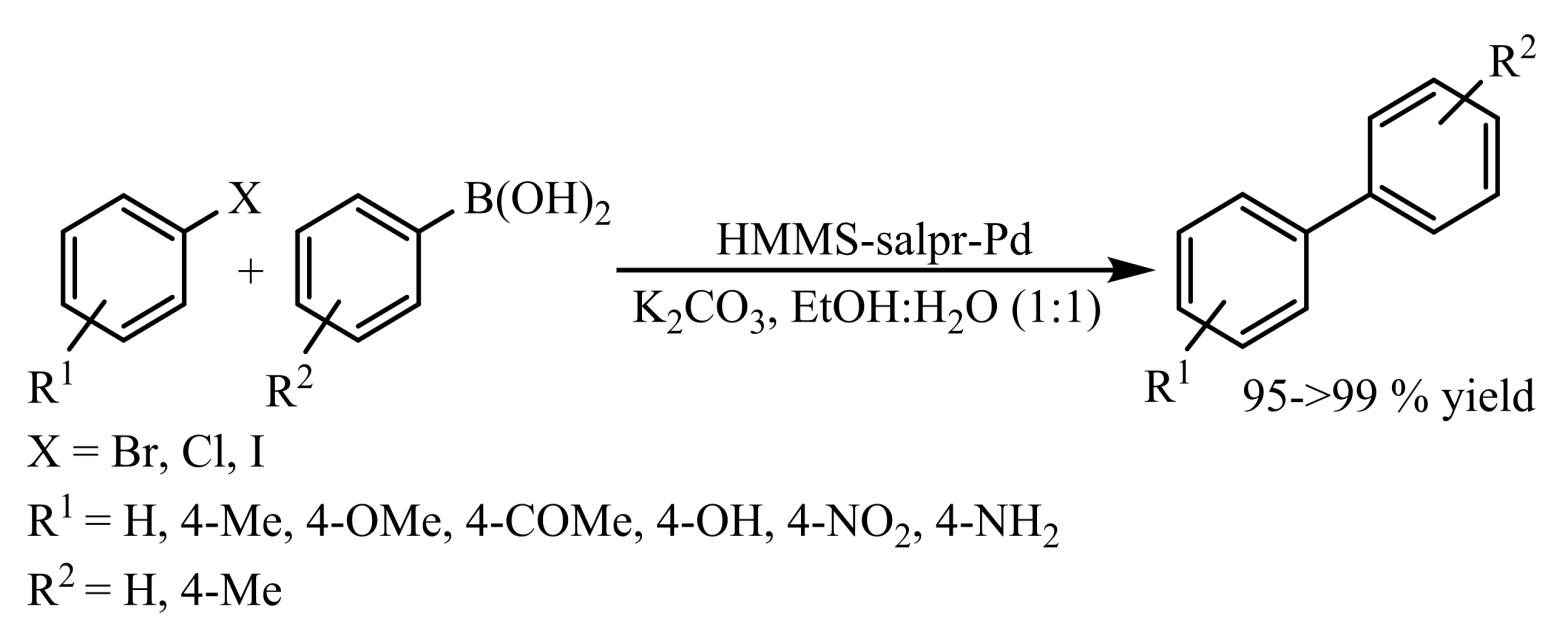
HMMS-salpr-Pd catalyst for Suzuki‒Miyaura coupling reaction [67].

A magnetic mesoporous catalyst was tested for Suzuki‒Miyaura coupling reactions and showed very good to excellent yields (Scheme 86). After the transformations, HMMS-salpr-Pd was recovered simply using an external magnet and could be recycled six times without any loss of activity.

**Scheme 87:**
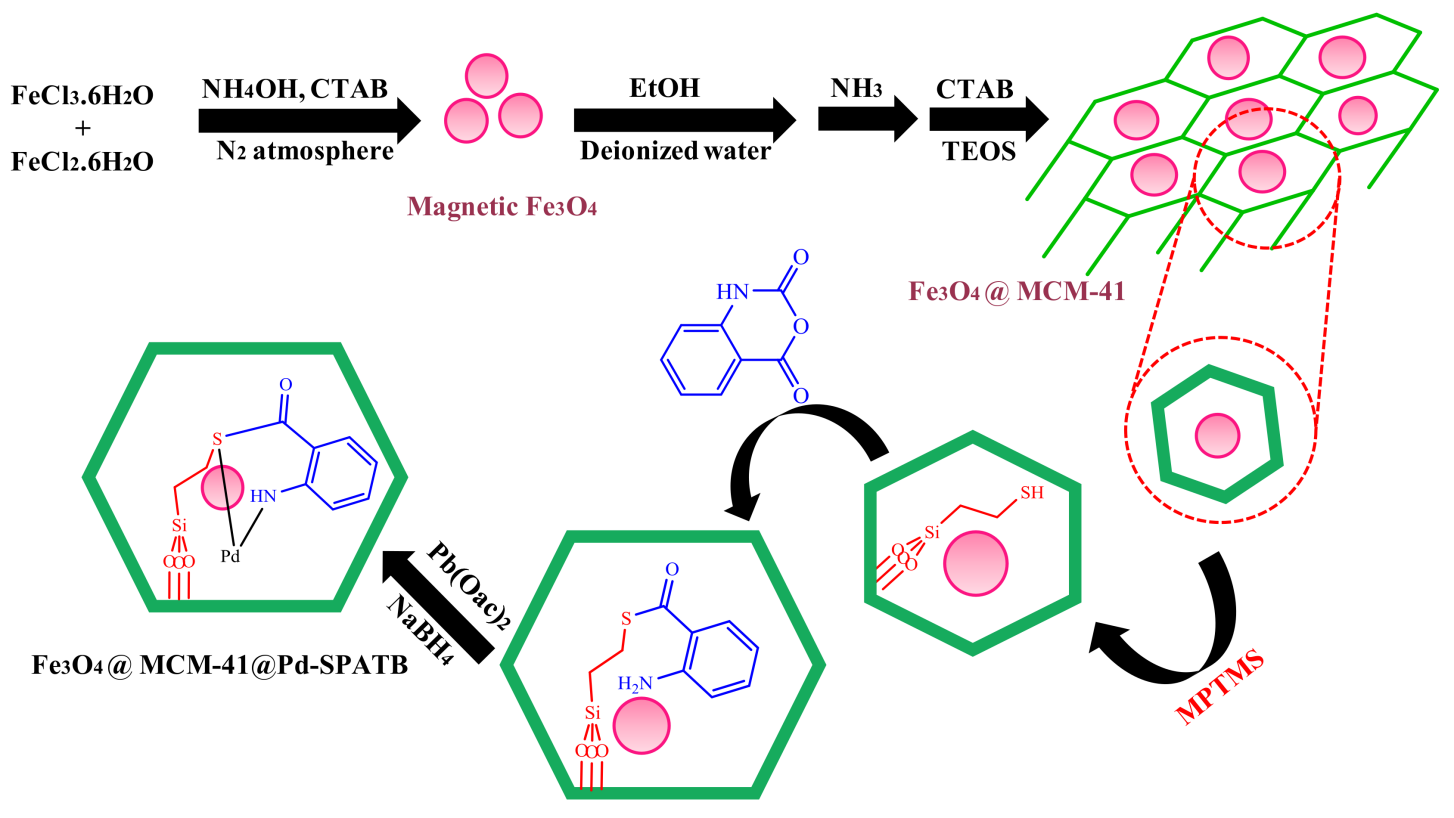
Synthesis of Fe_3_O_4_@MCM-41@Pd-SPATB. Reproduced with permission from [68].

Nikoorazm and co-workers prepared a Fe_3_O_4_@MCM-41@Pd-SPATB catalyst by immobilizing Pd(0)-S-propyl-2-aminobenzothioate complex onto functionalized magnetic nanoporous MCM-41 (Scheme 87) [68].

**Scheme 88:**
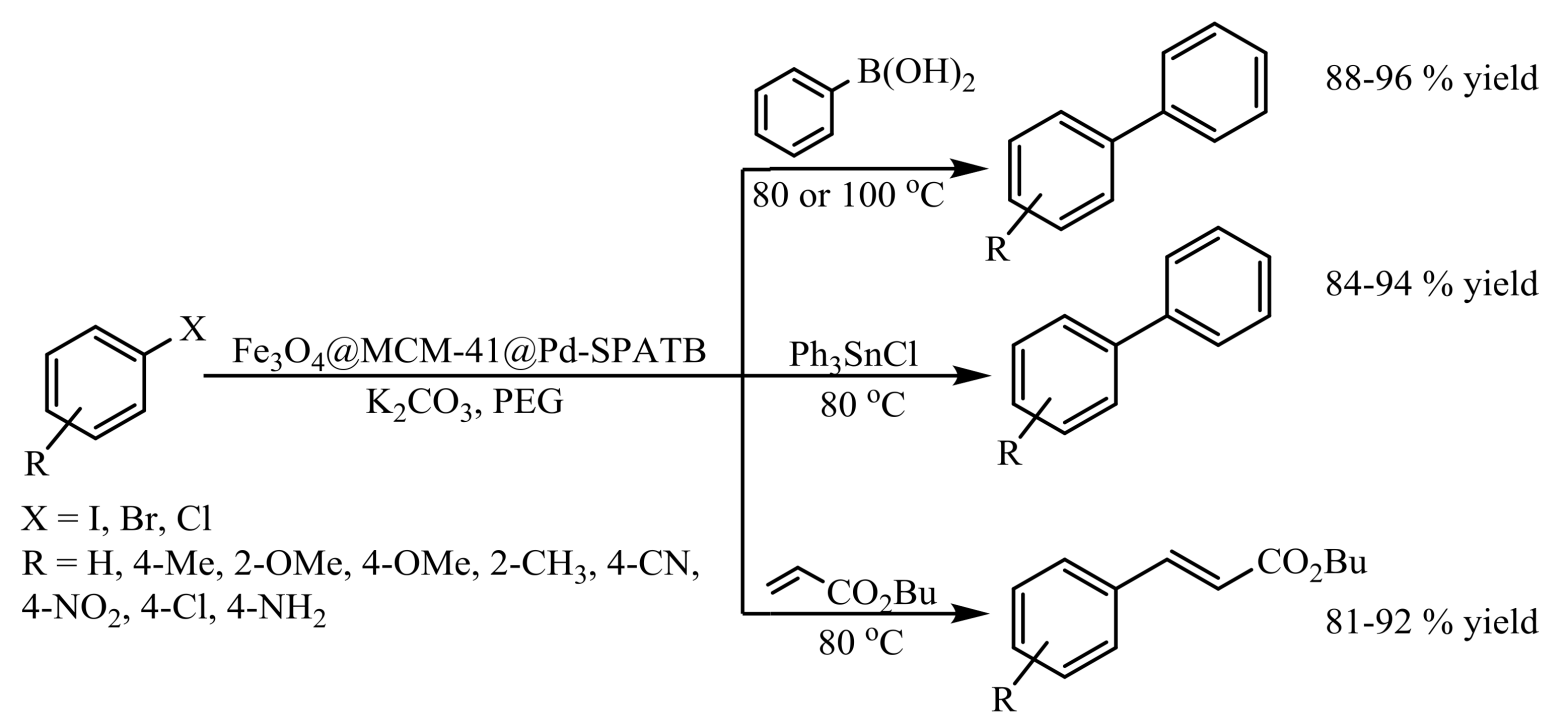
Catalytic activity of Fe_3_O_4_@MCM-41@Pd-SPATB in Suzuki‒Miyaura, Still, and Heck cross-coupling reactions [68].

The resulting nano-organometallic catalyst was tested with various substrates for Suzuki‒Miyaura, Still, and Heck couplings in PEG-400 as green solvent. The best result in Suzuki‒Miyaura reaction of iodobenzene with phenylboronic acid was obtained with K_2_CO_3_ as the base at 80 °C. Meanwhile, completion of the reaction for less reactive aryl chlorides requires a higher temperature (100 °C). Under the optimized conditions, aryl halides coupled with phenylboronic acid and the biphenyl derivatives were obtained in high yields and a short reaction time. In the case of a Still reaction of iodobenzene and Ph_3_SnCl, K_2_CO_3_ was selected as the proper base at 80 °C. After optimization, aryl halides coupled with Ph_3_SnCl in excellent yields. Finally, high yield and high conversion for Heck reaction of iodobenzene and *n*-butyl acrylate were obtained in the presence of K_2_CO_3_ as the base at 120 °C. Under the optimized conditions, aryl halide derivatives coupled with *n*-butyl acrylate in high yields (Scheme 88). In addition, the catalyst can be easily separated from the reaction mixture by applying an external magnet and can be reused for five sequential runs with no remarkable loss of stability and activity.

## 3. Conclusions

Magnetic nanocatalysts have emerged as valuable catalysts for a number of catalytic transformations. Preparing Pd complexes supported on magnetic nanoparticles introduces new possibilities for catalysis due to their excellent properties such as reusability and thermal stability. We have seen that Pd complexes can interact with some magnetic nanoparticles and are able to catalyze a widespread number of C‒C and C‒X coupling reactions that require the presence of transition metals such as palladium. In this review, convenient methods of anchoring or immobilizing Pd complexes on the magnetic supports, magnetic polymer, and porous nanocomposites are investigated in order to synthesize recoverable magnetic nanocatalysts and achieve their characterization. Directed functionalization of the magnetic nanomaterial surfaces is an effective way for Pd complexes heterogenization for magnetic separation. These nanocatalysts showed excellent catalytic performance in C‒X and C‒C coupling reactions and can be recycled from the reaction mixture and reused several times without a marked loss of catalytic activity.

Reported methods for the C‒X and C‒C coupling reactions using magnetic nanocatalysts suffer from drawbacks such as the use of toxic solvents, expensive materials, harsh reaction conditions, low yields, and long reaction times. Therefore, it is desirable to devise simple and efficient methods for the C‒X and C‒C coupling reactions using safer and cheaper magnetic nanocatalysts under aqueous reaction conditions, in ethanol or a H_2_O:EtOH mixture.

Nowadays, researchers are working on finding novel synthetic routes to improve magnetic catalytic systems. Moreover, these magnetic systems can be ideal for the immobilization of other transition metals. The sustainable synthesis of magnetic nanocatalysts using less toxic and more readily available reagents as well as environmentally benign solvents or supports under ambient conditions will also make this field of chemical research more “green.” Thus, the chance to prepare new magnetic nanocatalysts with excellent catalytic activity is an issue still open to researchers to improve the coupling reactions in the future.
